# Mobility Patterns in Different Age Groups in Japan during the COVID-19 Pandemic: a Small Area Time Series Analysis through March 2021

**DOI:** 10.1007/s11524-021-00566-7

**Published:** 2021-08-11

**Authors:** Shuhei Nomura, Yuta Tanoue, Daisuke Yoneoka, Stuart Gilmour, Takayuki Kawashima, Akifumi Eguchi, Hiroaki Miyata

**Affiliations:** 1 Department of Health Policy and Management, School of Medicine, Keio University, 35 Shinanomachi, Shinjuku-ku, Tokyo, 160-8582 Japan; 2 Department of Global Health Policy, Graduate School of Medicine, The University of Tokyo, Tokyo, Japan; 3 Institute for Business and Finance, Waseda University, Tokyo, Japan; 4 Graduate School of Public Health, St. Luke’s International University, Tokyo, Japan; 5 Department of Mathematical and Computing Science, Tokyo Institute of Technology, Tokyo, Japan; 6 Center for Preventive Medical Sciences, Chiba University, Chiba, Japan

**Keywords:** Japan, COVID-19, Human mobility, Smartphone

## Abstract

In the COVID-19 era, movement restrictions are crucial to slow virus transmission and have been implemented in most parts of the world, including Japan. To find new insights on human mobility and movement restrictions encouraged (but not forced) by the emergency declaration in Japan, we analyzed mobility data at 35 major stations and downtown areas in Japan—each defined as an area overlaid by several 125-meter grids—from September 1, 2019 to March 19, 2021. Data on the total number of unique individuals per hour passing through each area were obtained from Yahoo Japan Corporation (i.e., more than 13,500 data points for each area). We examined the temporal trend in the ratio of the rolling seven-day daily average of the total population to a baseline on January 16, 2020, by ten-year age groups in five time frames. We demonstrated that the degree and trend of mobility decline after the declaration of a state of emergency varies across age groups and even at the subregional level. We demonstrated that monitoring dynamic geographic and temporal mobility information stratified by detailed population characteristics can help guide not only exit strategies from an ongoing emergency declaration, but also initial response strategies before the next possible resurgence. Combining such detailed data with data on vaccination coverage and COVID-19 incidence (including the status of the health care delivery system) can help governments and local authorities develop community-specific mobility restriction policies. This could include strengthening incentives to stay home and raising awareness of cognitive errors that weaken people's resolve to refrain from nonessential movement.

**Supplementary Information:**

The online version contains supplementary material available at 10.1007/s11524-021-00566-7.

## Introduction

One month after World Health Organization declared the new coronavirus (COVID-19) outbreak as a pandemic on 11 March 2020, an estimated 4.5 billion people worldwide were under some form of lockdown or movement restriction in response to COVID-19. COVID-19 can be transmitted through direct or indirect human contact via droplet and/or particle transmission, and in the absence of vaccines non-pharmaceutical interventions (NPIs) that restrict social contact were an essential strategy for containing the pandemic [1, 2]. Movement restriction is a promising NPI because it is known to reduce the chance and intensity of human contact, both through reducing opportunities for social gatherings and by reducing social contact during commuting, a particularly important potential transmission route in dense cities like Tokyo with widespread public transport use [3]. Studies have shown that it is associated with lower virus transmission rates [4], and also correlates well with decreases in COVID-19 incidence [5–7]. In Japan, a state of emergency was issued for seven prefectures including Tokyo on April 7, 2020, extended to 47 prefectures nationwide on April 16, and lifted in stages by May 25, and the government uniformly called for citizens to refrain from nonessential movement, and encouraged remote work [8]. Japan's emergency declaration was unique in that it was not legally binding; compliance was voluntary. In response to the resurgence of COVID-19, a second state of emergency was declared on January 7, 2021 in four prefectures including Tokyo (extended to 11 prefectures on January 13, 2021), and the government once again asked citizens to refrain from nonessential movement [9].

Smartphones can serve as a tool for monitoring the situation regarding the spread of COVID-19 infections in a region based on the reported COVID-19-like illness symptoms and location information provided by smartphone users [10]. They also provide dynamic data on population movement—information on where people are moving from one place to another. These data will provide important insights into people's mobility patterns, helping to better understand the level of human contact and thus the risk of virus transmission across regions, as well as to assess the impact of lockdowns and other restrictions on population movement [11]. Meanwhile, data on human mobility from smartphones is often reported at a high level of aggregation such as national or regional levels [12–15]. Utilization of such data at a smaller area level is more likely to reflect movement-restriction policies within local contexts.

Nearly a year after the initial emergency declaration, self-restraint burnout (or so-called COVID-19 fatigue [16]) is spreading in Japan; future uniform calls for movement restraint will not be as effective as they have been in the past [17]. The practicalities of daily living vary greatly depending on individual and regional characteristics. In light of this fatigue, future movement-restriction policies may need to be tailored to different population groups with clarity on what specific parts of daily life require movement restraint.

In this study, we aim to explore in detail the human mobility patterns during the COVID-19 pandemic by age group and time frame in 35 areas including major stations across Japan, using data collected from smartphones. We statistically, examined whether the human mobility score decreased after each of the two emergency declarations, whether there was a difference in the rate of decrease among age groups, and further examined the cross-correlation between human mobility score and the number newly reported COVID-19 cases. By evaluating dynamic, geographic, and temporal movement information stratified by detailed population characteristics, we hope that this study will contribute to exit strategies from the ongoing emergency declarations, as well as initial response strategies before the next possible resurgence.

## Methods

We analyzed mobility data collected by Yahoo Japan Corporation. Yahoo Japan owns the location data of more than 80 million users who have authorized the use of GPS-based location information in various smartphone applications provided by the company, accounting for more than 60% of the total population in Japan. This mobility data is not publicly available, and we obtained it from Yahoo Japan for exclusive academic use based on an agreement between Keio University and Yahoo Japan for 35 major stations and downtown areas in Japan that we defined (see below), from September 1, 2019 to March 19, 2021. The data was anonymized by Yahoo Japan prior to our access, by aggregating the data by ten-year age groups on the total number of unique individuals per hour passing through each area (i.e., more than 13,500 data points for each area). See Table S1 in the supplementary material for the detailed definitions and other information of the 35 areas, and the geographical locations of each area are also provided in Figure S1. For example, one of the areas includes the Tokyo Station. Yahoo Japan calculates population numbers using a minimum grid of 125 meters as defined by the Ministry of Internal Affairs and Communications. We defined the Tokyo Station area to be overlaid on 9 grids that includes the entire Tokyo Station. Yahoo Japan then counted the unique individuals who have passed through (or already been in) the area covered by those 9 grids at a certain time as the number of people in the Tokyo Station area at that time (by age group). We only receive the data on that number. The 35 areas are all the same areas that are monitored by the Office for Novel Coronavirus Disease Control, Cabinet Secretariat, Government of Japan [18], and we selected them in light of the direct applicability of the results of this study to current policies and for their replicability by the government. In the present study, we investigated the data for the 35 areas, by five time frames. We decided to split the time frame because we thought that people's mobility patterns should be very different at different time frames in a day.

First, we plotted the ratio of the rolling seven-day daily average of the total population to a baseline on January 16, 2020 (hereafter referred to human mobility score), through the end of the study period. The baseline was also the rolling seven-day daily average. This baseline date was chosen because it was the day the media reported the first case of infection in Japan and we thought that it was the time when the 2020 New Year holidays and consecutive holidays were over and people resumed their relatively normal daily lives. Second, to investigate whether human mobility score decreased after the declaration of a state of emergency, the ratio during the declaration was compared to those for the same period before the declaration, using the nonparametric Wilcoxon signed-rank test, by age groups and time frames, and by the first and second declaration. Third, to investigate whether the degree of the reduction of human mobility score during the declaration differed by age groups, we compared the ratios during each of the first and second emergency declarations among age groups using a nonparametric Kruskal-Wallis test, by time frames. Finally, using the Haugh-Box test, we examined zero cross-correlation across all maximum +10-day lags between the daily number of newly reported COVID-19 cases with the human mobility score during the declaration of a state of emergency, by age groups and time frames, and by the first and second declaration. For the data of the number of COVID-19 cases, given its availability, we considered daily prefecture-level figures for comparisons [19].

## Results

**Fig. 1.**
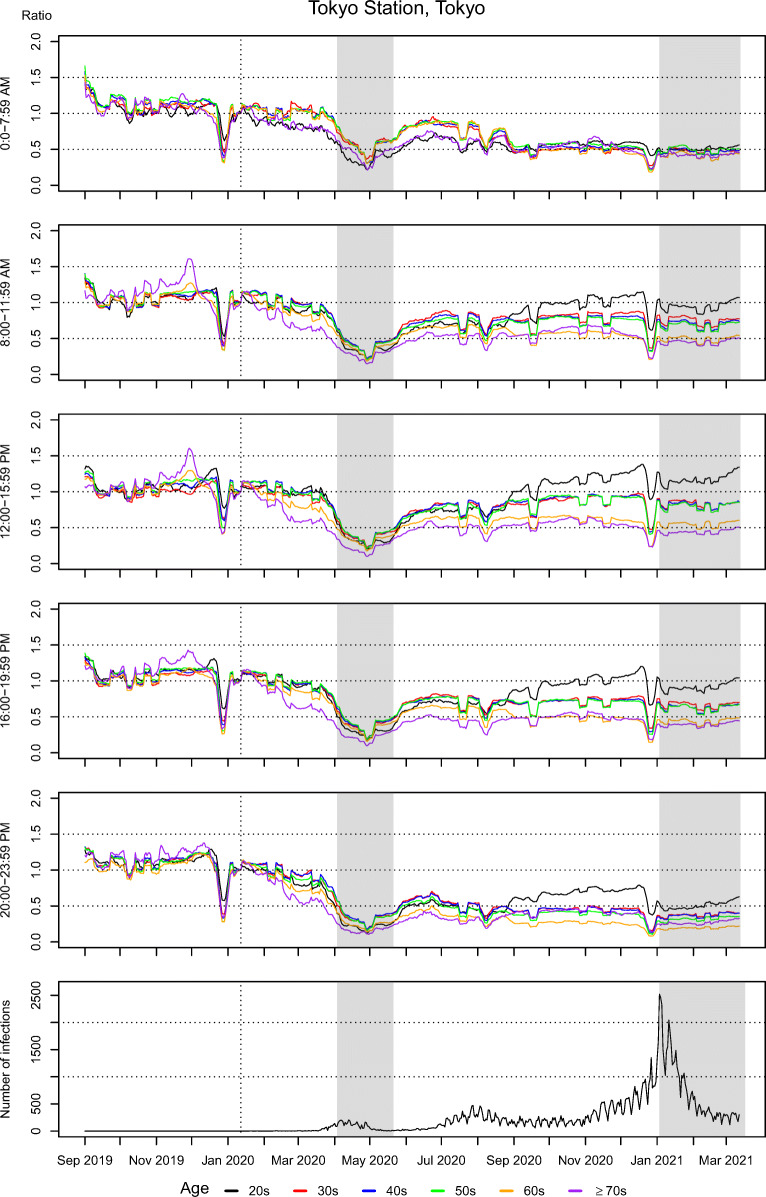
Temporal trends in human mobility in the Tokyo Station area by age groups and time frames and the daily number of newly reported COVID-19 cases in the area. The top five graphs show the ratio of the rolling seven-day daily average of the total population based on different time frames, and the last one is a graph of the daily number of newly reported COVID-19 cases. Gray areas indicate a state of emergency

Figure 1 shows the temporal trend in the ratio of the rolling seven-day daily average of the total population to a baseline on January 16, 2020, in the Tokyo Station area, by age groups and time frame. There are five graphs showing the ratio of the rolling seven-day daily average of the total population based on different time frames. Those for the other 34 areas are presented in Figure S2 in the supplementary material. We observed a statistically significant decrease in human mobility score after the first declaration of the state of emergency at almost all time frames, all areas, and all age groups (Table S2). After the second declaration of the state of emergency, we also found that human mobility score decreased in many cases, but in some cases, especially for people in their 60s, the decrease was not statistically significant. There were also statistically significant differences in the mobility score reduction among age groups for all time frames and all areas for both the first and second emergency declarations (Table S3). These results suggest significant mobility reduction of varying degrees among different age groups. The cross-correlations between the daily number of newly reported COVID-19 cases with the human mobility score also varied by area, time frame, and age group (Table S4).

The trend patterns after the first declaration of a state of emergency through the study end point obviously vary by age groups and time frames, and they also differ greatly across the 35 areas. In some cases, mobility score reduction had already started in March, 2020, before the first emergency declaration and there was a recovery of mobility after it that could be partially related to self-restraint burnout, and/or indicated a return to in-office working. In other cases, the ratio remained below 50% of the baseline for a long time after the first emergency declaration.

## Discussion

Our analyzes indicated that human mobility data from smartphone users can provide information on varying degrees of movement restraint [11, 12]. Several previous studies conducted in Japan have shown its usefulness as well. Yabe et al. showed that the first declaration of a state of emergency reduced human mobility by nearly 50% in Tokyo Prefecture, using mobility data collected from smartphones [13]. Similarly, Arimura et al. demonstrated that the declaration reduced human mobility by up to nearly 90% in Sapporo City (Hokkaido Prefecture), depending on the place and time frame of a day [20]. Nagata et al., who tracked human mobility in Tokyo, Osaka, and Aichi Prefectures using smartphone data, pointed out that the reduction in human mobility started before the first declaration of the state of emergency, and that the degree of reduction varied from place to place [21]. Furthermore, Kajitani et al. found that a 20–35% reduction in mobility may have been necessary to hold back the pandemic (i.e., to reduce the effective reproduction number to one or less) in the situation during the first wave of the pandemic in the business and commercial districts of nine prefectures including Tokyo [22]. These studies did not identify age-specific human mobility patterns as an analytical limitation, but we were able to show that the degrees of mobility reduction as well as the trend patterns after the emergency declarations varied among different age groups, and further varied at the small area level.

Monitoring dynamic geographic or temporal mobility information stratified by detailed population characteristics could help guide not only exit strategies from the on-going emergency declaration (e.g., when, where, who, and how to ease restrictions while balancing other concerns), but also initial response strategies before the next possible resurgence arrives in a tailored manner. Such mobility monitoring may be key to early detection of any new wave of COVID-19 since the mobility is known to correlate well with COVID-19 incidence and social contact [23–25]. Combining such data with vaccination coverage information and COVID-19 incidence data, including the status of health care delivery systems, would allow governments and local authorities to formulate localized movement-restriction policies, including strengthening incentives to stay at home and raising awareness of cognitive errors (e.g., omission, optimism, and confirmation biases) that weaken one's resolve to refrain from nonessential movement [26].

This study also implies that socioeconomic, psychological, or other factors that characterize a change in human mobility may vary by different population groups and time frames, even in a small area [27–29]. Therefore, the movement-restriction policies will be more effective if the policies are clearly targeted to a specific population group at a specific time. Governments and local authorities responsible for movement-restrictions for their respective populations should seek to replicate our work with a view to understand the characteristics of population with changing mobility within their own context, and formulate policies that specifically target these populations. A multidisciplinary approach engaging social and behavioral change communication experts, social influencers, anthropologists, behavioral economists, and psychologists is likely to be required.

We emphasize that contextualization is essential for fair interpretation of human mobility data. For example, in regions and age groups with many occupational types where working remotely is difficult, the degree of mobility reduction is inevitably smaller since travel to the workplace is unavoidable [30]. In addition, the release of human mobility data requires careful messaging and communication to protect individual privacy, safety, and trust, as well as to prevent unintended consequences, such as discrimination and prejudice against groups that have difficulty limiting their mobility for physical, health, or other social reasons [12]. However, careful use of mobility data from ubiquitous modern smartphone devices, paired with appropriate health system information, could enable the development of more locally tailored and effective movement-restriction policies which minimize disruption of daily or economic life.

## Supplementary Information

ESM 1 (PDF 5.56 mb)
