# Supplementary material for: Mobility Patterns in Different Age Groups in Japan during the COVID-19 Pandemic: a Small Area Time Series Analysis through March 2021
Source: J Urban Health. 2021 Aug 11;98(5):635–41. doi: 10.1007/s11524-021-00566-7 (PMC8356689; doi:10.1007/s11524-021-00566-7)
Supplement: Supplementary file 1 — (PDF 5.56 mb) [file 11524_2021_566_MOESM1_ESM.pdf]

Table S1: Area information. An area was defined as the grid containing the center point of each location and covering its surrounding grids. The table below for the number of grids considered and other area information.

Area	Prefecture	Number of grids considered	Average number of passengers getting on and off per day at the station in 2017 *	Date of the declaration of a state of emergency	
				First	Second
Susukino Station	Hokkaido	9	39,752	April 16, 2020	
Odori Station	Hokkaido	9	159,694	April 16, 2020	
Central Hirosaki Station	Aomori	9	767	April 16, 2020	
Kotodai-Koen Station	Miyagi	9	33,646	April 16, 2020	
Tobu-Utsunomiya station	Tochigi	9	9,894	April 16, 2020	January 13, 2021
Chuo Maebashi Station	Gunma	9	1,702	April 16, 2020	
Shibuya Station	Tokyo	14	224,784	April 7, 2020	January 7, 2021
Shibuya Center Street	Tokyo	9	NA / Shibuya Center Street is one of the biggest downtown areas in Tokyo.	April 7, 2020	January 7, 2021
Harajuku Station	Tokyo	9	152,168	April 7, 2020	January 7, 2021
Shinjuku Kabukicho	Tokyo	9	NA / Shinjuku Kabukicho is one of Tokyo's largest entertainment districts.	April 7, 2020	January 7, 2021
Ikebukuro Station	Tokyo	9	568,316	April 7, 2020	January 7, 2021
Tokyo Station	Tokyo	9	1,090,666	April 7, 2020	January 7, 2021
Shinbashi Station	Tokyo	14	958,018	April 7, 2020	January 7, 2021
Shinagawa Station	Tokyo	9	1,028,462	April 7, 2020	January 7, 2021
Ueno Station	Tokyo	9	578,406	April 7, 2020	January 7, 2021
Ginza Station	Tokyo	9	266,574	April 7, 2020	January 7, 2021
Roppongi Station	Tokyo	9	235,489	April 7, 2020	January 7, 2021
Kichijoji Station	Tokyo	9	430,717	April 7, 2020	January 7, 2021
Motomachi-Chukagai Station	Kanagawa	9	62,659	April 7, 2020	January 7, 2021
Sakuragicho Station	Kanagawa	9	178,396	April 7, 2020	January 7, 2021
Kannai Station	Kanagawa	9	155,171	April 7, 2020	January 7, 2021
Hamamatsu Station	Shizuoka	9	73,512 **	April 16, 2020	
Sakae Station	Aichi	9	230,805	April 16, 2020	January 13, 2021

Fushimi Station	Aichi	9	96,176	April 16, 2020	January 13, 2021
Kawaramachi Station	Kyoto	9	78,477	April 16, 2020	January 13, 2021
Shinsaibashi Station	Osaka	9	148,155	April 7, 2020	January 13, 2021
Namba Station	Osaka	9	607,584	April 7, 2020	January 13, 2021
Kitashinchi Station	Osaka	9	101,577	April 7, 2020	January 13, 2021
Tamachi Station	Okayama	9	122	April 16, 2020	
Hatchobori Station	Hiroshima	9	14,148	April 16, 2020	
Kawaramachi Station	Kagawa	9	14,223	April 16, 2020	
Hakata Station	Fukuoka	9	199,839	April 7, 2020	January 13, 2021
Nakasu-kawabata Station	Fukuoka	9	37,054	April 7, 2020	January 13, 2021
Kanko-dori Station	Nagasaki	9	2,500	April 16, 2020	
Makishi Station	Okinawa	9	7,181	April 16, 2020	

* Source: Ministry of Land, Infrastructure, Transport and Tourism. Number of passengers by station. https://nlftp.mlit.go.jp/ksj/gml/datalist/KsjTmplt-S12-v2_3.html (accessed May 16, 2021).

** Source: Hamamatsu City. Statistics: Hamamatsu City in 2017 [Japanese]. 2018. https://www.city.hamamatsu.shizuoka.jp/gyousei/library/toukeisyo/004_h29-toukeisyo.html (accessed May 17, 2021). The number was calculated by doubling the average number of passengers getting on per day.

Table S2: P-values for the comparison of human mobility before and during the declaration of a state of emergency using the Wilcoxon signed-rank test, by age groups and time frames, and by the first and second declaration. The ratios of the rolling seven-day daily average of the total population to a baseline on January 16, 2020, were compared between during the declaration of a state of emergency and during the same period before the declaration.

				Median ratio (before and during the emergency declaration: B, D) by age group											
Declaration	Time frame	Area	Prefecture	B, D	20s	B, D	30s	B, D	40s	B, D	50s	B, D	60s	B, D	≥ 70s
First															
	0:00–7:59 AM														
		Susukino Station	Hokkaido	0.89, 0.33	<0.001	0.56, 0.27	<0.001	0.63, 0.34	<0.001	0.71, 0.37	<0.001	0.62, 0.36	<0.001	0.72, 0.53	<0.001
		Odori Station	Hokkaido	0.78, 0.46	<0.001	1.03, 0.73	<0.001	1.04, 0.69	<0.001	1.01, 0.75	<0.001	0.98, 0.67	<0.001	0.89, 0.66	<0.001
		Central Hirosaki Station	Aomori	0.34, 0.05	<0.001	1.40, 0.92	<0.001	1.35, 0.75	<0.001	1.08, 0.64	<0.001	1.34, 0.91	<0.001	0.88, 0.86	0.93
		Kotodai-Koen Station	Miyagi	0.90, 0.51	<0.001	0.70, 0.48	<0.001	0.84, 0.66	<0.001	1.09, 0.73	<0.001	1.11, 0.82	<0.001	0.97, 0.57	<0.001
		Tobu-Utsunomiya station	Tochigi	0.93, 0.54	<0.001	0.91, 0.66	<0.001	0.97, 0.57	<0.001	1.38, 0.76	<0.001	1.05, 0.84	<0.001	0.70, 0.61	<0.01
		Chuo Maebashi Station	Gunma	1.87, 1.86	0.81	0.77, 0.52	<0.001	0.81, 0.56	<0.001	0.88, 0.69	<0.001	0.68, 0.45	<0.001	0.98, 0.65	<0.001
		Shibuya Station	Tokyo	0.85, 0.35	<0.001	0.78, 0.46	<0.001	0.92, 0.60	<0.001	0.89, 0.64	<0.001	0.88, 0.61	<0.001	0.92, 0.59	<0.001
		Shibuya Center Street	Tokyo	0.77, 0.31	<0.001	0.73, 0.41	<0.001	0.86, 0.54	<0.001	0.82, 0.57	<0.001	0.82, 0.54	<0.001	0.85, 0.55	<0.001
		Harajuku Station	Tokyo	0.99, 0.50	<0.001	1.14, 0.58	<0.001	1.16, 0.83	<0.001	1.21, 0.84	<0.001	1.24, 0.88	<0.001	1.43, 1.17	<0.001
		Shinjuku Kabukicho	Tokyo	0.97, 0.30	<0.001	1.05, 0.55	<0.001	1.01, 0.53	<0.001	0.98, 0.60	<0.001	1.04, 0.64	<0.001	0.93, 0.54	<0.001
		Ikebukuro Station	Tokyo	1.09, 0.50	<0.001	1.15, 0.72	<0.001	1.12, 0.76	<0.001	1.19, 0.82	<0.001	1.09, 0.75	<0.001	1.09, 0.69	<0.001
		Tokyo Station	Tokyo	0.79, 0.38	<0.001	1.02, 0.60	<0.001	1.03, 0.60	<0.001	1.01, 0.61	<0.001	0.98, 0.58	<0.001	0.80, 0.45	<0.001
		Shinbashi Station	Tokyo	0.83, 0.37	<0.001	0.99, 0.48	<0.001	0.97, 0.56	<0.001	0.96, 0.50	<0.001	0.94, 0.53	<0.001	0.95, 0.47	<0.001
		Shinagawa Station	Tokyo	0.78, 0.48	<0.001	1.00, 0.60	<0.001	0.92, 0.61	<0.001	0.97, 0.61	<0.001	0.93, 0.62	<0.001	0.68, 0.51	<0.001
		Ueno Station	Tokyo	0.99, 0.74	<0.001	1.07, 0.74	<0.001	1.01, 0.77	<0.001	0.99, 0.78	<0.001	0.92, 0.66	<0.001	0.83, 0.56	<0.001
		Ginza Station	Tokyo	1.04, 0.44	<0.001	1.08, 0.48	<0.001	1.07, 0.58	<0.001	1.03, 0.55	<0.001	0.95, 0.51	<0.001	0.89, 0.40	<0.001
		Roppongi Station	Tokyo	0.93, 0.28	<0.001	1.05, 0.49	<0.001	0.97, 0.62	<0.001	0.98, 0.58	<0.001	0.97, 0.59	<0.001	1.00, 0.52	<0.001
		Kichijoji Station	Tokyo	1.08, 0.74	<0.001	0.92, 0.56	<0.001	1.08, 0.75	<0.001	1.04, 0.72	<0.001	0.99, 0.71	<0.001	1.09, 0.90	<0.001
		Motomachi-Chukagai Station	Kanagawa	1.10, 0.69	<0.001	0.98, 0.80	<0.001	0.96, 0.79	<0.001	0.97, 0.81	<0.001	1.15, 1.01	<0.05	0.80, 0.65	<0.001
		Sakuragicho Station	Kanagawa	0.80, 0.47	<0.001	0.97, 0.79	<0.001	0.96, 0.85	<0.001	0.99, 0.75	<0.001	0.91, 0.70	<0.001	0.74, 0.61	<0.001
		Kannai Station	Kanagawa	1.71, 0.92	<0.001	0.96, 0.72	<0.001	1.15, 0.88	<0.001	1.07, 0.84	<0.001	1.01, 0.74	<0.001	1.56, 1.18	<0.001
		Hamamatsu Station	Shizuoka	0.82, 0.31	<0.001	0.88, 0.61	<0.001	0.78, 0.55	<0.001	0.93, 0.70	<0.001	0.91, 0.71	<0.001	0.92, 0.78	<0.001
		Sakae Station	Aichi	0.69, 0.48	<0.001	0.93, 0.47	<0.001	0.92, 0.51	<0.001	0.95, 0.50	<0.001	0.91, 0.57	<0.001	0.77, 0.41	<0.001

	Fushimi Station	Aichi	0.93, 0.47	<0.001	1.12, 0.66	<0.001	0.96, 0.66	<0.001	1.11, 0.82	<0.001	0.95, 0.71	<0.001	0.69, 0.42	<0.001
	Kawaramachi Station	Kyoto	1.06, 0.29	<0.001	0.72, 0.39	<0.001	0.88, 0.54	<0.001	1.04, 0.57	<0.001	1.02, 0.51	<0.001	0.95, 0.71	<0.001
	Shinsaibashi Station	Osaka	0.93, 0.32	<0.001	0.81, 0.43	<0.001	0.84, 0.54	<0.001	0.87, 0.53	<0.001	0.92, 0.56	<0.001	0.68, 0.53	<0.001
	Namba Station	Osaka	1.17, 0.35	<0.001	0.85, 0.38	<0.001	0.90, 0.52	<0.001	0.92, 0.52	<0.001	0.85, 0.50	<0.001	0.79, 0.43	<0.001
	Kitashinchi Station	Osaka	0.92, 0.27	<0.001	0.93, 0.33	<0.001	0.93, 0.38	<0.001	1.02, 0.46	<0.001	1.02, 0.43	<0.001	1.01, 0.46	<0.001
	Tamachi Station	Okayama	0.98, 0.68	<0.001	0.77, 0.42	<0.001	1.28, 0.81	<0.001	1.14, 0.70	<0.001	0.93, 0.63	<0.001	0.66, 0.69	<0.05
	Hatchobori Station	Hiroshima	0.71, 0.32	<0.001	0.65, 0.37	<0.001	0.79, 0.47	<0.001	0.72, 0.41	<0.001	0.78, 0.45	<0.001	0.99, 0.38	<0.001
	Kawaramachi Station	Kagawa	0.88, 0.47	<0.001	1.32, 0.84	<0.001	1.22, 1.07	0.14	1.69, 1.63	0.60	1.51, 1.46	0.36	1.17, 1.45	<0.01
	Hakata Station	Fukuoka	0.86, 0.35	<0.001	0.86, 0.46	<0.001	1.04, 0.58	<0.001	0.93, 0.51	<0.001	0.87, 0.50	<0.001	0.85, 0.46	<0.001
	Nakasu-kawabata Station	Fukuoka	0.83, 0.48	<0.001	0.70, 0.29	<0.001	0.80, 0.39	<0.001	0.94, 0.53	<0.001	0.97, 0.54	<0.001	0.76, 0.40	<0.001
	Kanko-dori Station	Nagasaki	1.33, 0.73	<0.001	1.15, 0.69	<0.001	0.86, 0.58	<0.001	0.89, 0.74	<0.001	1.02, 0.82	<0.001	0.83, 0.61	<0.001
	Makishi Station	Okinawa	1.25, 0.85	<0.001	0.82, 0.60	<0.001	1.24, 1.07	<0.001	0.97, 0.73	<0.001	1.15, 0.91	<0.001	1.29, 0.85	<0.001
08:00−11:59 AM														
	Susukino Station	Hokkaido	1.05, 0.65	<0.001	0.97, 0.67	<0.001	0.95, 0.70	<0.001	0.96, 0.73	<0.001	0.89, 0.63	<0.001	0.81, 0.60	<0.001
	Odori Station	Hokkaido	0.90, 0.51	<0.001	1.06, 0.69	<0.001	0.98, 0.66	<0.001	0.97, 0.64	<0.001	0.91, 0.58	<0.001	0.74, 0.49	<0.001
	Central Hirosaki Station	Aomori	0.54, 0.49	<0.01	1.13, 0.84	<0.001	1.18, 0.78	<0.001	1.17, 0.87	<0.001	1.39, 1.12	<0.001	1.00, 0.91	<0.001
	Kotodai-Koen Station	Miyagi	0.82, 0.56	<0.001	1.09, 0.77	<0.001	0.98, 0.69	<0.001	1.03, 0.72	<0.001	1.00, 0.71	<0.001	0.87, 0.62	<0.001
	Tobu-Utsunomiya station	Tochigi	1.11, 0.80	<0.001	1.08, 0.76	<0.001	0.99, 0.64	<0.001	1.15, 0.76	<0.001	0.97, 0.72	<0.001	0.84, 0.51	<0.001
	Chuo Maebashi Station	Gunma	1.70, 1.63	<0.01	0.45, 0.39	<0.001	0.74, 0.62	<0.001	0.96, 0.79	<0.001	0.66, 0.54	<0.001	1.00, 0.81	<0.001
	Shibuya Station	Tokyo	0.75, 0.32	<0.001	0.82, 0.44	<0.001	0.84, 0.50	<0.001	0.88, 0.55	<0.001	0.81, 0.48	<0.001	0.67, 0.38	<0.001
	Shibuya Center Street	Tokyo	0.68, 0.29	<0.001	0.75, 0.41	<0.001	0.78, 0.46	<0.001	0.81, 0.50	<0.001	0.75, 0.44	<0.001	0.61, 0.36	<0.001
	Harajuku Station	Tokyo	0.82, 0.38	<0.001	0.96, 0.57	<0.001	0.92, 0.57	<0.001	0.97, 0.66	<0.001	0.86, 0.60	<0.001	0.80, 0.55	<0.001
	Shinjuku Kabukicho	Tokyo	0.82, 0.40	<0.001	0.88, 0.51	<0.001	0.86, 0.52	<0.001	0.86, 0.53	<0.001	0.82, 0.48	<0.001	0.63, 0.36	<0.001
	Ikebukuro Station	Tokyo	0.96, 0.47	<0.001	1.02, 0.59	<0.001	1.06, 0.65	<0.001	1.08, 0.68	<0.001	1.01, 0.59	<0.001	0.86, 0.45	<0.001
	Tokyo Station	Tokyo	0.88, 0.34	<0.001	0.94, 0.44	<0.001	0.95, 0.45	<0.001	0.92, 0.43	<0.001	0.82, 0.39	<0.001	0.64, 0.30	<0.001
	Shinbashi Station	Tokyo	0.86, 0.39	<0.001	0.85, 0.39	<0.001	0.87, 0.46	<0.001	0.87, 0.44	<0.001	0.84, 0.46	<0.001	0.74, 0.39	<0.001
	Shinagawa Station	Tokyo	0.81, 0.42	<0.001	0.84, 0.40	<0.001	0.79, 0.39	<0.001	0.82, 0.39	<0.001	0.73, 0.39	<0.001	0.60, 0.33	<0.001
	Ueno Station	Tokyo	0.89, 0.48	<0.001	0.87, 0.58	<0.001	0.87, 0.61	<0.001	0.86, 0.61	<0.001	0.75, 0.50	<0.001	0.59, 0.38	<0.001
	Ginza Station	Tokyo	1.04, 0.45	<0.001	0.93, 0.42	<0.001	0.95, 0.48	<0.001	0.90, 0.47	<0.001	0.82, 0.42	<0.001	0.65, 0.31	<0.001
	Roppongi Station	Tokyo	0.88, 0.50	<0.001	0.96, 0.53	<0.001	0.90, 0.60	<0.001	0.99, 0.64	<0.001	0.94, 0.59	<0.001	0.88, 0.46	<0.001
	Kichijoji Station	Tokyo	0.88, 0.54	<0.001	0.95, 0.57	<0.001	0.98, 0.66	<0.001	0.97, 0.67	<0.001	0.91, 0.66	<0.001	0.78, 0.55	<0.001

	Motomachi-Chukagai Station	Kanagawa	0.99, 0.84	<0.001	0.96, 0.75	<0.001	1.02, 0.81	<0.001	1.00, 0.85	<0.001	0.95, 0.81	<0.001	0.82, 0.70	<0.001
	Sakuragicho Station	Kanagawa	1.14, 0.68	<0.001	0.91, 0.66	<0.001	0.94, 0.73	<0.001	0.98, 0.72	<0.001	0.85, 0.65	<0.001	0.77, 0.47	<0.001
	Kannai Station	Kanagawa	1.28, 0.74	<0.001	1.08, 0.69	<0.001	1.02, 0.73	<0.001	1.12, 0.87	<0.001	1.06, 0.80	<0.001	1.06, 0.71	<0.001
	Hamamatsu Station	Shizuoka	1.03, 0.57	<0.001	0.84, 0.59	<0.001	0.90, 0.65	<0.001	0.91, 0.61	<0.001	0.86, 0.59	<0.001	0.77, 0.47	<0.001
	Sakae Station	Aichi	0.89, 0.44	<0.001	0.93, 0.53	<0.001	0.94, 0.58	<0.001	0.99, 0.61	<0.001	0.94, 0.58	<0.001	0.67, 0.43	<0.001
	Fushimi Station	Aichi	0.93, 0.60	<0.001	1.00, 0.56	<0.001	1.04, 0.70	<0.001	1.10, 0.71	<0.001	0.95, 0.60	<0.001	0.64, 0.42	<0.001
	Kawaramachi Station	Kyoto	0.95, 0.44	<0.001	0.93, 0.57	<0.001	0.95, 0.64	<0.001	0.98, 0.65	<0.001	0.84, 0.54	<0.001	0.66, 0.39	<0.001
	Shinsaibashi Station	Osaka	1.03, 0.52	<0.001	0.99, 0.57	<0.001	1.09, 0.69	<0.001	1.10, 0.67	<0.001	1.00, 0.64	<0.001	0.89, 0.59	<0.001
	Namba Station	Osaka	0.88, 0.37	<0.001	0.87, 0.46	<0.001	0.84, 0.48	<0.001	0.87, 0.52	<0.001	0.79, 0.46	<0.001	0.61, 0.34	<0.001
	Kitashinchi Station	Osaka	1.07, 0.53	<0.001	0.95, 0.51	<0.001	1.01, 0.57	<0.001	1.03, 0.60	<0.001	0.95, 0.56	<0.001	0.84, 0.41	<0.001
	Tamachi Station	Okayama	1.14, 0.84	<0.001	1.16, 0.87	<0.001	1.30, 0.95	<0.001	1.15, 0.89	<0.001	1.08, 0.76	<0.001	0.78, 0.69	<0.01
	Hatchobori Station	Hiroshima	0.97, 0.55	<0.001	0.96, 0.62	<0.001	1.03, 0.68	<0.001	1.10, 0.67	<0.001	0.94, 0.58	<0.001	0.84, 0.53	<0.001
	Kawaramachi Station	Kagawa	1.04, 0.72	<0.001	1.14, 0.84	<0.001	1.00, 0.77	<0.001	1.16, 0.90	<0.001	0.93, 0.77	<0.001	1.02, 0.89	<0.001
	Hakata Station	Fukuoka	0.90, 0.41	<0.001	0.83, 0.45	<0.001	0.85, 0.50	<0.001	0.86, 0.47	<0.001	0.86, 0.46	<0.001	0.69, 0.36	<0.001
	Nakasu-kawabata Station	Fukuoka	0.99, 0.63	<0.001	1.05, 0.61	<0.001	1.10, 0.66	<0.001	1.25, 0.74	<0.001	1.19, 0.63	<0.001	0.85, 0.55	<0.001
	Kanko-dori Station	Nagasaki	1.22, 0.84	<0.001	0.98, 0.71	<0.001	0.98, 0.78	<0.001	0.97, 0.81	<0.001	0.93, 0.77	<0.001	0.95, 0.78	<0.001
	Makishi Station	Okinawa	1.61, 1.11	<0.001	1.15, 0.89	<0.001	1.21, 0.93	<0.001	1.05, 0.84	<0.001	1.06, 0.95	<0.001	0.90, 0.69	<0.001
12:00−15:59 PM														
	Susukino Station	Hokkaido	1.10, 0.62	<0.001	0.91, 0.66	<0.001	0.92, 0.74	<0.001	0.94, 0.76	<0.001	0.86, 0.68	<0.001	0.69, 0.44	<0.001
	Odori Station	Hokkaido	0.93, 0.55	<0.001	1.04, 0.73	<0.001	0.94, 0.67	<0.001	0.94, 0.64	<0.001	0.87, 0.57	<0.001	0.60, 0.37	<0.001
	Central Hirosaki Station	Aomori	1.15, 1.07	<0.01	0.99, 0.82	<0.001	1.25, 0.87	<0.001	1.21, 0.93	<0.001	1.15, 0.93	<0.001	1.01, 0.92	<0.01
	Kotodai-Koen Station	Miyagi	0.98, 0.64	<0.001	1.02, 0.72	<0.001	1.01, 0.72	<0.001	1.04, 0.76	<0.001	0.93, 0.68	<0.001	0.63, 0.42	<0.001
	Tobu-Utsunomiya station	Tochigi	1.51, 1.18	<0.001	0.81, 0.58	<0.001	0.87, 0.55	<0.001	1.08, 0.71	<0.001	0.82, 0.60	<0.001	0.70, 0.34	<0.001
	Chuo Maebashi Station	Gunma	1.81, 1.79	0.68	0.47, 0.46	0.40	0.61, 0.57	<0.01	0.89, 0.80	<0.05	0.79, 0.77	0.50	0.92, 0.67	<0.001
	Shibuya Station	Tokyo	0.75, 0.26	<0.001	0.79, 0.41	<0.001	0.77, 0.46	<0.001	0.80, 0.49	<0.001	0.69, 0.42	<0.001	0.53, 0.29	<0.001
	Shibuya Center Street	Tokyo	0.70, 0.24	<0.001	0.72, 0.38	<0.001	0.70, 0.43	<0.001	0.73, 0.45	<0.001	0.61, 0.37	<0.001	0.47, 0.27	<0.001
	Harajuku Station	Tokyo	0.85, 0.38	<0.001	0.75, 0.52	<0.001	0.70, 0.51	<0.001	0.76, 0.60	<0.001	0.68, 0.54	<0.001	0.56, 0.47	<0.001
	Shinjuku Kabukicho	Tokyo	0.81, 0.30	<0.001	0.81, 0.45	<0.001	0.77, 0.45	<0.001	0.78, 0.46	<0.001	0.69, 0.38	<0.001	0.52, 0.27	<0.001
	Ikebukuro Station	Tokyo	1.03, 0.39	<0.001	0.95, 0.49	<0.001	0.97, 0.54	<0.001	1.00, 0.58	<0.001	0.92, 0.49	<0.001	0.75, 0.34	<0.001
	Tokyo Station	Tokyo	0.99, 0.32	<0.001	0.94, 0.41	<0.001	0.97, 0.42	<0.001	0.96, 0.42	<0.001	0.77, 0.36	<0.001	0.59, 0.22	<0.001
	Shinbashi Station	Tokyo	0.90, 0.35	<0.001	0.82, 0.36	<0.001	0.84, 0.42	<0.001	0.86, 0.40	<0.001	0.80, 0.41	<0.001	0.67, 0.30	<0.001

	Shinagawa Station	Tokyo	0.87, 0.43	<0.001	0.83, 0.41	<0.001	0.82, 0.41	<0.001	0.85, 0.40	<0.001	0.70, 0.38	<0.001	0.56, 0.29	<0.001
	Ueno Station	Tokyo	0.85, 0.42	<0.001	0.88, 0.60	<0.001	0.84, 0.58	<0.001	0.84, 0.59	<0.001	0.67, 0.46	<0.001	0.52, 0.32	<0.001
	Ginza Station	Tokyo	0.97, 0.27	<0.001	0.91, 0.32	<0.001	0.92, 0.39	<0.001	0.87, 0.36	<0.001	0.71, 0.28	<0.001	0.58, 0.21	<0.001
	Roppongi Station	Tokyo	0.83, 0.46	<0.001	0.95, 0.52	<0.001	0.93, 0.58	<0.001	0.93, 0.57	<0.001	0.83, 0.51	<0.001	0.81, 0.44	<0.001
	Kichijoji Station	Tokyo	0.91, 0.49	<0.001	0.89, 0.53	<0.001	0.86, 0.56	<0.001	0.87, 0.61	<0.001	0.85, 0.59	<0.001	0.74, 0.43	<0.001
	Motomachi-Chukagai Station	Kanagawa	0.97, 0.85	<0.001	0.85, 0.72	<0.001	1.04, 0.94	<0.001	0.96, 0.88	<0.001	0.85, 0.78	<0.001	0.80, 0.68	<0.001
	Sakuragicho Station	Kanagawa	1.05, 0.56	<0.001	0.88, 0.64	<0.001	0.85, 0.70	<0.001	0.87, 0.69	<0.001	0.77, 0.55	<0.001	0.63, 0.39	<0.001
	Kannai Station	Kanagawa	1.22, 0.86	<0.001	1.05, 0.70	<0.001	0.98, 0.70	<0.001	1.07, 0.86	<0.001	1.07, 0.80	<0.001	0.89, 0.59	<0.001
	Hamamatsu Station	Shizuoka	1.00, 0.50	<0.001	0.76, 0.53	<0.001	0.85, 0.61	<0.001	0.82, 0.59	<0.001	0.79, 0.54	<0.001	0.58, 0.43	<0.001
	Sakae Station	Aichi	0.92, 0.43	<0.001	0.88, 0.55	<0.001	0.93, 0.61	<0.001	1.00, 0.67	<0.001	0.78, 0.52	<0.001	0.55, 0.37	<0.001
	Fushimi Station	Aichi	0.99, 0.62	<0.001	0.96, 0.57	<0.001	1.05, 0.74	<0.001	1.06, 0.72	<0.001	0.81, 0.56	<0.001	0.66, 0.41	<0.001
	Kawaramachi Station	Kyoto	0.92, 0.35	<0.001	0.80, 0.47	<0.001	0.81, 0.50	<0.001	0.82, 0.53	<0.001	0.72, 0.39	<0.001	0.52, 0.34	<0.001
	Shinsaibashi Station	Osaka	0.96, 0.43	<0.001	0.95, 0.52	<0.001	1.03, 0.65	<0.001	0.98, 0.61	<0.001	0.86, 0.55	<0.001	0.71, 0.43	<0.001
	Namba Station	Osaka	0.86, 0.30	<0.001	0.75, 0.37	<0.001	0.71, 0.38	<0.001	0.71, 0.38	<0.001	0.63, 0.32	<0.001	0.54, 0.27	<0.001
	Kitashinchi Station	Osaka	1.13, 0.45	<0.001	0.95, 0.52	<0.001	0.94, 0.54	<0.001	0.97, 0.56	<0.001	0.88, 0.49	<0.001	0.70, 0.33	<0.001
	Tamachi Station	Okayama	1.02, 0.81	<0.001	1.19, 0.93	<0.001	1.18, 0.91	<0.001	1.12, 0.87	<0.001	1.05, 0.73	<0.001	0.68, 0.56	<0.001
	Hatchobori Station	Hiroshima	0.92, 0.51	<0.001	0.82, 0.55	<0.001	0.89, 0.63	<0.001	0.92, 0.64	<0.001	0.79, 0.52	<0.001	0.70, 0.43	<0.001
	Kawaramachi Station	Kagawa	0.84, 0.59	<0.001	1.05, 0.75	<0.001	1.01, 0.88	<0.01	1.05, 0.94	<0.01	0.84, 0.84	0.46	0.79, 0.72	<0.05
	Hakata Station	Fukuoka	0.90, 0.36	<0.001	0.82, 0.37	<0.001	0.81, 0.45	<0.001	0.79, 0.38	<0.001	0.77, 0.38	<0.001	0.63, 0.29	<0.001
	Nakasu-kawabata Station	Fukuoka	0.94, 0.58	<0.001	0.94, 0.63	<0.001	1.09, 0.73	<0.001	1.22, 0.78	<0.001	1.11, 0.62	<0.001	0.76, 0.43	<0.001
	Kanko-dori Station	Nagasaki	0.96, 0.71	<0.001	0.87, 0.67	<0.001	0.89, 0.72	<0.001	0.91, 0.75	<0.001	0.84, 0.68	<0.001	0.84, 0.61	<0.001
	Makishi Station	Okinawa	1.29, 1.04	<0.001	1.18, 0.91	<0.001	1.11, 0.87	<0.001	1.06, 0.94	<0.001	0.99, 0.91	<0.001	1.03, 0.85	<0.001
16:00−19:59 PM														
	Susukino Station	Hokkaido	0.95, 0.47	<0.001	0.77, 0.49	<0.001	0.79, 0.55	<0.001	0.82, 0.54	<0.001	0.72, 0.44	<0.001	0.54, 0.35	<0.001
	Odori Station	Hokkaido	0.90, 0.53	<0.001	1.01, 0.64	<0.001	0.93, 0.61	<0.001	0.94, 0.57	<0.001	0.86, 0.50	<0.001	0.63, 0.41	<0.001
	Central Hirosaki Station	Aomori	0.78, 0.48	<0.001	1.13, 0.81	<0.001	1.15, 0.73	<0.001	1.02, 0.75	<0.001	1.41, 1.06	<0.001	0.93, 0.94	0.79
	Kotodai-Koen Station	Miyagi	0.93, 0.60	<0.001	0.99, 0.67	<0.001	0.96, 0.66	<0.001	0.99, 0.71	<0.001	0.82, 0.59	<0.001	0.72, 0.43	<0.001
	Tobu-Utsunomiya station	Tochigi	1.20, 0.84	<0.001	0.94, 0.61	<0.001	0.97, 0.52	<0.001	1.25, 0.68	<0.001	0.93, 0.63	<0.001	0.85, 0.49	<0.001
	Chuo Maebashi Station	Gunma	1.61, 1.39	0.10	0.50, 0.34	<0.001	0.68, 0.51	<0.001	0.87, 0.62	<0.001	0.62, 0.48	<0.001	1.21, 0.92	<0.001
	Shibuya Station	Tokyo	0.80, 0.24	<0.001	0.81, 0.42	<0.001	0.81, 0.46	<0.001	0.85, 0.52	<0.001	0.78, 0.45	<0.001	0.58, 0.32	<0.001
	Shibuya Center Street	Tokyo	0.74, 0.22	<0.001	0.73, 0.38	<0.001	0.74, 0.42	<0.001	0.77, 0.48	<0.001	0.69, 0.40	<0.001	0.51, 0.29	<0.001

	Harajuku Station	Tokyo	0.89, 0.34	<0.001	0.96, 0.58	<0.001	0.90, 0.60	<0.001	0.89, 0.68	<0.001	0.93, 0.65	<0.001	0.74, 0.47	<0.001
	Shinjuku Kabukicho	Tokyo	0.83, 0.27	<0.001	0.86, 0.42	<0.001	0.85, 0.45	<0.001	0.86, 0.48	<0.001	0.77, 0.42	<0.001	0.55, 0.25	<0.001
	Ikebukuro Station	Tokyo	1.08, 0.44	<0.001	1.05, 0.53	<0.001	1.06, 0.59	<0.001	1.07, 0.63	<0.001	1.02, 0.55	<0.001	0.82, 0.38	<0.001
	Tokyo Station	Tokyo	0.92, 0.30	<0.001	0.92, 0.40	<0.001	0.91, 0.43	<0.001	0.93, 0.44	<0.001	0.83, 0.38	<0.001	0.62, 0.22	<0.001
	Shinbashi Station	Tokyo	0.91, 0.34	<0.001	0.89, 0.37	<0.001	0.88, 0.43	<0.001	0.89, 0.43	<0.001	0.84, 0.41	<0.001	0.62, 0.24	<0.001
	Shinagawa Station	Tokyo	0.83, 0.42	<0.001	0.82, 0.38	<0.001	0.83, 0.42	<0.001	0.84, 0.42	<0.001	0.78, 0.41	<0.001	0.59, 0.33	<0.001
	Ueno Station	Tokyo	0.81, 0.43	<0.001	0.87, 0.57	<0.001	0.88, 0.61	<0.001	0.90, 0.62	<0.001	0.81, 0.51	<0.001	0.60, 0.32	<0.001
	Ginza Station	Tokyo	0.93, 0.27	<0.001	0.96, 0.31	<0.001	0.94, 0.37	<0.001	0.88, 0.36	<0.001	0.78, 0.29	<0.001	0.57, 0.20	<0.001
	Roppongi Station	Tokyo	0.97, 0.44	<0.001	1.02, 0.50	<0.001	0.95, 0.57	<0.001	0.99, 0.58	<0.001	0.95, 0.53	<0.001	0.70, 0.37	<0.001
	Kichijoji Station	Tokyo	0.97, 0.46	<0.001	0.98, 0.58	<0.001	0.91, 0.60	<0.001	0.92, 0.65	<0.001	0.89, 0.59	<0.001	0.69, 0.43	<0.001
	Motomachi-Chukagai Station	Kanagawa	1.04, 0.89	<0.001	0.92, 0.74	<0.001	1.00, 0.83	<0.001	0.97, 0.83	<0.001	0.92, 0.79	<0.001	0.80, 0.66	<0.001
	Sakuragicho Station	Kanagawa	0.97, 0.50	<0.001	0.90, 0.62	<0.001	0.88, 0.68	<0.001	0.90, 0.67	<0.001	0.81, 0.59	<0.001	0.64, 0.39	<0.001
	Kannai Station	Kanagawa	1.22, 0.81	<0.001	1.06, 0.74	<0.001	1.05, 0.76	<0.001	1.13, 0.88	<0.001	1.09, 0.78	<0.001	0.85, 0.51	<0.001
	Hamamatsu Station	Shizuoka	0.94, 0.46	<0.001	0.85, 0.60	<0.001	0.89, 0.64	<0.001	0.88, 0.61	<0.001	0.80, 0.57	<0.001	0.72, 0.52	<0.001
	Sakae Station	Aichi	0.83, 0.38	<0.001	0.91, 0.56	<0.001	0.89, 0.55	<0.001	0.96, 0.58	<0.001	0.88, 0.48	<0.001	0.62, 0.35	<0.001
	Fushimi Station	Aichi	1.01, 0.60	<0.001	1.02, 0.58	<0.001	1.10, 0.70	<0.001	1.06, 0.69	<0.001	0.89, 0.54	<0.001	0.63, 0.31	<0.001
	Kawaramachi Station	Kyoto	0.82, 0.29	<0.001	0.77, 0.44	<0.001	0.91, 0.51	<0.001	0.90, 0.51	<0.001	0.78, 0.40	<0.001	0.62, 0.30	<0.001
	Shinsaibashi Station	Osaka	0.95, 0.43	<0.001	0.97, 0.49	<0.001	1.04, 0.61	<0.001	1.04, 0.62	<0.001	0.95, 0.56	<0.001	0.71, 0.37	<0.001
	Namba Station	Osaka	0.88, 0.27	<0.001	0.85, 0.40	<0.001	0.80, 0.43	<0.001	0.82, 0.44	<0.001	0.71, 0.37	<0.001	0.58, 0.25	<0.001
	Kitashinchi Station	Osaka	1.07, 0.38	<0.001	0.94, 0.46	<0.001	0.98, 0.50	<0.001	1.02, 0.52	<0.001	0.91, 0.44	<0.001	0.75, 0.30	<0.001
	Tamachi Station	Okayama	0.98, 0.70	<0.001	1.15, 0.80	<0.001	1.19, 0.82	<0.001	1.11, 0.79	<0.001	1.02, 0.71	<0.001	0.71, 0.56	<0.001
	Hatchobori Station	Hiroshima	0.81, 0.39	<0.001	0.77, 0.44	<0.001	0.86, 0.52	<0.001	0.89, 0.52	<0.001	0.78, 0.45	<0.001	0.64, 0.33	<0.001
	Kawaramachi Station	Kagawa	1.16, 0.61	<0.001	1.05, 0.71	<0.001	1.05, 0.80	<0.001	1.04, 0.94	<0.001	0.86, 0.76	<0.01	0.87, 0.71	<0.001
	Hakata Station	Fukuoka	0.86, 0.31	<0.001	0.87, 0.38	<0.001	0.86, 0.42	<0.001	0.86, 0.41	<0.001	0.85, 0.42	<0.001	0.66, 0.27	<0.001
	Nakasu-kawabata Station	Fukuoka	0.89, 0.50	<0.001	0.99, 0.59	<0.001	1.01, 0.57	<0.001	1.12, 0.61	<0.001	0.91, 0.50	<0.001	0.71, 0.42	<0.001
	Kanko-dori Station	Nagasaki	0.94, 0.59	<0.001	0.91, 0.67	<0.001	0.93, 0.72	<0.001	0.95, 0.75	<0.001	0.85, 0.66	<0.001	0.78, 0.52	<0.001
	Makishi Station	Okinawa	1.62, 1.17	<0.001	1.13, 0.84	<0.001	1.17, 0.89	<0.001	0.99, 0.85	<0.001	1.07, 0.92	<0.001	1.12, 0.80	<0.001
20:00−23:59 PM														
	Susukino Station	Hokkaido	0.84, 0.27	<0.001	0.56, 0.22	<0.001	0.57, 0.25	<0.001	0.56, 0.24	<0.001	0.46, 0.17	<0.001	0.43, 0.22	<0.001
	Odori Station	Hokkaido	0.80, 0.36	<0.001	0.86, 0.50	<0.001	0.82, 0.47	<0.001	0.81, 0.43	<0.001	0.62, 0.32	<0.001	0.56, 0.35	<0.001
	Central Hirosaki Station	Aomori	0.55, 0.15	<0.001	1.17, 0.60	<0.001	0.98, 0.47	<0.001	0.83, 0.46	<0.001	0.79, 0.46	<0.001	0.78, 0.72	<0.001

Kotodai-Koen Station	Miyagi	0.77, 0.37	<0.001	0.76, 0.45	<0.001	0.92, 0.59	<0.001	0.92, 0.55	<0.001	0.68, 0.37	<0.001	0.91, 0.45	<0.001
Tobu-Utsunomiya station	Tochigi	1.24, 0.67	<0.001	1.02, 0.46	<0.001	0.96, 0.41	<0.001	1.25, 0.55	<0.001	0.87, 0.52	<0.001	0.87, 0.47	<0.001
Chuo Maebashi Station	Gunma	1.14, 1.27	<0.05	0.64, 0.37	<0.001	0.68, 0.42	<0.001	0.87, 0.48	<0.001	0.65, 0.39	<0.001	1.19, 0.64	<0.001
Shibuya Station	Tokyo	0.71, 0.16	<0.001	0.79, 0.33	<0.001	0.78, 0.35	<0.001	0.78, 0.36	<0.001	0.65, 0.27	<0.001	0.56, 0.24	<0.001
Shibuya Center Street	Tokyo	0.65, 0.15	<0.001	0.71, 0.28	<0.001	0.71, 0.31	<0.001	0.71, 0.32	<0.001	0.59, 0.24	<0.001	0.50, 0.22	<0.001
Harajuku Station	Tokyo	0.81, 0.24	<0.001	0.93, 0.41	<0.001	0.92, 0.50	<0.001	0.86, 0.45	<0.001	0.87, 0.41	<0.001	0.79, 0.35	<0.001
Shinjuku Kabukicho	Tokyo	0.86, 0.16	<0.001	0.85, 0.30	<0.001	0.84, 0.33	<0.001	0.82, 0.34	<0.001	0.74, 0.27	<0.001	0.64, 0.24	<0.001
Ikebukuro Station	Tokyo	1.02, 0.28	<0.001	1.01, 0.42	<0.001	0.98, 0.45	<0.001	0.98, 0.44	<0.001	0.87, 0.34	<0.001	0.66, 0.25	<0.001
Tokyo Station	Tokyo	0.79, 0.23	<0.001	0.90, 0.34	<0.001	0.90, 0.37	<0.001	0.86, 0.34	<0.001	0.71, 0.25	<0.001	0.57, 0.17	<0.001
Shinbashi Station	Tokyo	0.86, 0.22	<0.001	0.83, 0.26	<0.001	0.86, 0.32	<0.001	0.83, 0.28	<0.001	0.71, 0.22	<0.001	0.60, 0.16	<0.001
Shinagawa Station	Tokyo	0.77, 0.26	<0.001	0.77, 0.30	<0.001	0.81, 0.35	<0.001	0.78, 0.32	<0.001	0.64, 0.29	<0.001	0.53, 0.23	<0.001
Ueno Station	Tokyo	0.74, 0.37	<0.001	0.81, 0.41	<0.001	0.84, 0.45	<0.001	0.81, 0.43	<0.001	0.68, 0.28	<0.001	0.58, 0.27	<0.001
Ginza Station	Tokyo	0.92, 0.20	<0.001	0.85, 0.22	<0.001	0.76, 0.27	<0.001	0.78, 0.24	<0.001	0.63, 0.17	<0.001	0.53, 0.12	<0.001
Roppongi Station	Tokyo	0.94, 0.32	<0.001	0.98, 0.37	<0.001	0.88, 0.44	<0.001	0.90, 0.41	<0.001	0.85, 0.34	<0.001	0.71, 0.27	<0.001
Kichijoji Station	Tokyo	0.88, 0.32	<0.001	0.91, 0.39	<0.001	0.89, 0.48	<0.001	0.84, 0.43	<0.001	0.78, 0.39	<0.001	0.65, 0.39	<0.001
Motomachi-Chukagai Station	Kanagawa	0.95, 0.62	<0.001	0.95, 0.67	<0.001	0.91, 0.61	<0.001	0.91, 0.66	<0.001	1.00, 0.75	<0.001	0.78, 0.62	<0.001
Sakuragicho Station	Kanagawa	0.89, 0.39	<0.001	0.91, 0.48	<0.001	0.91, 0.54	<0.001	0.85, 0.45	<0.001	0.73, 0.40	<0.001	0.51, 0.28	<0.001
Kannai Station	Kanagawa	1.20, 0.54	<0.001	1.02, 0.63	<0.001	1.17, 0.59	<0.001	1.00, 0.59	<0.001	0.91, 0.43	<0.001	0.78, 0.36	<0.001
Hamamatsu Station	Shizuoka	0.66, 0.28	<0.001	0.86, 0.52	<0.001	0.83, 0.50	<0.001	0.83, 0.43	<0.001	0.68, 0.44	<0.001	0.93, 0.65	<0.001
Sakae Station	Aichi	0.66, 0.24	<0.001	0.85, 0.32	<0.001	0.82, 0.38	<0.001	0.83, 0.36	<0.001	0.71, 0.32	<0.001	0.48, 0.20	<0.001
Fushimi Station	Aichi	0.85, 0.38	<0.001	0.87, 0.44	<0.001	0.89, 0.47	<0.001	0.85, 0.50	<0.001	0.80, 0.39	<0.001	0.54, 0.19	<0.001
Kawaramachi Station	Kyoto	0.92, 0.20	<0.001	0.67, 0.31	<0.001	0.76, 0.35	<0.001	0.87, 0.38	<0.001	0.77, 0.29	<0.001	0.68, 0.21	<0.001
Shinsaibashi Station	Osaka	0.99, 0.33	<0.001	0.90, 0.34	<0.001	0.87, 0.39	<0.001	0.87, 0.37	<0.001	0.74, 0.30	<0.001	0.63, 0.29	<0.001
Namba Station	Osaka	0.84, 0.17	<0.001	0.86, 0.27	<0.001	0.83, 0.30	<0.001	0.80, 0.28	<0.001	0.65, 0.24	<0.001	0.61, 0.16	<0.001
Kitashinchi Station	Osaka	0.99, 0.20	<0.001	0.90, 0.24	<0.001	0.93, 0.27	<0.001	0.90, 0.26	<0.001	0.79, 0.20	<0.001	0.63, 0.16	<0.001
Tamachi Station	Okayama	0.91, 0.52	<0.001	0.98, 0.50	<0.001	1.01, 0.59	<0.001	0.88, 0.51	<0.001	0.80, 0.46	<0.001	0.70, 0.52	<0.001
Hatchobori Station	Hiroshima	0.71, 0.25	<0.001	0.60, 0.24	<0.001	0.74, 0.27	<0.001	0.72, 0.27	<0.001	0.66, 0.22	<0.001	0.54, 0.18	<0.001
Kawaramachi Station	Kagawa	1.02, 0.36	<0.001	1.35, 0.68	<0.001	1.20, 0.80	<0.001	1.21, 0.87	<0.001	0.91, 0.69	<0.01	1.05, 0.86	<0.001
Hakata Station	Fukuoka	0.79, 0.18	<0.001	0.81, 0.25	<0.001	0.83, 0.30	<0.001	0.71, 0.24	<0.001	0.67, 0.27	<0.001	0.52, 0.22	<0.001
Nakasu-kawabata Station	Fukuoka	0.75, 0.33	<0.001	0.74, 0.27	<0.001	0.74, 0.29	<0.001	0.77, 0.30	<0.001	0.69, 0.26	<0.001	0.69, 0.31	<0.001
Kanko-dori Station	Nagasaki	0.98, 0.46	<0.001	0.86, 0.39	<0.001	0.81, 0.47	<0.001	0.86, 0.52	<0.001	0.72, 0.50	<0.001	0.62, 0.26	<0.001

		Makishi Station	Okinawa	1.54, 0.96	<0.001	0.88, 0.62	<0.001	1.10, 0.87	<0.001	1.01, 0.69	<0.001	1.12, 0.78	<0.001	1.13, 0.71	<0.001
Second															
	0:00–7:59 AM														
		Susukino Station	Hokkaido	–	–	–	–	–	–	–	–	–	–	–	–
		Odori Station	Hokkaido	–	–	–	–	–	–	–	–	–	–	–	–
		Central Hirosaki Station	Aomori	–	–	–	–	–	–	–	–	–	–	–	–
		Kotodai-Koen Station	Miyagi	–	–	–	–	–	–	–	–	–	–	–	–
		Tobu-Utsunomiya station	Tochigi	0.37, 0.25	<0.001	0.42, 0.39	<0.05	0.53, 0.57	0.80	0.69, 0.61	<0.05	0.55, 0.56	0.96	0.48, 0.40	<0.001
		Chuo Maebashi Station	Gunma	–	–	–	–	–	–	–	–	–	–	–	–
		Shibuya Station	Tokyo	0.66, 0.44	<0.001	0.31, 0.29	<0.001	0.37, 0.36	0.05	0.38, 0.36	<0.01	0.36, 0.38	<0.001	0.55, 0.52	<0.001
		Shibuya Center Street	Tokyo	0.63, 0.41	<0.001	0.29, 0.26	<0.001	0.34, 0.32	<0.001	0.34, 0.32	<0.001	0.32, 0.34	<0.05	0.51, 0.47	<0.001
		Harajuku Station	Tokyo	0.78, 0.63	<0.001	0.47, 0.44	<0.001	0.55, 0.55	0.05	0.51, 0.48	<0.01	0.47, 0.48	<0.01	0.84, 0.79	0.89
		Shinjuku Kabukicho	Tokyo	0.62, 0.47	<0.001	0.45, 0.39	<0.001	0.44, 0.40	<0.001	0.47, 0.46	<0.001	0.41, 0.41	0.94	0.63, 0.56	<0.001
		Ikebukuro Station	Tokyo	0.67, 0.53	<0.001	0.45, 0.40	<0.001	0.48, 0.44	<0.001	0.51, 0.47	<0.001	0.41, 0.41	0.46	0.60, 0.49	<0.001
		Tokyo Station	Tokyo	0.58, 0.50	<0.001	0.50, 0.48	<0.05	0.52, 0.47	<0.001	0.54, 0.50	<0.001	0.44, 0.43	<0.001	0.51, 0.42	<0.001
		Shinbashi Station	Tokyo	0.64, 0.48	<0.001	0.51, 0.46	<0.001	0.48, 0.43	<0.001	0.51, 0.48	<0.001	0.43, 0.43	<0.05	0.51, 0.51	0.30
		Shinagawa Station	Tokyo	0.62, 0.52	<0.001	0.59, 0.50	<0.001	0.59, 0.53	<0.001	0.63, 0.59	<0.001	0.50, 0.53	<0.001	0.43, 0.42	<0.05
		Ueno Station	Tokyo	0.83, 0.71	<0.001	0.61, 0.51	<0.001	0.63, 0.58	<0.001	0.60, 0.56	<0.001	0.46, 0.44	<0.001	0.43, 0.41	<0.01
		Ginza Station	Tokyo	0.63, 0.50	<0.001	0.58, 0.52	<0.001	0.57, 0.51	<0.001	0.61, 0.58	<0.001	0.45, 0.46	0.66	0.46, 0.40	<0.001
		Roppongi Station	Tokyo	0.57, 0.45	<0.001	0.55, 0.52	<0.001	0.50, 0.47	<0.001	0.51, 0.47	<0.001	0.47, 0.46	0.30	0.65, 0.67	0.19
		Kichijoji Station	Tokyo	0.79, 0.64	<0.001	0.50, 0.44	<0.001	0.69, 0.67	<0.001	0.63, 0.56	<0.001	0.54, 0.53	0.95	0.63, 0.62	0.99
		Motomachi-Chukagai Station	Kanagawa	0.56, 0.51	<0.001	0.55, 0.51	<0.001	0.57, 0.51	<0.001	0.69, 0.75	<0.001	0.69, 0.68	0.07	0.60, 0.54	<0.001
		Sakuragicho Station	Kanagawa	0.68, 0.56	<0.001	0.66, 0.56	<0.001	0.63, 0.61	<0.001	0.67, 0.64	<0.001	0.61, 0.60	0.30	0.59, 0.58	0.39
		Kannai Station	Kanagawa	0.93, 0.81	<0.001	0.65, 0.62	<0.001	0.72, 0.63	<0.001	0.58, 0.60	<0.001	0.64, 0.60	<0.01	1.24, 1.04	<0.001
		Hamamatsu Station	Shizuoka	–	–	–	–	–	–	–	–	–	–	–	–
		Sakae Station	Aichi	0.55, 0.51	<0.001	0.44, 0.40	<0.01	0.44, 0.40	<0.05	0.42, 0.37	<0.001	0.34, 0.35	0.11	0.37, 0.35	0.07
		Fushimi Station	Aichi	0.61, 0.58	<0.01	0.59, 0.52	<0.01	0.59, 0.50	<0.01	0.60, 0.54	<0.05	0.49, 0.49	0.47	0.44, 0.39	<0.05
		Kawaramachi Station	Kyoto	0.61, 0.38	<0.001	0.29, 0.22	<0.001	0.41, 0.32	<0.001	0.42, 0.37	<0.001	0.37, 0.37	0.77	0.65, 0.68	0.12
		Shinsaibashi Station	Osaka	0.52, 0.49	<0.001	0.38, 0.37	<0.05	0.47, 0.44	0.12	0.49, 0.49	0.84	0.43, 0.44	<0.05	0.50, 0.49	0.90
		Namba Station	Osaka	0.56, 0.47	<0.001	0.33, 0.30	<0.001	0.42, 0.39	<0.001	0.42, 0.44	0.65	0.35, 0.36	<0.05	0.50, 0.52	<0.05
		Kitashinchi Station	Osaka	0.48, 0.36	<0.001	0.39, 0.34	<0.001	0.46, 0.39	<0.01	0.50, 0.44	<0.001	0.39, 0.38	<0.05	0.44, 0.43	0.06

	Tamachi Station	Okayama	–	–	–	–	–	–	–	–	–	–	–	–
	Hatchobori Station	Hiroshima	–	–	–	–	–	–	–	–	–	–	–	–
	Kawaramachi Station	Kagawa	–	–	–	–	–	–	–	–	–	–	–	–
	Hakata Station	Fukuoka	0.55, 0.41	<0.001	0.54, 0.43	<0.001	0.66, 0.56	<0.001	0.58, 0.46	<0.001	0.49, 0.41	<0.001	0.58, 0.42	<0.001
	Nakasu-kawabata Station	Fukuoka	0.52, 0.31	<0.001	0.54, 0.42	<0.001	0.67, 0.51	<0.001	0.66, 0.53	<0.001	0.59, 0.53	<0.001	0.68, 0.56	<0.001
	Kanko-dori Station	Nagasaki	–	–	–	–	–	–	–	–	–	–	–	–
	Makishi Station	Okinawa	–	–	–	–	–	–	–	–	–	–	–	–
08:00–11:59 AM														
	Susukino Station	Hokkaido	–	–	–	–	–	–	–	–	–	–	–	–
	Odori Station	Hokkaido	–	–	–	–	–	–	–	–	–	–	–	–
	Central Hirosaki Station	Aomori	–	–	–	–	–	–	–	–	–	–	–	–
	Kotodai-Koen Station	Miyagi	–	–	–	–	–	–	–	–	–	–	–	–
	Tobu-Utsunomiya station	Tochigi	0.89, 0.88	0.90	0.87, 0.92	0.25	0.81, 0.83	0.49	0.90, 0.88	0.35	0.80, 0.76	0.14	0.55, 0.51	0.23
	Chuo Maebashi Station	Gunma	–	–	–	–	–	–	–	–	–	–	–	–
	Shibuya Station	Tokyo	0.93, 0.96	<0.001	0.64, 0.61	<0.01	0.61, 0.57	<0.001	0.59, 0.56	<0.001	0.47, 0.46	0.23	0.55, 0.50	<0.001
	Shibuya Center Street	Tokyo	0.88, 0.91	<0.001	0.59, 0.57	<0.05	0.56, 0.52	<0.001	0.54, 0.51	<0.001	0.42, 0.42	0.64	0.49, 0.45	<0.001
	Harajuku Station	Tokyo	0.99, 1.01	<0.001	0.81, 0.76	<0.001	0.73, 0.69	<0.001	0.68, 0.64	<0.001	0.51, 0.51	0.11	0.72, 0.66	<0.001
	Shinjuku Kabukicho	Tokyo	0.87, 0.92	<0.001	0.64, 0.63	0.64	0.63, 0.61	<0.01	0.61, 0.59	0.13	0.47, 0.47	0.67	0.53, 0.49	<0.001
	Ikebukuro Station	Tokyo	0.97, 1.00	<0.001	0.65, 0.64	<0.001	0.67, 0.63	<0.001	0.65, 0.62	<0.001	0.51, 0.49	<0.001	0.60, 0.52	<0.001
	Tokyo Station	Tokyo	1.07, 0.95	<0.001	0.85, 0.77	<0.001	0.80, 0.72	<0.001	0.76, 0.70	<0.001	0.52, 0.51	<0.001	0.57, 0.46	<0.001
	Shinbashi Station	Tokyo	1.02, 0.91	<0.001	0.76, 0.68	<0.001	0.72, 0.65	<0.001	0.71, 0.66	<0.001	0.55, 0.54	<0.01	0.63, 0.60	<0.001
	Shinagawa Station	Tokyo	0.92, 0.80	<0.001	0.74, 0.65	<0.001	0.66, 0.59	<0.001	0.65, 0.59	<0.001	0.50, 0.49	<0.05	0.52, 0.49	<0.001
	Ueno Station	Tokyo	0.93, 0.87	<0.001	0.68, 0.61	<0.001	0.64, 0.59	<0.001	0.62, 0.56	<0.001	0.46, 0.43	<0.001	0.43, 0.36	<0.001
	Ginza Station	Tokyo	1.42, 1.43	<0.01	0.97, 0.95	0.30	0.96, 0.95	0.06	0.91, 0.87	<0.001	0.68, 0.69	0.93	0.64, 0.59	<0.001
	Roppongi Station	Tokyo	0.97, 0.98	0.34	0.85, 0.84	0.98	0.78, 0.80	<0.01	0.86, 0.88	0.05	0.64, 0.70	<0.001	0.76, 0.77	0.81
	Kichijoji Station	Tokyo	1.19, 1.23	<0.001	0.77, 0.74	<0.001	0.82, 0.79	<0.001	0.76, 0.72	<0.001	0.65, 0.61	<0.001	0.69, 0.62	<0.001
	Motomachi-Chukagai Station	Kanagawa	0.94, 0.86	<0.001	0.82, 0.82	0.30	0.90, 0.86	<0.001	1.00, 1.01	0.98	0.76, 0.78	<0.01	0.73, 0.64	<0.001
	Sakuragicho Station	Kanagawa	1.37, 1.26	<0.001	1.01, 0.95	<0.001	0.95, 0.93	<0.001	0.99, 0.95	<0.001	0.83, 0.79	<0.01	0.73, 0.68	<0.001
	Kannai Station	Kanagawa	1.34, 1.27	<0.001	0.90, 0.89	0.54	0.96, 0.88	<0.001	0.97, 0.95	0.77	0.86, 0.85	0.32	0.94, 0.85	<0.001
	Hamamatsu Station	Shizuoka	–	–	–	–	–	–	–	–	–	–	–	–
	Sakae Station	Aichi	0.94, 0.90	<0.01	0.81, 0.77	<0.01	0.79, 0.75	<0.05	0.75, 0.71	<0.01	0.56, 0.56	0.81	0.54, 0.50	0.12

	Fushimi Station	Aichi	1.09, 1.05	<0.05	0.98, 0.89	<0.001	0.92, 0.87	<0.01	0.92, 0.89	<0.01	0.67, 0.66	0.88	0.68, 0.61	<0.05
	Kawaramachi Station	Kyoto	1.06, 1.05	0.21	0.65, 0.65	0.95	0.63, 0.64	0.44	0.64, 0.63	0.19	0.52, 0.50	<0.05	0.48, 0.48	0.76
	Shinsaibashi Station	Osaka	1.33, 1.33	0.71	1.08, 1.05	<0.05	1.12, 1.08	<0.05	1.06, 1.07	0.42	0.76, 0.79	<0.01	0.86, 0.88	0.72
	Namba Station	Osaka	0.89, 0.87	<0.01	0.69, 0.67	<0.01	0.65, 0.63	<0.05	0.65, 0.64	0.19	0.46, 0.47	0.19	0.49, 0.48	<0.05
	Kitashinchi Station	Osaka	1.39, 1.30	<0.01	0.92, 0.89	<0.01	0.91, 0.89	<0.01	0.87, 0.86	0.16	0.70, 0.71	0.10	0.80, 0.76	<0.01
	Tamachi Station	Okayama	–	–	–	–	–	–	–	–	–	–	–	–
	Hatchobori Station	Hiroshima	–	–	–	–	–	–	–	–	–	–	–	–
	Kawaramachi Station	Kagawa	–	–	–	–	–	–	–	–	–	–	–	–
	Hakata Station	Fukuoka	0.90, 0.78	<0.001	0.75, 0.69	<0.001	0.79, 0.71	<0.001	0.73, 0.64	<0.001	0.60, 0.53	<0.001	0.61, 0.50	<0.001
	Nakasu-kawabata Station	Fukuoka	1.07, 0.90	<0.001	1.11, 1.04	<0.01	1.20, 1.08	<0.001	1.20, 1.10	<0.001	0.92, 0.92	0.70	0.87, 0.79	<0.01
	Kanko-dori Station	Nagasaki	–	–	–	–	–	–	–	–	–	–	–	–
	Makishi Station	Okinawa	–	–	–	–	–	–	–	–	–	–	–	–
12:00−15:59 PM														
	Susukino Station	Hokkaido	–	–	–	–	–	–	–	–	–	–	–	–
	Odori Station	Hokkaido	–	–	–	–	–	–	–	–	–	–	–	–
	Central Hirosaki Station	Aomori	–	–	–	–	–	–	–	–	–	–	–	–
	Kotodai-Koen Station	Miyagi	–	–	–	–	–	–	–	–	–	–	–	–
	Tobu-Utsunomiya station	Tochigi	1.29, 1.21	<0.001	0.69, 0.63	<0.001	0.83, 0.75	<0.01	0.85, 0.76	<0.001	0.69, 0.61	<0.01	0.49, 0.46	<0.05
	Chuo Maebashi Station	Gunma	–	–	–	–	–	–	–	–	–	–	–	–
	Shibuya Station	Tokyo	0.99, 1.08	<0.001	0.67, 0.63	<0.01	0.62, 0.57	<0.001	0.61, 0.54	<0.001	0.44, 0.41	<0.001	0.51, 0.46	<0.001
	Shibuya Center Street	Tokyo	0.93, 1.02	<0.001	0.61, 0.59	<0.05	0.57, 0.52	<0.001	0.55, 0.49	<0.001	0.39, 0.37	<0.001	0.47, 0.42	<0.001
	Harajuku Station	Tokyo	1.29, 1.41	<0.001	0.86, 0.83	<0.001	0.77, 0.71	<0.001	0.75, 0.67	<0.001	0.55, 0.49	<0.001	0.78, 0.68	<0.001
	Shinjuku Kabukicho	Tokyo	0.81, 0.90	<0.001	0.63, 0.65	<0.001	0.61, 0.59	<0.05	0.60, 0.58	<0.001	0.46, 0.45	<0.001	0.45, 0.41	<0.001
	Ikebukuro Station	Tokyo	0.95, 1.04	<0.001	0.65, 0.64	<0.001	0.70, 0.65	<0.001	0.70, 0.64	<0.001	0.55, 0.50	<0.001	0.57, 0.47	<0.001
	Tokyo Station	Tokyo	1.24, 1.15	<0.01	0.93, 0.85	<0.001	0.93, 0.84	<0.001	0.92, 0.82	<0.001	0.58, 0.56	<0.001	0.53, 0.44	<0.001
	Shinbashi Station	Tokyo	1.10, 1.04	<0.001	0.87, 0.76	<0.001	0.82, 0.73	<0.001	0.83, 0.75	<0.001	0.65, 0.61	<0.001	0.63, 0.58	<0.001
	Shinagawa Station	Tokyo	0.98, 0.88	<0.001	0.79, 0.70	<0.001	0.80, 0.69	<0.001	0.77, 0.68	<0.001	0.58, 0.54	<0.001	0.47, 0.46	<0.01
	Ueno Station	Tokyo	0.89, 0.94	<0.001	0.72, 0.70	<0.001	0.71, 0.66	<0.001	0.70, 0.64	<0.001	0.50, 0.45	<0.001	0.39, 0.35	<0.001
	Ginza Station	Tokyo	1.60, 1.71	<0.001	1.02, 1.02	0.82	1.05, 1.01	<0.001	1.00, 0.91	<0.001	0.71, 0.65	<0.001	0.63, 0.53	<0.001
	Roppongi Station	Tokyo	1.05, 1.09	<0.001	0.89, 0.87	0.30	0.87, 0.87	0.38	0.89, 0.87	<0.05	0.66, 0.69	<0.001	0.79, 0.77	0.08
	Kichijoji Station	Tokyo	1.13, 1.24	<0.001	0.76, 0.76	<0.01	0.78, 0.76	<0.001	0.78, 0.73	<0.001	0.66, 0.59	<0.001	0.62, 0.56	<0.001

	Motomachi-Chukagai Station	Kanagawa	1.03, 0.94	<0.001	0.79, 0.80	0.08	0.92, 0.91	<0.05	0.94, 0.94	0.74	0.75, 0.73	0.06	0.74, 0.62	<0.001
	Sakuragicho Station	Kanagawa	1.40, 1.40	0.50	1.11, 1.04	<0.001	1.01, 0.97	<0.001	1.05, 1.00	<0.001	0.86, 0.78	<0.001	0.72, 0.63	<0.001
	Kannai Station	Kanagawa	1.37, 1.34	0.07	0.86, 0.84	0.12	0.92, 0.85	<0.001	0.96, 0.92	0.07	0.89, 0.83	<0.001	0.84, 0.74	<0.001
	Hamamatsu Station	Shizuoka	–	–	–	–	–	–	–	–	–	–	–	–
	Sakae Station	Aichi	1.02, 1.02	0.65	0.80, 0.75	<0.01	0.80, 0.75	<0.05	0.79, 0.74	<0.01	0.56, 0.54	<0.05	0.48, 0.44	<0.05
	Fushimi Station	Aichi	1.17, 1.18	0.95	0.93, 0.83	<0.01	0.93, 0.88	<0.05	0.90, 0.87	0.12	0.67, 0.66	0.15	0.64, 0.58	<0.01
	Kawaramachi Station	Kyoto	1.04, 0.94	<0.001	0.60, 0.57	<0.001	0.60, 0.56	<0.001	0.63, 0.56	<0.001	0.47, 0.42	<0.001	0.44, 0.39	<0.001
	Shinsaibashi Station	Osaka	1.19, 1.18	0.33	1.05, 1.02	0.55	1.09, 1.05	0.35	1.03, 1.02	0.49	0.72, 0.72	0.54	0.73, 0.72	0.27
	Namba Station	Osaka	0.80, 0.80	0.92	0.62, 0.59	<0.001	0.59, 0.58	<0.001	0.58, 0.56	<0.05	0.44, 0.43	0.30	0.44, 0.43	<0.001
	Kitashinchi Station	Osaka	1.42, 1.38	0.20	1.02, 0.97	<0.001	1.01, 0.95	<0.001	0.99, 0.97	0.19	0.77, 0.76	0.07	0.73, 0.68	<0.05
	Tamachi Station	Okayama	–	–	–	–	–	–	–	–	–	–	–	–
	Hatchobori Station	Hiroshima	–	–	–	–	–	–	–	–	–	–	–	–
	Kawaramachi Station	Kagawa	–	–	–	–	–	–	–	–	–	–	–	–
	Hakata Station	Fukuoka	0.87, 0.75	<0.001	0.76, 0.68	<0.001	0.87, 0.76	<0.001	0.76, 0.66	<0.001	0.65, 0.56	<0.001	0.63, 0.50	<0.001
	Nakasu-kawabata Station	Fukuoka	1.05, 0.94	<0.001	1.06, 1.02	<0.01	1.23, 1.12	<0.001	1.25, 1.18	<0.001	1.00, 0.95	<0.01	0.77, 0.74	0.30
	Kanko-dori Station	Nagasaki	–	–	–	–	–	–	–	–	–	–	–	–
	Makishi Station	Okinawa	–	–	–	–	–	–	–	–	–	–	–	–
16:00−19:59 PM														
	Susukino Station	Hokkaido	–	–	–	–	–	–	–	–	–	–	–	–
	Odori Station	Hokkaido	–	–	–	–	–	–	–	–	–	–	–	–
	Central Hirosaki Station	Aomori	–	–	–	–	–	–	–	–	–	–	–	–
	Kotodai-Koen Station	Miyagi	–	–	–	–	–	–	–	–	–	–	–	–
	Tobu-Utsunomiya station	Tochigi	0.79, 0.60	<0.001	0.64, 0.56	<0.001	0.68, 0.61	<0.01	0.75, 0.65	<0.001	0.63, 0.53	<0.01	0.44, 0.45	0.98
	Chuo Maebashi Station	Gunma	–	–	–	–	–	–	–	–	–	–	–	–
	Shibuya Station	Tokyo	0.88, 0.89	0.89	0.56, 0.52	0.43	0.53, 0.49	<0.001	0.51, 0.48	<0.001	0.37, 0.36	0.12	0.54, 0.46	<0.001
	Shibuya Center Street	Tokyo	0.82, 0.82	0.95	0.50, 0.47	0.40	0.47, 0.43	<0.001	0.45, 0.42	<0.001	0.32, 0.32	0.30	0.50, 0.42	<0.001
	Harajuku Station	Tokyo	1.06, 1.07	0.98	0.70, 0.67	<0.001	0.64, 0.59	<0.001	0.61, 0.56	<0.001	0.47, 0.46	<0.05	0.82, 0.69	<0.001
	Shinjuku Kabukicho	Tokyo	0.72, 0.74	<0.001	0.56, 0.55	0.55	0.57, 0.53	<0.001	0.57, 0.54	<0.05	0.40, 0.40	0.39	0.45, 0.41	<0.001
	Ikebukuro Station	Tokyo	0.89, 0.87	<0.05	0.60, 0.57	<0.001	0.63, 0.58	<0.001	0.61, 0.57	<0.001	0.45, 0.43	<0.001	0.55, 0.47	<0.001
	Tokyo Station	Tokyo	1.05, 0.90	<0.001	0.75, 0.68	<0.001	0.73, 0.65	<0.001	0.72, 0.64	<0.001	0.46, 0.45	<0.01	0.47, 0.39	<0.001
	Shinbashi Station	Tokyo	1.05, 0.89	<0.001	0.76, 0.67	<0.001	0.71, 0.64	<0.001	0.71, 0.67	<0.001	0.51, 0.49	<0.01	0.49, 0.45	<0.001

	Shinagawa Station	Tokyo	0.83, 0.70	<0.001	0.64, 0.58	<0.001	0.65, 0.58	<0.001	0.65, 0.59	<0.001	0.46, 0.47	0.11	0.48, 0.44	<0.001
	Ueno Station	Tokyo	0.81, 0.77	<0.001	0.66, 0.61	<0.001	0.66, 0.60	<0.001	0.65, 0.59	<0.001	0.44, 0.43	<0.01	0.40, 0.36	<0.001
	Ginza Station	Tokyo	1.27, 1.20	<0.001	0.87, 0.82	<0.001	0.85, 0.79	<0.001	0.82, 0.74	<0.001	0.56, 0.53	<0.001	0.50, 0.41	<0.001
	Roppongi Station	Tokyo	1.00, 1.02	0.65	0.82, 0.82	0.83	0.76, 0.77	<0.05	0.79, 0.79	0.24	0.61, 0.62	0.76	0.61, 0.57	<0.001
	Kichijoji Station	Tokyo	0.92, 0.96	<0.001	0.65, 0.63	<0.01	0.62, 0.60	<0.001	0.61, 0.58	<0.001	0.47, 0.44	<0.001	0.50, 0.44	<0.001
	Motomachi-Chukagai Station	Kanagawa	0.94, 0.90	<0.001	0.67, 0.67	1.00	0.76, 0.70	<0.001	0.79, 0.80	0.09	0.59, 0.59	0.19	0.64, 0.55	<0.001
	Sakuragicho Station	Kanagawa	1.12, 1.05	<0.001	0.84, 0.76	<0.001	0.82, 0.75	<0.001	0.85, 0.77	<0.001	0.62, 0.58	<0.001	0.62, 0.54	<0.001
	Kannai Station	Kanagawa	1.10, 1.05	<0.001	0.81, 0.78	<0.001	0.88, 0.81	<0.001	0.88, 0.85	<0.01	0.74, 0.69	<0.001	0.71, 0.62	<0.001
	Hamamatsu Station	Shizuoka	–	–	–	–	–	–	–	–	–	–	–	–
	Sakae Station	Aichi	0.80, 0.76	<0.001	0.69, 0.64	<0.001	0.65, 0.60	<0.01	0.61, 0.58	<0.01	0.42, 0.42	0.85	0.46, 0.40	<0.01
	Fushimi Station	Aichi	1.02, 0.96	<0.01	0.78, 0.69	<0.001	0.79, 0.72	<0.01	0.72, 0.68	<0.05	0.46, 0.45	0.08	0.51, 0.46	<0.01
	Kawaramachi Station	Kyoto	0.80, 0.70	<0.001	0.47, 0.42	<0.001	0.55, 0.48	<0.001	0.54, 0.47	<0.001	0.39, 0.35	<0.001	0.49, 0.39	<0.001
	Shinsaibashi Station	Osaka	1.00, 0.93	<0.001	0.86, 0.81	<0.001	0.91, 0.85	<0.01	0.88, 0.83	0.11	0.58, 0.60	<0.01	0.62, 0.59	<0.01
	Namba Station	Osaka	0.72, 0.68	<0.001	0.58, 0.55	<0.001	0.57, 0.54	<0.001	0.57, 0.55	<0.01	0.40, 0.40	0.43	0.43, 0.41	<0.001
	Kitashinchi Station	Osaka	1.11, 0.99	<0.001	0.79, 0.73	<0.001	0.83, 0.74	<0.001	0.79, 0.75	<0.01	0.56, 0.55	0.73	0.62, 0.56	<0.001
	Tamachi Station	Okayama	–	–	–	–	–	–	–	–	–	–	–	–
	Hatchobori Station	Hiroshima	–	–	–	–	–	–	–	–	–	–	–	–
	Kawaramachi Station	Kagawa	–	–	–	–	–	–	–	–	–	–	–	–
	Hakata Station	Fukuoka	0.75, 0.61	<0.001	0.69, 0.59	<0.001	0.75, 0.62	<0.001	0.65, 0.55	<0.001	0.51, 0.43	<0.001	0.61, 0.45	<0.001
	Nakasu-kawabata Station	Fukuoka	0.85, 0.72	<0.001	0.93, 0.84	<0.001	0.99, 0.85	<0.001	0.94, 0.85	<0.001	0.65, 0.61	<0.01	0.72, 0.62	<0.01
	Kanko-dori Station	Nagasaki	–	–	–	–	–	–	–	–	–	–	–	–
	Makishi Station	Okinawa	–	–	–	–	–	–	–	–	–	–	–	–
20:00−23:59 PM														
	Susukino Station	Hokkaido	–	–	–	–	–	–	–	–	–	–	–	–
	Odori Station	Hokkaido	–	–	–	–	–	–	–	–	–	–	–	–
	Central Hirosaki Station	Aomori	–	–	–	–	–	–	–	–	–	–	–	–
	Kotodai-Koen Station	Miyagi	–	–	–	–	–	–	–	–	–	–	–	–
	Tobu-Utsunomiya station	Tochigi	0.49, 0.28	<0.001	0.33, 0.31	<0.01	0.38, 0.38	0.85	0.43, 0.41	<0.05	0.33, 0.28	<0.01	0.45, 0.30	<0.001
	Chuo Maebashi Station	Gunma	–	–	–	–	–	–	–	–	–	–	–	–
	Shibuya Station	Tokyo	0.64, 0.47	<0.001	0.33, 0.30	<0.001	0.32, 0.30	<0.001	0.29, 0.26	<0.001	0.21, 0.20	<0.001	0.53, 0.35	<0.001
	Shibuya Center Street	Tokyo	0.61, 0.44	<0.001	0.30, 0.27	<0.001	0.29, 0.27	<0.001	0.26, 0.24	<0.001	0.19, 0.17	<0.001	0.50, 0.32	<0.001

Harajuku Station	Tokyo	0.77, 0.64	<0.001	0.47, 0.44	<0.001	0.44, 0.43	<0.05	0.36, 0.35	<0.001	0.31, 0.28	<0.001	0.67, 0.57	<0.001
Shinjuku Kabukicho	Tokyo	0.56, 0.42	<0.001	0.37, 0.30	<0.001	0.36, 0.31	<0.001	0.34, 0.30	<0.001	0.23, 0.21	<0.001	0.47, 0.32	<0.001
Ikebukuro Station	Tokyo	0.62, 0.47	<0.001	0.39, 0.31	<0.001	0.40, 0.31	<0.001	0.36, 0.28	<0.001	0.27, 0.21	<0.001	0.48, 0.30	<0.001
Tokyo Station	Tokyo	0.72, 0.49	<0.001	0.46, 0.38	<0.001	0.44, 0.37	<0.001	0.39, 0.32	<0.001	0.25, 0.19	<0.001	0.40, 0.26	<0.001
Shinbashi Station	Tokyo	0.79, 0.52	<0.001	0.47, 0.38	<0.001	0.45, 0.36	<0.001	0.40, 0.32	<0.001	0.28, 0.21	<0.001	0.39, 0.28	<0.001
Shinagawa Station	Tokyo	0.62, 0.44	<0.001	0.46, 0.39	<0.001	0.44, 0.38	<0.001	0.41, 0.35	<0.001	0.27, 0.26	<0.01	0.39, 0.32	<0.001
Ueno Station	Tokyo	0.61, 0.50	<0.001	0.43, 0.37	<0.001	0.44, 0.37	<0.001	0.39, 0.33	<0.001	0.25, 0.20	<0.001	0.34, 0.25	<0.001
Ginza Station	Tokyo	0.84, 0.55	<0.001	0.52, 0.38	<0.001	0.47, 0.35	<0.001	0.43, 0.31	<0.001	0.28, 0.19	<0.001	0.36, 0.22	<0.001
Roppongi Station	Tokyo	0.71, 0.58	<0.001	0.53, 0.50	<0.001	0.46, 0.41	<0.001	0.44, 0.41	<0.001	0.36, 0.29	<0.001	0.48, 0.39	<0.001
Kichijoji Station	Tokyo	0.56, 0.46	<0.001	0.40, 0.33	<0.001	0.40, 0.36	<0.001	0.36, 0.30	<0.001	0.26, 0.22	<0.001	0.42, 0.36	<0.001
Motomachi-Chukagai Station	Kanagawa	0.51, 0.44	<0.001	0.41, 0.39	<0.001	0.43, 0.38	<0.001	0.53, 0.54	<0.001	0.45, 0.44	<0.05	0.46, 0.43	<0.001
Sakuragicho Station	Kanagawa	0.78, 0.57	<0.001	0.50, 0.41	<0.001	0.47, 0.41	<0.001	0.44, 0.38	<0.001	0.37, 0.32	<0.001	0.46, 0.37	<0.001
Kannai Station	Kanagawa	0.65, 0.55	<0.001	0.53, 0.45	<0.001	0.58, 0.46	<0.001	0.46, 0.39	<0.001	0.39, 0.28	<0.001	0.52, 0.38	<0.001
Hamamatsu Station	Shizuoka	–	–	–	–	–	–	–	–	–	–	–	–
Sakae Station	Aichi	0.45, 0.38	<0.001	0.36, 0.33	0.08	0.35, 0.32	0.05	0.30, 0.26	<0.01	0.20, 0.18	<0.01	0.26, 0.20	<0.001
Fushimi Station	Aichi	0.61, 0.51	<0.001	0.40, 0.34	<0.05	0.39, 0.34	<0.05	0.31, 0.29	0.17	0.21, 0.20	<0.05	0.28, 0.23	<0.001
Kawaramachi Station	Kyoto	0.60, 0.34	<0.001	0.26, 0.19	<0.001	0.31, 0.22	<0.001	0.30, 0.23	<0.001	0.24, 0.17	<0.001	0.41, 0.27	<0.001
Shinsaibashi Station	Osaka	0.57, 0.47	<0.001	0.40, 0.35	<0.001	0.43, 0.39	<0.001	0.36, 0.33	<0.001	0.25, 0.24	<0.001	0.39, 0.33	<0.001
Namba Station	Osaka	0.46, 0.36	<0.001	0.33, 0.27	<0.001	0.34, 0.28	<0.001	0.30, 0.26	<0.001	0.21, 0.18	<0.001	0.36, 0.29	<0.001
Kitashinchi Station	Osaka	0.55, 0.40	<0.001	0.39, 0.32	<0.001	0.40, 0.31	<0.001	0.33, 0.27	<0.001	0.23, 0.20	<0.001	0.33, 0.23	<0.001
Tamachi Station	Okayama	–	–	–	–	–	–	–	–	–	–	–	–
Hatchobori Station	Hiroshima	–	–	–	–	–	–	–	–	–	–	–	–
Kawaramachi Station	Kagawa	–	–	–	–	–	–	–	–	–	–	–	–
Hakata Station	Fukuoka	0.48, 0.28	<0.001	0.44, 0.30	<0.001	0.47, 0.33	<0.01	0.37, 0.26	<0.001	0.28, 0.22	<0.001	0.38, 0.26	<0.001
Nakasu-kawabata Station	Fukuoka	0.45, 0.32	<0.001	0.45, 0.34	<0.001	0.49, 0.34	<0.001	0.44, 0.30	<0.001	0.33, 0.23	<0.001	0.43, 0.31	<0.001
Kanko-dori Station	Nagasaki	–	–	–	–	–	–	–	–	–	–	–	–
Makishi Station	Okinawa	–	–	–	–	–	–	–	–	–	–	–	–

Table S3: P-values for the comparison of human mobility among age groups using the Kruskal-Wallis test, by time frames and by the first and second declaration of a state of emergency. The ratios of the rolling seven-day daily average of the total population to a baseline on January 16, 2020 during the declaration of a state of emergency was compared among age groups.

				Median ratio by age group						
Declaration	Time frame	Area	Prefecture	20s	30s	40s	50s	60s	≥ 70s	P value
First										
	0:00−7:59 AM									
		Susukino Station	Hokkaido	0.33	0.27	0.34	0.37	0.36	0.53	<0.001
		Odori Station	Hokkaido	0.46	0.73	0.69	0.75	0.67	0.66	<0.001
		Central Hirosaki Station	Aomori	0.05	0.92	0.75	0.64	0.91	0.86	<0.001
		Kotodai-Koen Station	Miyagi	0.51	0.48	0.66	0.73	0.82	0.57	<0.001
		Tobu-Utsunomiya station	Tochigi	0.54	0.66	0.57	0.76	0.84	0.61	<0.001
		Chuo Maebashi Station	Gunma	1.86	0.52	0.56	0.69	0.45	0.65	<0.001
		Shibuya Station	Tokyo	0.35	0.46	0.60	0.64	0.61	0.59	<0.001
		Shibuya Center Street	Tokyo	0.31	0.41	0.54	0.57	0.54	0.55	<0.001
		Harajuku Station	Tokyo	0.50	0.58	0.83	0.84	0.88	1.17	<0.001
		Shinjuku Kabukicho	Tokyo	0.30	0.55	0.53	0.60	0.64	0.54	<0.001
		Ikebukuro Station	Tokyo	0.50	0.72	0.76	0.82	0.75	0.69	<0.001
		Tokyo Station	Tokyo	0.38	0.60	0.60	0.61	0.58	0.45	<0.001
		Shinbashi Station	Tokyo	0.37	0.48	0.56	0.50	0.53	0.47	<0.001
		Shinagawa Station	Tokyo	0.48	0.60	0.61	0.61	0.62	0.51	<0.001
		Ueno Station	Tokyo	0.74	0.74	0.77	0.78	0.66	0.56	<0.001
		Ginza Station	Tokyo	0.44	0.48	0.58	0.55	0.51	0.40	<0.001
		Roppongi Station	Tokyo	0.28	0.49	0.62	0.58	0.59	0.52	<0.001
		Kichijoji Station	Tokyo	0.74	0.56	0.75	0.72	0.71	0.90	<0.001
		Motomachi-Chukagai Station	Kanagawa	0.69	0.80	0.79	0.81	1.01	0.65	<0.001
		Sakuragicho Station	Kanagawa	0.47	0.79	0.85	0.75	0.70	0.61	<0.001
		Kannai Station	Kanagawa	0.92	0.72	0.88	0.84	0.74	1.18	<0.001
		Hamamatsu Station	Shizuoka	0.31	0.61	0.55	0.70	0.71	0.78	<0.001
		Sakae Station	Aichi	0.48	0.47	0.51	0.50	0.57	0.41	<0.01
		Fushimi Station	Aichi	0.47	0.66	0.66	0.82	0.71	0.42	<0.001
		Kawaramachi Station	Kyoto	0.29	0.39	0.54	0.57	0.51	0.71	<0.001
		Shinsaibashi Station	Osaka	0.32	0.43	0.54	0.53	0.56	0.53	<0.001
		Namba Station	Osaka	0.35	0.38	0.52	0.52	0.50	0.43	<0.001
		Kitashinchi Station	Osaka	0.27	0.33	0.38	0.46	0.43	0.46	<0.001
		Tamachi Station	Okayama	0.68	0.42	0.81	0.70	0.63	0.69	<0.001
		Hatchobori Station	Hiroshima	0.32	0.37	0.47	0.41	0.45	0.38	<0.001
		Kawaramachi Station	Kagawa	0.47	0.84	1.07	1.63	1.46	1.45	<0.001
		Hakata Station	Fukuoka	0.35	0.46	0.58	0.51	0.50	0.46	<0.001
		Nakasu-kawabata Station	Fukuoka	0.48	0.29	0.39	0.53	0.54	0.40	<0.001
		Kanko-dori Station	Nagasaki	0.73	0.69	0.58	0.74	0.82	0.61	<0.001
		Makishi Station	Okinawa	0.85	0.60	1.07	0.73	0.91	0.85	<0.001
	08:00−11:59 AM									
		Susukino Station	Hokkaido	0.65	0.67	0.70	0.73	0.63	0.60	<0.001
		Odori Station	Hokkaido	0.51	0.69	0.66	0.64	0.58	0.49	<0.001
		Central Hirosaki Station	Aomori	0.49	0.84	0.78	0.87	1.12	0.91	<0.001
		Kotodai-Koen Station	Miyagi	0.56	0.77	0.69	0.72	0.71	0.62	<0.001

	Tobu-Utsunomiya station	Tochigi	0.80	0.76	0.64	0.76	0.72	0.51	<0.001
	Chuo Maebashi Station	Gunma	1.63	0.39	0.62	0.79	0.54	0.81	<0.001
	Shibuya Station	Tokyo	0.32	0.44	0.50	0.55	0.48	0.38	<0.001
	Shibuya Center Street	Tokyo	0.29	0.41	0.46	0.50	0.44	0.36	<0.001
	Harajuku Station	Tokyo	0.38	0.57	0.57	0.66	0.60	0.55	<0.001
	Shinjuku Kabukicho	Tokyo	0.40	0.51	0.52	0.53	0.48	0.36	<0.001
	Ikebukuro Station	Tokyo	0.47	0.59	0.65	0.68	0.59	0.45	<0.001
	Tokyo Station	Tokyo	0.34	0.44	0.45	0.43	0.39	0.30	<0.001
	Shinbashi Station	Tokyo	0.39	0.39	0.46	0.44	0.46	0.39	<0.001
	Shinagawa Station	Tokyo	0.42	0.40	0.39	0.39	0.39	0.33	<0.001
	Ueno Station	Tokyo	0.48	0.58	0.61	0.61	0.50	0.38	<0.001
	Ginza Station	Tokyo	0.45	0.42	0.48	0.47	0.42	0.31	<0.001
	Roppongi Station	Tokyo	0.50	0.53	0.60	0.64	0.59	0.46	<0.001
	Kichijoji Station	Tokyo	0.54	0.57	0.66	0.67	0.66	0.55	<0.001
	Motomachi-Chukagai Station	Kanagawa	0.84	0.75	0.81	0.85	0.81	0.70	<0.001
	Sakuragicho Station	Kanagawa	0.68	0.66	0.73	0.72	0.65	0.47	<0.001
	Kannai Station	Kanagawa	0.74	0.69	0.73	0.87	0.80	0.71	<0.001
	Hamamatsu Station	Shizuoka	0.57	0.59	0.65	0.61	0.59	0.47	<0.001
	Sakae Station	Aichi	0.44	0.53	0.58	0.61	0.58	0.43	<0.001
	Fushimi Station	Aichi	0.60	0.56	0.70	0.71	0.60	0.42	<0.001
	Kawaramachi Station	Kyoto	0.44	0.57	0.64	0.65	0.54	0.39	<0.001
	Shinsaibashi Station	Osaka	0.52	0.57	0.69	0.67	0.64	0.59	<0.001
	Namba Station	Osaka	0.37	0.46	0.48	0.52	0.46	0.34	<0.001
	Kitashinchi Station	Osaka	0.53	0.51	0.57	0.60	0.56	0.41	<0.001
	Tamachi Station	Okayama	0.84	0.87	0.95	0.89	0.76	0.69	<0.001
	Hatchobori Station	Hiroshima	0.55	0.62	0.68	0.67	0.58	0.53	<0.01
	Kawaramachi Station	Kagawa	0.72	0.84	0.77	0.90	0.77	0.89	<0.001
	Hakata Station	Fukuoka	0.41	0.45	0.50	0.47	0.46	0.36	<0.001
	Nakasu-kawabata Station	Fukuoka	0.63	0.61	0.66	0.74	0.63	0.55	<0.001
	Kanko-dori Station	Nagasaki	0.84	0.71	0.78	0.81	0.77	0.78	<0.01
	Makishi Station	Okinawa	1.11	0.89	0.93	0.84	0.95	0.69	<0.001
12:00−15:59 PM									
	Susukino Station	Hokkaido	0.62	0.66	0.74	0.76	0.68	0.44	<0.001
	Odori Station	Hokkaido	0.55	0.73	0.67	0.64	0.57	0.37	<0.001
	Central Hirosaki Station	Aomori	1.07	0.82	0.87	0.93	0.93	0.92	<0.001
	Kotodai-Koen Station	Miyagi	0.64	0.72	0.72	0.76	0.68	0.42	<0.001
	Tobu-Utsunomiya station	Tochigi	1.18	0.58	0.55	0.71	0.60	0.34	<0.001
	Chuo Maebashi Station	Gunma	1.79	0.46	0.57	0.80	0.77	0.67	<0.001
	Shibuya Station	Tokyo	0.26	0.41	0.46	0.49	0.42	0.29	<0.001
	Shibuya Center Street	Tokyo	0.24	0.38	0.43	0.45	0.37	0.27	<0.001
	Harajuku Station	Tokyo	0.38	0.52	0.51	0.60	0.54	0.47	<0.001
	Shinjuku Kabukicho	Tokyo	0.30	0.45	0.45	0.46	0.38	0.27	<0.001
	Ikebukuro Station	Tokyo	0.39	0.49	0.54	0.58	0.49	0.34	<0.001
	Tokyo Station	Tokyo	0.32	0.41	0.42	0.42	0.36	0.22	<0.001
	Shinbashi Station	Tokyo	0.35	0.36	0.42	0.40	0.41	0.30	<0.001
	Shinagawa Station	Tokyo	0.43	0.41	0.41	0.40	0.38	0.29	<0.001
	Ueno Station	Tokyo	0.42	0.60	0.58	0.59	0.46	0.32	<0.001
	Ginza Station	Tokyo	0.27	0.32	0.39	0.36	0.28	0.21	<0.001
	Roppongi Station	Tokyo	0.46	0.52	0.58	0.57	0.51	0.44	<0.001

	Kichijoji Station	Tokyo	0.49	0.53	0.56	0.61	0.59	0.43	<0.001
	Motomachi-Chukagai Station	Kanagawa	0.85	0.72	0.94	0.88	0.78	0.68	<0.001
	Sakuragicho Station	Kanagawa	0.56	0.64	0.70	0.69	0.55	0.39	<0.001
	Kannai Station	Kanagawa	0.86	0.70	0.70	0.86	0.80	0.59	<0.001
	Hamamatsu Station	Shizuoka	0.50	0.53	0.61	0.59	0.54	0.43	<0.001
	Sakae Station	Aichi	0.43	0.55	0.61	0.67	0.52	0.37	<0.001
	Fushimi Station	Aichi	0.62	0.57	0.74	0.72	0.56	0.41	<0.001
	Kawaramachi Station	Kyoto	0.35	0.47	0.50	0.53	0.39	0.34	<0.001
	Shinsaibashi Station	Osaka	0.43	0.52	0.65	0.61	0.55	0.43	<0.001
	Namba Station	Osaka	0.30	0.37	0.38	0.38	0.32	0.27	<0.001
	Kitashinchi Station	Osaka	0.45	0.52	0.54	0.56	0.49	0.33	<0.001
	Tamachi Station	Okayama	0.81	0.93	0.91	0.87	0.73	0.56	<0.001
	Hatchobori Station	Hiroshima	0.51	0.55	0.63	0.64	0.52	0.43	<0.001
	Kawaramachi Station	Kagawa	0.59	0.75	0.88	0.94	0.84	0.72	<0.001
	Hakata Station	Fukuoka	0.36	0.37	0.45	0.38	0.38	0.29	<0.001
	Nakasu-kawabata Station	Fukuoka	0.58	0.63	0.73	0.78	0.62	0.43	<0.001
	Kanko-dori Station	Nagasaki	0.71	0.67	0.72	0.75	0.68	0.61	<0.001
	Makishi Station	Okinawa	1.04	0.91	0.87	0.94	0.91	0.85	<0.001
16:00–19:59 PM									
	Susukino Station	Hokkaido	0.47	0.49	0.55	0.54	0.44	0.35	<0.001
	Odori Station	Hokkaido	0.53	0.64	0.61	0.57	0.50	0.41	<0.001
	Central Hirosaki Station	Aomori	0.48	0.81	0.73	0.75	1.06	0.94	<0.001
	Kotodai-Koen Station	Miyagi	0.60	0.67	0.66	0.71	0.59	0.43	<0.001
	Tobu-Utsunomiya station	Tochigi	0.84	0.61	0.52	0.68	0.63	0.49	<0.001
	Chuo Maebashi Station	Gunma	1.39	0.34	0.51	0.62	0.48	0.92	<0.001
	Shibuya Station	Tokyo	0.24	0.42	0.46	0.52	0.45	0.32	<0.001
	Shibuya Center Street	Tokyo	0.22	0.38	0.42	0.48	0.40	0.29	<0.001
	Harajuku Station	Tokyo	0.34	0.58	0.60	0.68	0.65	0.47	<0.001
	Shinjuku Kabukicho	Tokyo	0.27	0.42	0.45	0.48	0.42	0.25	<0.001
	Ikebukuro Station	Tokyo	0.44	0.53	0.59	0.63	0.55	0.38	<0.001
	Tokyo Station	Tokyo	0.30	0.40	0.43	0.44	0.38	0.22	<0.001
	Shinbashi Station	Tokyo	0.34	0.37	0.43	0.43	0.41	0.24	<0.001
	Shinagawa Station	Tokyo	0.42	0.38	0.42	0.42	0.41	0.33	<0.001
	Ueno Station	Tokyo	0.43	0.57	0.61	0.62	0.51	0.32	<0.001
	Ginza Station	Tokyo	0.27	0.31	0.37	0.36	0.29	0.20	<0.001
	Roppongi Station	Tokyo	0.44	0.50	0.57	0.58	0.53	0.37	<0.001
	Kichijoji Station	Tokyo	0.46	0.58	0.60	0.65	0.59	0.43	<0.001
	Motomachi-Chukagai Station	Kanagawa	0.89	0.74	0.83	0.83	0.79	0.66	<0.001
	Sakuragicho Station	Kanagawa	0.50	0.62	0.68	0.67	0.59	0.39	<0.001
	Kannai Station	Kanagawa	0.81	0.74	0.76	0.88	0.78	0.51	<0.001
	Hamamatsu Station	Shizuoka	0.46	0.60	0.64	0.61	0.57	0.52	<0.001
	Sakae Station	Aichi	0.38	0.56	0.55	0.58	0.48	0.35	<0.001
	Fushimi Station	Aichi	0.60	0.58	0.70	0.69	0.54	0.31	<0.001
	Kawaramachi Station	Kyoto	0.29	0.44	0.51	0.51	0.40	0.30	<0.001
	Shinsaibashi Station	Osaka	0.43	0.49	0.61	0.62	0.56	0.37	<0.001
	Namba Station	Osaka	0.27	0.40	0.43	0.44	0.37	0.25	<0.001
	Kitashinchi Station	Osaka	0.38	0.46	0.50	0.52	0.44	0.30	<0.001
	Tamachi Station	Okayama	0.70	0.80	0.82	0.79	0.71	0.56	<0.001
	Hatchobori Station	Hiroshima	0.39	0.44	0.52	0.52	0.45	0.33	<0.001

		Kawaramachi Station	Kagawa	0.61	0.71	0.80	0.94	0.76	0.71	<0.001
		Hakata Station	Fukuoka	0.31	0.38	0.42	0.41	0.42	0.27	<0.001
		Nakasu-kawabata Station	Fukuoka	0.50	0.59	0.57	0.61	0.50	0.42	<0.001
		Kanko-dori Station	Nagasaki	0.59	0.67	0.72	0.75	0.66	0.52	<0.001
		Makishi Station	Okinawa	1.17	0.84	0.89	0.85	0.92	0.80	<0.001
	20:00–23:59 PM									
		Susukino Station	Hokkaido	0.27	0.22	0.25	0.24	0.17	0.22	<0.001
		Odori Station	Hokkaido	0.36	0.50	0.47	0.43	0.32	0.35	<0.001
		Central Hirosaki Station	Aomori	0.15	0.60	0.47	0.46	0.46	0.72	<0.001
		Kotodai-Koen Station	Miyagi	0.37	0.45	0.59	0.55	0.37	0.45	<0.001
		Tobu-Utsunomiya station	Tochigi	0.67	0.46	0.41	0.55	0.52	0.47	<0.001
		Chuo Maebashi Station	Gunma	1.27	0.37	0.42	0.48	0.39	0.64	<0.001
		Shibuya Station	Tokyo	0.16	0.33	0.35	0.36	0.27	0.24	<0.001
		Shibuya Center Street	Tokyo	0.15	0.28	0.31	0.32	0.24	0.22	<0.001
		Harajuku Station	Tokyo	0.24	0.41	0.50	0.45	0.41	0.35	<0.001
		Shinjuku Kabukicho	Tokyo	0.16	0.30	0.33	0.34	0.27	0.24	<0.001
		Ikebukuro Station	Tokyo	0.28	0.42	0.45	0.44	0.34	0.25	<0.001
		Tokyo Station	Tokyo	0.23	0.34	0.37	0.34	0.25	0.17	<0.001
		Shinbashi Station	Tokyo	0.22	0.26	0.32	0.28	0.22	0.16	<0.001
		Shinagawa Station	Tokyo	0.26	0.30	0.35	0.32	0.29	0.23	<0.001
		Ueno Station	Tokyo	0.37	0.41	0.45	0.43	0.28	0.27	<0.001
		Ginza Station	Tokyo	0.20	0.22	0.27	0.24	0.17	0.12	<0.001
		Roppongi Station	Tokyo	0.32	0.37	0.44	0.41	0.34	0.27	<0.001
		Kichijoji Station	Tokyo	0.32	0.39	0.48	0.43	0.39	0.39	<0.001
		Motomachi-Chukagai Station	Kanagawa	0.62	0.67	0.61	0.66	0.75	0.62	<0.001
		Sakuragicho Station	Kanagawa	0.39	0.48	0.54	0.45	0.40	0.28	<0.001
		Kannai Station	Kanagawa	0.54	0.63	0.59	0.59	0.43	0.36	<0.001
		Hamamatsu Station	Shizuoka	0.28	0.52	0.50	0.43	0.44	0.65	<0.001
		Sakae Station	Aichi	0.24	0.32	0.38	0.36	0.32	0.20	<0.001
		Fushimi Station	Aichi	0.38	0.44	0.47	0.50	0.39	0.19	<0.001
		Kawaramachi Station	Kyoto	0.20	0.31	0.35	0.38	0.29	0.21	<0.001
		Shinsaibashi Station	Osaka	0.33	0.34	0.39	0.37	0.30	0.29	<0.001
		Namba Station	Osaka	0.17	0.27	0.30	0.28	0.24	0.16	<0.001
		Kitashinchi Station	Osaka	0.20	0.24	0.27	0.26	0.20	0.16	<0.001
		Tamachi Station	Okayama	0.52	0.50	0.59	0.51	0.46	0.52	<0.01
		Hatchobori Station	Hiroshima	0.25	0.24	0.27	0.27	0.22	0.18	<0.001
		Kawaramachi Station	Kagawa	0.36	0.68	0.80	0.87	0.69	0.86	<0.001
		Hakata Station	Fukuoka	0.18	0.25	0.30	0.24	0.27	0.22	<0.001
		Nakasu-kawabata Station	Fukuoka	0.33	0.27	0.29	0.30	0.26	0.31	<0.001
		Kanko-dori Station	Nagasaki	0.46	0.39	0.47	0.52	0.50	0.26	<0.001
		Makishi Station	Okinawa	0.96	0.62	0.87	0.69	0.78	0.71	<0.001
Second										
	0:00–7:59 AM									
		Susukino Station	Hokkaido	–	–	–	–	–	–	–
		Odori Station	Hokkaido	–	–	–	–	–	–	–
		Central Hirosaki Station	Aomori	–	–	–	–	–	–	–
		Kotodai-Koen Station	Miyagi	–	–	–	–	–	–	–
		Tobu-Utsunomiya station	Tochigi	0.25	0.39	0.57	0.61	0.56	0.40	<0.001
		Chuo Maebashi Station	Gunma	–	–	–	–	–	–	–

	Shibuya Station	Tokyo	0.44	0.29	0.36	0.36	0.38	0.52	<0.001
	Shibuya Center Street	Tokyo	0.41	0.26	0.32	0.32	0.34	0.47	<0.001
	Harajuku Station	Tokyo	0.63	0.44	0.55	0.48	0.48	0.79	<0.001
	Shinjuku Kabukicho	Tokyo	0.47	0.39	0.40	0.46	0.41	0.56	<0.001
	Ikebukuro Station	Tokyo	0.53	0.40	0.44	0.47	0.41	0.49	<0.001
	Tokyo Station	Tokyo	0.50	0.48	0.47	0.50	0.43	0.42	<0.001
	Shinbashi Station	Tokyo	0.48	0.46	0.43	0.48	0.43	0.51	<0.001
	Shinagawa Station	Tokyo	0.52	0.50	0.53	0.59	0.53	0.42	<0.001
	Ueno Station	Tokyo	0.71	0.51	0.58	0.56	0.44	0.41	<0.001
	Ginza Station	Tokyo	0.50	0.52	0.51	0.58	0.46	0.40	<0.001
	Roppongi Station	Tokyo	0.45	0.52	0.47	0.47	0.46	0.67	<0.001
	Kichijoji Station	Tokyo	0.64	0.44	0.67	0.56	0.53	0.62	<0.001
	Motomachi-Chukagai Station	Kanagawa	0.51	0.51	0.51	0.75	0.68	0.54	<0.001
	Sakuragicho Station	Kanagawa	0.56	0.56	0.61	0.64	0.60	0.58	<0.001
	Kannai Station	Kanagawa	0.81	0.62	0.63	0.60	0.60	1.04	<0.001
	Hamamatsu Station	Shizuoka	–	–	–	–	–	–	–
	Sakae Station	Aichi	0.51	0.40	0.40	0.37	0.35	0.35	<0.001
	Fushimi Station	Aichi	0.58	0.52	0.50	0.54	0.49	0.39	<0.001
	Kawaramachi Station	Kyoto	0.38	0.22	0.32	0.37	0.37	0.68	<0.001
	Shinsaibashi Station	Osaka	0.49	0.37	0.44	0.49	0.44	0.49	<0.001
	Namba Station	Osaka	0.47	0.30	0.39	0.44	0.36	0.52	<0.001
	Kitashinchi Station	Osaka	0.36	0.34	0.39	0.44	0.38	0.43	<0.001
	Tamachi Station	Okayama	–	–	–	–	–	–	–
	Hatchobori Station	Hiroshima	–	–	–	–	–	–	–
	Kawaramachi Station	Kagawa	–	–	–	–	–	–	–
	Hakata Station	Fukuoka	0.41	0.43	0.56	0.46	0.41	0.42	<0.001
	Nakasu-kawabata Station	Fukuoka	0.31	0.42	0.51	0.53	0.53	0.56	<0.001
	Kanko-dori Station	Nagasaki	–	–	–	–	–	–	–
	Makishi Station	Okinawa	–	–	–	–	–	–	–
08:00–11:59 AM									
	Susukino Station	Hokkaido	–	–	–	–	–	–	–
	Odori Station	Hokkaido	–	–	–	–	–	–	–
	Central Hirosaki Station	Aomori	–	–	–	–	–	–	–
	Kotodai-Koen Station	Miyagi	–	–	–	–	–	–	–
	Tobu-Utsunomiya station	Tochigi	0.88	0.92	0.83	0.88	0.76	0.51	<0.001
	Chuo Maebashi Station	Gunma	–	–	–	–	–	–	–
	Shibuya Station	Tokyo	0.96	0.61	0.57	0.56	0.46	0.50	<0.001
	Shibuya Center Street	Tokyo	0.91	0.57	0.52	0.51	0.42	0.45	<0.001
	Harajuku Station	Tokyo	1.01	0.76	0.69	0.64	0.51	0.66	<0.001
	Shinjuku Kabukicho	Tokyo	0.92	0.63	0.61	0.59	0.47	0.49	<0.001
	Ikebukuro Station	Tokyo	1.00	0.64	0.63	0.62	0.49	0.52	<0.001
	Tokyo Station	Tokyo	0.95	0.77	0.72	0.70	0.51	0.46	<0.001
	Shinbashi Station	Tokyo	0.91	0.68	0.65	0.66	0.54	0.60	<0.001
	Shinagawa Station	Tokyo	0.80	0.65	0.59	0.59	0.49	0.49	<0.001
	Ueno Station	Tokyo	0.87	0.61	0.59	0.56	0.43	0.36	<0.001
	Ginza Station	Tokyo	1.43	0.95	0.95	0.87	0.69	0.59	<0.001
	Roppongi Station	Tokyo	0.98	0.84	0.80	0.88	0.70	0.77	<0.001
	Kichijoji Station	Tokyo	1.23	0.74	0.79	0.72	0.61	0.62	<0.001
	Motomachi-Chukagai Station	Kanagawa	0.86	0.82	0.86	1.01	0.78	0.64	<0.001

	Sakuragicho Station	Kanagawa	1.26	0.95	0.93	0.95	0.79	0.68	<0.001
	Kannai Station	Kanagawa	1.27	0.89	0.88	0.95	0.85	0.85	<0.001
	Hamamatsu Station	Shizuoka	–	–	–	–	–	–	–
	Sakae Station	Aichi	0.90	0.77	0.75	0.71	0.56	0.50	<0.001
	Fushimi Station	Aichi	1.05	0.89	0.87	0.89	0.66	0.61	<0.001
	Kawaramachi Station	Kyoto	1.05	0.65	0.64	0.63	0.50	0.48	<0.001
	Shinsaibashi Station	Osaka	1.33	1.05	1.08	1.07	0.79	0.88	<0.001
	Namba Station	Osaka	0.87	0.67	0.63	0.64	0.47	0.48	<0.001
	Kitashinchi Station	Osaka	1.30	0.89	0.89	0.86	0.71	0.76	<0.001
	Tamachi Station	Okayama	–	–	–	–	–	–	–
	Hatchobori Station	Hiroshima	–	–	–	–	–	–	–
	Kawaramachi Station	Kagawa	–	–	–	–	–	–	–
	Hakata Station	Fukuoka	0.78	0.69	0.71	0.64	0.53	0.50	<0.001
	Nakasu-kawabata Station	Fukuoka	0.90	1.04	1.08	1.10	0.92	0.79	<0.001
	Kanko-dori Station	Nagasaki	–	–	–	–	–	–	–
	Makishi Station	Okinawa	–	–	–	–	–	–	–
12:00–15:59 PM									
	Susukino Station	Hokkaido	–	–	–	–	–	–	–
	Odori Station	Hokkaido	–	–	–	–	–	–	–
	Central Hirosaki Station	Aomori	–	–	–	–	–	–	–
	Kotodai-Koen Station	Miyagi	–	–	–	–	–	–	–
	Tobu-Utsunomiya station	Tochigi	1.21	0.63	0.75	0.76	0.61	0.46	<0.001
	Chuo Maebashi Station	Gunma	–	–	–	–	–	–	–
	Shibuya Station	Tokyo	1.08	0.63	0.57	0.54	0.41	0.46	<0.001
	Shibuya Center Street	Tokyo	1.02	0.59	0.52	0.49	0.37	0.42	<0.001
	Harajuku Station	Tokyo	1.41	0.83	0.71	0.67	0.49	0.68	<0.001
	Shinjuku Kabukicho	Tokyo	0.90	0.65	0.59	0.58	0.45	0.41	<0.001
	Ikebukuro Station	Tokyo	1.04	0.64	0.65	0.64	0.50	0.47	<0.001
	Tokyo Station	Tokyo	1.15	0.85	0.84	0.82	0.56	0.44	<0.001
	Shinbashi Station	Tokyo	1.04	0.76	0.73	0.75	0.61	0.58	<0.001
	Shinagawa Station	Tokyo	0.88	0.70	0.69	0.68	0.54	0.46	<0.001
	Ueno Station	Tokyo	0.94	0.70	0.66	0.64	0.45	0.35	<0.001
	Ginza Station	Tokyo	1.71	1.02	1.01	0.91	0.65	0.53	<0.001
	Roppongi Station	Tokyo	1.09	0.87	0.87	0.87	0.69	0.77	<0.001
	Kichijoji Station	Tokyo	1.24	0.76	0.76	0.73	0.59	0.56	<0.001
	Motomachi-Chukagai Station	Kanagawa	0.94	0.80	0.91	0.94	0.73	0.62	<0.001
	Sakuragicho Station	Kanagawa	1.40	1.04	0.97	1.00	0.78	0.63	<0.001
	Kannai Station	Kanagawa	1.34	0.84	0.85	0.92	0.83	0.74	<0.001
	Hamamatsu Station	Shizuoka	–	–	–	–	–	–	–
	Sakae Station	Aichi	1.02	0.75	0.75	0.74	0.54	0.44	<0.001
	Fushimi Station	Aichi	1.18	0.83	0.88	0.87	0.66	0.58	<0.001
	Kawaramachi Station	Kyoto	0.94	0.57	0.56	0.56	0.42	0.39	<0.001
	Shinsaibashi Station	Osaka	1.18	1.02	1.05	1.02	0.72	0.72	<0.001
	Namba Station	Osaka	0.80	0.59	0.58	0.56	0.43	0.43	<0.001
	Kitashinchi Station	Osaka	1.38	0.97	0.95	0.97	0.76	0.68	<0.001
	Tamachi Station	Okayama	–	–	–	–	–	–	–
	Hatchobori Station	Hiroshima	–	–	–	–	–	–	–
	Kawaramachi Station	Kagawa	–	–	–	–	–	–	–
	Hakata Station	Fukuoka	0.75	0.68	0.76	0.66	0.56	0.50	<0.001

	Nakasu-kawabata Station	Fukuoka	0.94	1.02	1.12	1.18	0.95	0.74	<0.001
	Kanko-dori Station	Nagasaki	–	–	–	–	–	–	–
	Makishi Station	Okinawa	–	–	–	–	–	–	–
16:00–19:59 PM									
	Susukino Station	Hokkaido	–	–	–	–	–	–	–
	Odori Station	Hokkaido	–	–	–	–	–	–	–
	Central Hirosaki Station	Aomori	–	–	–	–	–	–	–
	Kotodai-Koen Station	Miyagi	–	–	–	–	–	–	–
	Tobu-Utsunomiya station	Tochigi	0.60	0.56	0.61	0.65	0.53	0.45	<0.001
	Chuo Maebashi Station	Gunma	–	–	–	–	–	–	–
	Shibuya Station	Tokyo	0.89	0.52	0.49	0.48	0.36	0.46	<0.001
	Shibuya Center Street	Tokyo	0.82	0.47	0.43	0.42	0.32	0.42	<0.001
	Harajuku Station	Tokyo	1.07	0.67	0.59	0.56	0.46	0.69	<0.001
	Shinjuku Kabukicho	Tokyo	0.74	0.55	0.53	0.54	0.40	0.41	<0.001
	Ikebukuro Station	Tokyo	0.87	0.57	0.58	0.57	0.43	0.47	<0.001
	Tokyo Station	Tokyo	0.90	0.68	0.65	0.64	0.45	0.39	<0.001
	Shinbashi Station	Tokyo	0.89	0.67	0.64	0.67	0.49	0.45	<0.001
	Shinagawa Station	Tokyo	0.70	0.58	0.58	0.59	0.47	0.44	<0.001
	Ueno Station	Tokyo	0.77	0.61	0.60	0.59	0.43	0.36	<0.001
	Ginza Station	Tokyo	1.20	0.82	0.79	0.74	0.53	0.41	<0.001
	Roppongi Station	Tokyo	1.02	0.82	0.77	0.79	0.62	0.57	<0.001
	Kichijoji Station	Tokyo	0.96	0.63	0.60	0.58	0.44	0.44	<0.001
	Motomachi-Chukagai Station	Kanagawa	0.90	0.67	0.70	0.80	0.59	0.55	<0.001
	Sakuragicho Station	Kanagawa	1.05	0.76	0.75	0.77	0.58	0.54	<0.001
	Kannai Station	Kanagawa	1.05	0.78	0.81	0.85	0.69	0.62	<0.001
	Hamamatsu Station	Shizuoka	–	–	–	–	–	–	–
	Sakae Station	Aichi	0.76	0.64	0.60	0.58	0.42	0.40	<0.001
	Fushimi Station	Aichi	0.96	0.69	0.72	0.68	0.45	0.46	<0.001
	Kawaramachi Station	Kyoto	0.70	0.42	0.48	0.47	0.35	0.39	<0.001
	Shinsaibashi Station	Osaka	0.93	0.81	0.85	0.83	0.60	0.59	<0.001
	Namba Station	Osaka	0.68	0.55	0.54	0.55	0.40	0.41	<0.001
	Kitashinchi Station	Osaka	0.99	0.73	0.74	0.75	0.55	0.56	<0.001
	Tamachi Station	Okayama	–	–	–	–	–	–	–
	Hatchobori Station	Hiroshima	–	–	–	–	–	–	–
	Kawaramachi Station	Kagawa	–	–	–	–	–	–	–
	Hakata Station	Fukuoka	0.61	0.59	0.62	0.55	0.43	0.45	<0.001
	Nakasu-kawabata Station	Fukuoka	0.72	0.84	0.85	0.85	0.61	0.62	<0.001
	Kanko-dori Station	Nagasaki	–	–	–	–	–	–	–
	Makishi Station	Okinawa	–	–	–	–	–	–	–
20:00–23:59 PM									
	Susukino Station	Hokkaido	–	–	–	–	–	–	–
	Odori Station	Hokkaido	–	–	–	–	–	–	–
	Central Hirosaki Station	Aomori	–	–	–	–	–	–	–
	Kotodai-Koen Station	Miyagi	–	–	–	–	–	–	–
	Tobu-Utsunomiya station	Tochigi	0.28	0.31	0.38	0.41	0.28	0.30	<0.001
	Chuo Maebashi Station	Gunma	–	–	–	–	–	–	–
	Shibuya Station	Tokyo	0.47	0.30	0.30	0.26	0.20	0.35	<0.001
	Shibuya Center Street	Tokyo	0.44	0.27	0.27	0.24	0.17	0.32	<0.001
	Harajuku Station	Tokyo	0.64	0.44	0.43	0.35	0.28	0.57	<0.001

Shinjuku Kabukicho	Tokyo	0.42	0.30	0.31	0.30	0.21	0.32	<0.001
Ikebukuro Station	Tokyo	0.47	0.31	0.31	0.28	0.21	0.30	<0.001
Tokyo Station	Tokyo	0.49	0.38	0.37	0.32	0.19	0.26	<0.001
Shinbashi Station	Tokyo	0.52	0.38	0.36	0.32	0.21	0.28	<0.001
Shinagawa Station	Tokyo	0.44	0.39	0.38	0.35	0.26	0.32	<0.001
Ueno Station	Tokyo	0.50	0.37	0.37	0.33	0.20	0.25	<0.001
Ginza Station	Tokyo	0.55	0.38	0.35	0.31	0.19	0.22	<0.001
Roppongi Station	Tokyo	0.58	0.50	0.41	0.41	0.29	0.39	<0.001
Kichijoji Station	Tokyo	0.46	0.33	0.36	0.30	0.22	0.36	<0.001
Motomachi-Chukagai Station	Kanagawa	0.44	0.39	0.38	0.54	0.44	0.43	<0.001
Sakuragicho Station	Kanagawa	0.57	0.41	0.41	0.38	0.32	0.37	<0.001
Kannai Station	Kanagawa	0.55	0.45	0.46	0.39	0.28	0.38	<0.001
Hamamatsu Station	Shizuoka	–	–	–	–	–	–	–
Sakae Station	Aichi	0.38	0.33	0.32	0.26	0.18	0.20	<0.001
Fushimi Station	Aichi	0.51	0.34	0.34	0.29	0.20	0.23	<0.001
Kawaramachi Station	Kyoto	0.34	0.19	0.22	0.23	0.17	0.27	<0.001
Shinsaibashi Station	Osaka	0.47	0.35	0.39	0.33	0.24	0.33	<0.001
Namba Station	Osaka	0.36	0.27	0.28	0.26	0.18	0.29	<0.001
Kitashinchi Station	Osaka	0.40	0.32	0.31	0.27	0.20	0.23	<0.001
Tamachi Station	Okayama	–	–	–	–	–	–	–
Hatchobori Station	Hiroshima	–	–	–	–	–	–	–
Kawaramachi Station	Kagawa	–	–	–	–	–	–	–
Hakata Station	Fukuoka	0.28	0.30	0.33	0.26	0.22	0.26	<0.001
Nakasu-kawabata Station	Fukuoka	0.32	0.34	0.34	0.30	0.23	0.31	<0.001
Kanko-dori Station	Nagasaki	–	–	–	–	–	–	–
Makishi Station	Okinawa	–	–	–	–	–	–	–

Table S4: P-values for testing zero cross-correlation across all maximum +10-day lags between human mobility and daily number of newly reported COVID-19 cases during the declaration of a state of emergency, using the Haugh-Box test, by age groups and time frames, and by the first and second declaration. The ratios of the rolling seven-day daily average of the total population to a baseline on January 16, 2020, was compared with daily number of newly reported COVID-19 cases during the declaration of a state of emergency.

				Age group					
Declaration	Time frame	Area	Prefecture	20s	30s	40s	50s	60s	≥ 70s
First									
	0:00−7:59 AM								
		Central Hirosaki Station	Aomori	0.84	0.12	0.42	0.16	0.42	0.08
		Chuo Maebashi Station	Gunma	0.66	<0.001	0.12	<0.001	<0.001	0.08
		Fushimi Station	Aichi	<0.001	<0.001	<0.001	<0.001	<0.001	<0.001
		Ginza Station	Tokyo	<0.001	<0.001	<0.001	<0.001	<0.001	<0.001
		Hakata Station	Fukuoka	<0.001	<0.001	<0.001	<0.001	<0.001	<0.001
		Hamamatsu Station	Shizuoka	<0.001	<0.01	0.10	<0.001	<0.001	1.00
		Harajuku Station	Tokyo	<0.001	<0.001	<0.01	<0.05	<0.001	<0.001
		Hatchobori Station	Hiroshima	<0.05	<0.001	<0.05	<0.001	<0.01	<0.01
		Ikebukuro Station	Tokyo	<0.001	<0.001	<0.001	<0.001	<0.001	<0.001
		Kanko-dori Station	Nagasaki	0.99	0.79	0.96	0.96	0.96	0.89
		Kannai Station	Kanagawa	<0.001	<0.001	<0.001	0.14	<0.001	<0.001
		Kawaramachi Station	Kagawa	0.78	0.92	1.00	0.99	0.98	<0.01
		Kawaramachi Station	Kyoto	0.31	0.19	<0.001	<0.001	<0.001	0.41
		Kichijoji Station	Tokyo	0.69	<0.001	<0.001	<0.001	<0.001	0.06
		Kitashinchi Station	Osaka	<0.001	<0.001	<0.001	<0.001	<0.001	<0.001
		Kotodai-Koen Station	Miyagi	0.77	0.86	0.59	0.59	0.73	0.90
		Makishi Station	Okinawa	<0.001	<0.001	<0.001	<0.001	<0.001	<0.001
		Motomachi-Chukagai Station	Kanagawa	<0.001	<0.001	<0.001	<0.001	<0.001	<0.001
		Nakasu-kawabata Station	Fukuoka	<0.001	<0.001	<0.001	<0.001	<0.001	<0.001
		Namba Station	Osaka	<0.001	<0.001	<0.001	<0.001	<0.001	<0.001
		Odori Station	Hokkaido	<0.001	<0.001	<0.01	<0.001	<0.001	<0.001
		Roppongi Station	Tokyo	1.00	<0.001	<0.001	<0.001	<0.001	1.00
		Sakae Station	Aichi	<0.001	<0.001	<0.001	<0.001	<0.001	<0.001
		Sakuragicho Station	Kanagawa	<0.001	<0.001	<0.01	<0.001	<0.01	0.42
		Shibuya Center Street	Tokyo	<0.001	<0.001	<0.001	<0.001	<0.001	<0.001
		Shibuya Station	Tokyo	<0.001	<0.001	<0.001	<0.001	<0.001	<0.001
		Shinagawa Station	Tokyo	<0.001	<0.001	<0.001	<0.001	<0.001	<0.001
		Shinbashi Station	Tokyo	0.12	<0.001	<0.001	<0.001	<0.001	<0.001
		Shinjuku Kabukicho	Tokyo	<0.01	<0.001	<0.001	<0.001	<0.001	<0.001
		Shinsaibashi Station	Osaka	<0.001	<0.001	<0.001	<0.001	<0.001	0.63
		Susukino Station	Hokkaido	<0.001	<0.001	<0.001	<0.001	<0.001	<0.001
		Tamachi Station	Okayama	0.96	0.24	0.56	0.58	0.38	0.54
		Tobu-Utsunomiya station	Tochigi	<0.05	0.99	<0.01	<0.01	<0.05	<0.01
		Tokyo Station	Tokyo	<0.05	<0.001	<0.001	<0.001	<0.001	<0.001
		Ueno Station	Tokyo	0.10	<0.001	<0.001	<0.001	<0.001	<0.001
	08:00−11:59 AM								
		Central Hirosaki Station	Aomori	0.31	0.71	0.44	0.53	0.70	0.23

	Chuo Maebashi Station	Gunma	0.13	<0.05	<0.001	<0.001	<0.001	<0.001
	Fushimi Station	Aichi	<0.001	<0.001	<0.001	<0.001	<0.001	<0.001
	Ginza Station	Tokyo	<0.001	<0.001	<0.001	<0.001	<0.001	<0.01
	Hakata Station	Fukuoka	<0.001	<0.001	<0.001	<0.001	<0.001	<0.001
	Hamamatsu Station	Shizuoka	<0.001	<0.001	<0.001	<0.001	<0.001	<0.001
	Harajuku Station	Tokyo	<0.001	<0.001	<0.001	<0.001	<0.001	<0.001
	Hatchobori Station	Hiroshima	<0.001	<0.001	<0.001	<0.001	<0.001	<0.001
	Ikebukuro Station	Tokyo	<0.05	<0.001	<0.001	<0.001	<0.001	<0.01
	Kanko-dori Station	Nagasaki	0.93	0.69	0.87	0.88	0.93	0.94
	Kannai Station	Kanagawa	<0.001	<0.001	<0.001	<0.001	<0.001	<0.001
	Kawaramachi Station	Kagawa	<0.01	<0.05	<0.001	<0.01	0.21	<0.001
	Kawaramachi Station	Kyoto	<0.001	<0.001	<0.001	<0.001	<0.05	<0.05
	Kichijoji Station	Tokyo	<0.05	<0.001	<0.001	<0.001	<0.001	<0.001
	Kitashinchi Station	Osaka	<0.001	<0.001	<0.001	<0.001	<0.001	<0.001
	Kotodai-Koen Station	Miyagi	0.71	0.46	0.41	0.43	0.64	0.47
	Makishi Station	Okinawa	<0.001	<0.001	<0.001	<0.001	0.18	<0.001
	Motomachi-Chukagai Station	Kanagawa	0.09	<0.001	<0.001	<0.001	<0.001	<0.001
	Nakasu-kawabata Station	Fukuoka	<0.001	<0.001	<0.001	<0.001	<0.001	<0.001
	Namba Station	Osaka	<0.001	<0.001	<0.001	<0.001	<0.001	<0.001
	Odori Station	Hokkaido	<0.001	<0.001	<0.001	<0.001	<0.001	<0.001
	Roppongi Station	Tokyo	<0.001	<0.001	<0.001	<0.001	<0.001	<0.05
	Sakae Station	Aichi	<0.001	<0.001	<0.001	<0.001	<0.001	<0.001
	Sakuragicho Station	Kanagawa	<0.001	<0.001	<0.05	<0.001	<0.05	<0.01
	Shibuya Center Street	Tokyo	<0.05	<0.001	<0.001	<0.001	<0.001	<0.01
	Shibuya Station	Tokyo	<0.01	<0.001	<0.001	<0.001	<0.001	<0.001
	Shinagawa Station	Tokyo	<0.001	<0.001	<0.001	<0.001	<0.001	<0.001
	Shinbashi Station	Tokyo	<0.001	<0.001	<0.001	<0.001	<0.001	<0.001
	Shinjuku Kabukicho	Tokyo	<0.001	<0.001	<0.001	<0.001	<0.001	<0.05
	Shinsaibashi Station	Osaka	<0.001	<0.001	<0.001	<0.001	<0.001	<0.001
	Susukino Station	Hokkaido	<0.001	<0.001	<0.001	<0.001	<0.001	<0.001
	Tamachi Station	Okayama	0.54	0.72	0.65	0.66	0.41	0.42
	Tobu-Utsunomiya station	Tochigi	0.95	<0.05	<0.01	<0.001	<0.01	<0.001
	Tokyo Station	Tokyo	<0.001	<0.001	<0.001	<0.001	<0.001	<0.001
	Ueno Station	Tokyo	<0.001	<0.001	<0.001	<0.001	<0.001	<0.001
12:00−15:59 PM								
	Central Hirosaki Station	Aomori	0.76	0.32	0.49	0.59	0.72	<0.05
	Chuo Maebashi Station	Gunma	0.30	<0.001	<0.001	<0.001	<0.001	<0.05
	Fushimi Station	Aichi	<0.001	<0.001	<0.001	<0.001	<0.001	<0.001
	Ginza Station	Tokyo	<0.05	<0.05	<0.001	<0.001	<0.001	<0.001
	Hakata Station	Fukuoka	<0.001	<0.001	<0.001	<0.001	<0.001	<0.001
	Hamamatsu Station	Shizuoka	<0.001	<0.001	<0.001	<0.001	<0.001	<0.001
	Harajuku Station	Tokyo	<0.001	<0.05	<0.001	<0.05	<0.001	<0.001
	Hatchobori Station	Hiroshima	<0.001	<0.001	<0.001	<0.001	<0.001	<0.01
	Ikebukuro Station	Tokyo	0.06	<0.05	<0.001	<0.05	<0.05	<0.01
	Kanko-dori Station	Nagasaki	0.61	0.71	0.73	0.49	0.65	0.90
	Kannai Station	Kanagawa	<0.001	<0.001	<0.001	<0.001	<0.001	<0.001
	Kawaramachi Station	Kagawa	<0.001	0.46	<0.001	<0.001	0.24	0.34
	Kawaramachi Station	Kyoto	<0.001	<0.05	0.19	0.75	0.10	0.46
	Kichijoji Station	Tokyo	<0.001	0.22	<0.001	<0.001	<0.001	<0.01

	Kitashinchi Station	Osaka	<0.001	<0.001	<0.001	<0.001	<0.001	<0.001
	Kotodai-Koen Station	Miyagi	0.46	0.49	0.39	0.50	0.67	0.51
	Makishi Station	Okinawa	0.53	<0.001	<0.001	<0.001	0.41	<0.001
	Motomachi-Chukagai Station	Kanagawa	0.87	0.93	<0.001	0.92	<0.001	0.83
	Nakasu-kawabata Station	Fukuoka	<0.001	<0.001	<0.001	<0.001	<0.001	<0.001
	Namba Station	Osaka	<0.05	<0.001	<0.001	<0.001	<0.001	<0.001
	Odori Station	Hokkaido	<0.001	<0.001	<0.001	<0.001	<0.001	<0.001
	Roppongi Station	Tokyo	<0.001	<0.01	<0.001	<0.001	<0.001	<0.001
	Sakae Station	Aichi	<0.001	<0.001	<0.001	<0.001	<0.001	<0.001
	Sakuragicho Station	Kanagawa	<0.01	0.37	0.75	0.71	0.33	<0.05
	Shibuya Center Street	Tokyo	<0.05	<0.01	<0.001	<0.001	<0.001	<0.05
	Shibuya Station	Tokyo	<0.05	<0.001	<0.001	<0.001	<0.001	<0.05
	Shinagawa Station	Tokyo	<0.001	<0.001	<0.001	<0.001	<0.001	<0.05
	Shinbashi Station	Tokyo	<0.001	<0.001	<0.001	<0.001	<0.001	<0.05
	Shinjuku Kabukicho	Tokyo	<0.001	<0.001	<0.01	<0.001	<0.001	<0.05
	Shinsaibashi Station	Osaka	<0.001	<0.001	<0.001	<0.001	<0.001	<0.001
	Susukino Station	Hokkaido	<0.001	<0.001	<0.001	<0.001	<0.001	<0.001
	Tamachi Station	Okayama	0.38	0.79	0.62	0.71	0.57	0.53
	Tobu-Utsunomiya station	Tochigi	0.93	<0.001	<0.01	<0.01	<0.001	<0.001
	Tokyo Station	Tokyo	<0.001	<0.001	<0.001	<0.001	<0.001	<0.001
	Ueno Station	Tokyo	<0.001	<0.05	<0.001	<0.05	<0.001	0.12
16:00−19:59 PM								
	Central Hirosaki Station	Aomori	0.33	0.51	0.34	0.54	0.41	0.62
	Chuo Maebashi Station	Gunma	<0.05	<0.001	<0.001	<0.001	<0.001	<0.05
	Fushimi Station	Aichi	<0.001	<0.001	<0.001	<0.001	<0.001	<0.001
	Ginza Station	Tokyo	<0.001	<0.001	<0.001	<0.001	<0.001	<0.001
	Hakata Station	Fukuoka	<0.001	<0.001	<0.001	<0.001	<0.001	<0.001
	Hamamatsu Station	Shizuoka	<0.001	<0.001	<0.001	<0.001	<0.001	<0.001
	Harajuku Station	Tokyo	0.16	<0.001	<0.001	<0.001	<0.001	<0.001
	Hatchobori Station	Hiroshima	<0.001	<0.001	<0.001	<0.001	<0.001	<0.001
	Ikebukuro Station	Tokyo	<0.01	<0.001	<0.001	<0.001	<0.001	<0.001
	Kanko-dori Station	Nagasaki	0.42	0.65	0.81	0.88	0.79	0.75
	Kannai Station	Kanagawa	<0.001	<0.001	<0.001	<0.001	<0.001	<0.001
	Kawaramachi Station	Kagawa	<0.001	0.08	0.06	<0.05	0.07	0.82
	Kawaramachi Station	Kyoto	<0.001	<0.05	<0.01	<0.01	<0.001	0.24
	Kichijoji Station	Tokyo	<0.01	0.24	0.13	<0.001	<0.05	<0.001
	Kitashinchi Station	Osaka	<0.001	<0.001	<0.001	<0.001	<0.001	<0.001
	Kotodai-Koen Station	Miyagi	0.45	0.56	0.34	0.49	0.54	0.48
	Makishi Station	Okinawa	<0.001	<0.001	<0.001	<0.01	<0.001	<0.001
	Motomachi-Chukagai Station	Kanagawa	0.33	<0.001	<0.001	<0.001	<0.001	<0.001
	Nakasu-kawabata Station	Fukuoka	<0.001	<0.001	<0.001	<0.001	<0.001	<0.001
	Namba Station	Osaka	<0.001	<0.001	<0.001	<0.001	<0.001	<0.001
	Odori Station	Hokkaido	<0.001	<0.001	<0.001	<0.001	<0.001	<0.001
	Roppongi Station	Tokyo	<0.001	<0.05	<0.01	<0.001	<0.001	<0.05
	Sakae Station	Aichi	<0.001	<0.001	<0.001	<0.001	<0.001	<0.001
	Sakuragicho Station	Kanagawa	<0.001	<0.001	<0.001	<0.001	<0.001	0.97
	Shibuya Center Street	Tokyo	0.11	<0.001	<0.001	<0.001	<0.001	<0.001
	Shibuya Station	Tokyo	0.11	<0.001	<0.001	<0.001	<0.001	<0.001
	Shinagawa Station	Tokyo	<0.001	<0.001	<0.001	<0.001	<0.001	<0.001

		Shinbashi Station	Tokyo	<0.001	<0.001	<0.001	<0.001	<0.001	<0.05
		Shinjuku Kabukicho	Tokyo	<0.05	<0.001	<0.001	<0.001	<0.001	<0.001
		Shinsaibashi Station	Osaka	<0.001	<0.001	<0.001	<0.001	<0.001	<0.001
		Susukino Station	Hokkaido	<0.01	<0.001	<0.001	<0.001	<0.001	<0.001
		Tamachi Station	Okayama	0.64	0.58	0.58	0.58	0.56	0.37
		Tobu-Utsunomiya station	Tochigi	<0.001	<0.05	<0.001	<0.01	<0.001	<0.001
		Tokyo Station	Tokyo	<0.001	<0.001	<0.001	<0.001	<0.001	<0.001
		Ueno Station	Tokyo	<0.001	<0.001	<0.001	<0.001	<0.001	<0.05
	20:00–23:59 PM								
		Central Hirosaki Station	Aomori	0.84	0.21	0.22	0.40	0.44	0.60
		Chuo Maebashi Station	Gunma	0.10	<0.001	<0.01	<0.001	<0.001	<0.01
		Fushimi Station	Aichi	<0.001	<0.05	<0.001	<0.001	<0.001	<0.001
		Ginza Station	Tokyo	<0.001	<0.001	<0.001	<0.001	<0.001	<0.001
		Hakata Station	Fukuoka	<0.001	<0.001	<0.001	<0.001	<0.001	<0.001
		Hamamatsu Station	Shizuoka	<0.001	<0.001	<0.001	<0.001	<0.001	<0.001
		Harajuku Station	Tokyo	<0.001	<0.001	<0.001	<0.001	<0.001	<0.001
		Hatchobori Station	Hiroshima	<0.001	<0.001	<0.001	<0.001	<0.001	<0.001
		Ikebukuro Station	Tokyo	<0.001	<0.001	<0.001	<0.001	<0.001	<0.001
		Kanko-dori Station	Nagasaki	0.90	0.37	0.33	0.48	0.56	0.33
		Kannai Station	Kanagawa	<0.001	<0.001	<0.001	<0.001	<0.001	<0.001
		Kawaramachi Station	Kagawa	<0.001	0.95	0.77	0.19	0.60	0.96
		Kawaramachi Station	Kyoto	0.05	0.77	0.08	<0.05	<0.001	<0.001
		Kichijoji Station	Tokyo	<0.001	<0.01	<0.001	<0.001	0.06	0.07
		Kitashinchi Station	Osaka	<0.001	<0.001	<0.001	<0.001	<0.001	<0.001
		Kotodai-Koen Station	Miyagi	0.15	0.55	0.32	0.56	0.32	0.98
		Makishi Station	Okinawa	<0.001	<0.001	<0.001	<0.001	<0.001	<0.001
		Motomachi-Chukagai Station	Kanagawa	0.34	<0.001	<0.001	<0.001	<0.001	<0.001
		Nakasu-kawabata Station	Fukuoka	<0.05	<0.001	<0.001	<0.001	<0.001	<0.001
		Namba Station	Osaka	<0.001	<0.001	<0.001	<0.001	<0.001	<0.001
		Odori Station	Hokkaido	<0.001	<0.001	<0.001	<0.001	<0.001	<0.001
		Roppongi Station	Tokyo	0.26	<0.01	<0.01	<0.001	<0.001	0.40
		Sakae Station	Aichi	<0.001	<0.01	<0.001	<0.001	<0.001	<0.001
		Sakuragicho Station	Kanagawa	<0.001	0.46	0.93	<0.001	<0.001	<0.01
		Shibuya Center Street	Tokyo	<0.05	<0.001	<0.001	<0.001	<0.001	<0.001
		Shibuya Station	Tokyo	<0.01	<0.001	<0.001	<0.001	<0.001	<0.001
		Shinagawa Station	Tokyo	<0.001	<0.001	<0.001	<0.001	<0.001	<0.001
		Shinbashi Station	Tokyo	<0.05	<0.001	<0.001	<0.001	<0.001	<0.001
		Shinjuku Kabukicho	Tokyo	<0.05	<0.001	<0.001	<0.001	<0.001	<0.001
		Shinsaibashi Station	Osaka	<0.001	<0.001	<0.001	<0.001	<0.001	<0.001
		Susukino Station	Hokkaido	<0.001	<0.001	<0.001	<0.001	<0.001	<0.001
		Tamachi Station	Okayama	0.44	0.19	0.54	0.85	0.59	0.28
		Tobu-Utsunomiya station	Tochigi	<0.001	0.11	<0.001	0.10	0.26	<0.001
		Tokyo Station	Tokyo	<0.001	<0.001	<0.001	<0.001	<0.001	<0.01
		Ueno Station	Tokyo	<0.001	<0.001	<0.001	<0.001	<0.001	<0.001
Second									
	0:00–7:59 AM								
		Susukino Station	Hokkaido	–	–	–	–	–	–
		Odori Station	Hokkaido	–	–	–	–	–	–
		Central Hirosaki Station	Aomori	–	–	–	–	–	–

	Kotodai-Koen Station	Miyagi	–	–	–	–	–	–
	Tobu-Utsunomiya station	Tochigi	<0.001	<0.001	<0.001	0.15	<0.01	<0.05
	Chuo Maebashi Station	Gunma	–	–	–	–	–	–
	Shibuya Station	Tokyo	<0.001	<0.001	<0.001	<0.001	<0.001	<0.001
	Shibuya Center Street	Tokyo	<0.001	<0.001	<0.001	<0.001	<0.001	<0.01
	Harajuku Station	Tokyo	<0.001	0.12	1.00	<0.001	<0.001	<0.05
	Shinjuku Kabukicho	Tokyo	0.50	<0.001	<0.001	0.92	<0.05	<0.01
	Ikebukuro Station	Tokyo	<0.01	<0.001	0.91	0.39	<0.001	<0.001
	Tokyo Station	Tokyo	<0.001	0.57	0.88	0.90	0.29	<0.05
	Shinbashi Station	Tokyo	<0.001	0.22	1.00	0.98	0.31	<0.001
	Shinagawa Station	Tokyo	0.05	<0.001	<0.001	<0.001	<0.01	0.71
	Ueno Station	Tokyo	0.87	0.42	<0.001	1.00	<0.001	<0.001
	Ginza Station	Tokyo	<0.001	<0.05	0.43	1.00	0.82	0.92
	Roppongi Station	Tokyo	<0.001	<0.001	<0.01	<0.01	0.84	<0.001
	Kichijoji Station	Tokyo	<0.001	<0.001	0.92	<0.05	<0.001	0.54
	Motomachi-Chukagai Station	Kanagawa	<0.001	<0.001	<0.001	<0.01	<0.001	<0.05
	Sakuragicho Station	Kanagawa	1.00	<0.001	0.30	1.00	<0.001	1.00
	Kannai Station	Kanagawa	<0.001	0.26	<0.001	<0.001	<0.01	0.57
	Hamamatsu Station	Shizuoka	–	–	–	–	–	–
	Sakae Station	Aichi	0.44	0.57	0.28	0.69	<0.01	0.94
	Fushimi Station	Aichi	0.47	<0.001	<0.001	<0.001	<0.001	0.18
	Kawaramachi Station	Kyoto	0.93	<0.001	0.71	<0.05	<0.001	<0.001
	Shinsaibashi Station	Osaka	0.88	<0.05	<0.001	<0.01	0.17	<0.001
	Namba Station	Osaka	0.57	<0.01	<0.001	<0.001	<0.001	<0.001
	Kitashinchi Station	Osaka	0.28	<0.001	<0.001	<0.001	<0.05	<0.001
	Tamachi Station	Okayama	–	–	–	–	–	–
	Hatchobori Station	Hiroshima	–	–	–	–	–	–
	Kawaramachi Station	Kagawa	–	–	–	–	–	–
	Hakata Station	Fukuoka	<0.001	<0.001	<0.01	<0.001	<0.001	<0.001
	Nakasu-kawabata Station	Fukuoka	<0.001	<0.001	<0.001	<0.001	<0.001	<0.001
	Kanko-dori Station	Nagasaki	–	–	–	–	–	–
	Makishi Station	Okinawa	–	–	–	–	–	–
08:00–11:59 AM								
	Susukino Station	Hokkaido	–	–	–	–	–	–
	Odori Station	Hokkaido	–	–	–	–	–	–
	Central Hirosaki Station	Aomori	–	–	–	–	–	–
	Kotodai-Koen Station	Miyagi	–	–	–	–	–	–
	Tobu-Utsunomiya station	Tochigi	0.70	<0.001	<0.001	<0.05	0.25	<0.05
	Chuo Maebashi Station	Gunma	–	–	–	–	–	–
	Shibuya Station	Tokyo	<0.001	<0.001	0.98	0.62	1.00	<0.001
	Shibuya Center Street	Tokyo	<0.001	<0.001	0.95	0.86	1.00	<0.001
	Harajuku Station	Tokyo	<0.001	<0.001	0.83	0.96	0.05	<0.001
	Shinjuku Kabukicho	Tokyo	<0.001	<0.001	<0.001	<0.01	0.45	<0.001
	Ikebukuro Station	Tokyo	0.99	<0.001	0.14	0.32	1.00	<0.001
	Tokyo Station	Tokyo	0.97	0.33	0.92	1.00	0.98	0.92
	Shinbashi Station	Tokyo	<0.001	0.99	0.98	1.00	0.88	<0.001
	Shinagawa Station	Tokyo	<0.001	0.90	1.00	1.00	0.48	<0.001
	Ueno Station	Tokyo	<0.001	<0.05	<0.05	<0.001	0.96	<0.001
	Ginza Station	Tokyo	<0.001	0.07	1.00	0.61	<0.05	<0.001

	Roppongi Station	Tokyo	<0.001	0.40	<0.001	<0.01	<0.001	1.00
	Kichijoji Station	Tokyo	1.00	0.20	0.94	<0.001	<0.01	<0.001
	Motomachi-Chukagai Station	Kanagawa	<0.001	<0.001	<0.001	<0.05	<0.001	0.26
	Sakuragicho Station	Kanagawa	<0.001	<0.001	0.25	1.00	<0.001	<0.001
	Kannai Station	Kanagawa	<0.001	<0.001	<0.001	<0.001	0.93	<0.001
	Hamamatsu Station	Shizuoka	–	–	–	–	–	–
	Sakae Station	Aichi	<0.001	<0.001	<0.001	<0.001	<0.001	0.91
	Fushimi Station	Aichi	<0.01	<0.001	<0.001	<0.01	<0.05	0.96
	Kawaramachi Station	Kyoto	<0.001	<0.001	<0.001	<0.001	<0.001	<0.001
	Shinsaibashi Station	Osaka	<0.001	<0.001	<0.001	<0.001	<0.001	<0.001
	Namba Station	Osaka	<0.001	<0.001	<0.001	<0.001	<0.001	<0.001
	Kitashinchi Station	Osaka	<0.001	<0.001	<0.001	<0.01	<0.001	<0.001
	Tamachi Station	Okayama	–	–	–	–	–	–
	Hatchobori Station	Hiroshima	–	–	–	–	–	–
	Kawaramachi Station	Kagawa	–	–	–	–	–	–
	Hakata Station	Fukuoka	0.90	<0.001	0.05	<0.05	<0.001	<0.01
	Nakasu-kawabata Station	Fukuoka	<0.001	<0.01	<0.001	<0.01	<0.001	<0.05
	Kanko-dori Station	Nagasaki	–	–	–	–	–	–
	Makishi Station	Okinawa	–	–	–	–	–	–
12:00–15:59 PM								
	Susukino Station	Hokkaido	–	–	–	–	–	–
	Odori Station	Hokkaido	–	–	–	–	–	–
	Central Hirosaki Station	Aomori	–	–	–	–	–	–
	Kotodai-Koen Station	Miyagi	–	–	–	–	–	–
	Tobu-Utsunomiya station	Tochigi	<0.01	0.77	0.94	<0.05	<0.01	0.05
	Chuo Maebashi Station	Gunma	–	–	–	–	–	–
	Shibuya Station	Tokyo	<0.001	<0.001	<0.001	<0.001	<0.001	<0.001
	Shibuya Center Street	Tokyo	<0.001	<0.001	<0.001	<0.001	<0.001	<0.001
	Harajuku Station	Tokyo	<0.001	<0.001	0.80	<0.001	<0.001	<0.001
	Shinjuku Kabukicho	Tokyo	<0.001	<0.001	<0.001	<0.001	0.26	<0.001
	Ikebukuro Station	Tokyo	<0.001	<0.001	<0.001	<0.001	0.90	<0.001
	Tokyo Station	Tokyo	<0.001	0.22	0.99	1.00	0.98	<0.001
	Shinbashi Station	Tokyo	<0.001	0.80	0.71	1.00	<0.01	<0.001
	Shinagawa Station	Tokyo	<0.01	0.61	1.00	0.97	<0.001	<0.001
	Ueno Station	Tokyo	<0.001	<0.001	<0.001	<0.001	<0.001	<0.001
	Ginza Station	Tokyo	<0.001	<0.05	0.96	0.67	<0.001	<0.001
	Roppongi Station	Tokyo	<0.001	0.31	0.95	0.07	0.13	<0.001
	Kichijoji Station	Tokyo	<0.001	<0.001	<0.001	1.00	<0.05	<0.001
	Motomachi-Chukagai Station	Kanagawa	<0.001	<0.05	<0.001	<0.001	<0.001	0.89
	Sakuragicho Station	Kanagawa	<0.001	<0.001	<0.001	1.00	0.28	<0.001
	Kannai Station	Kanagawa	<0.001	<0.001	<0.01	<0.001	0.72	<0.001
	Hamamatsu Station	Shizuoka	–	–	–	–	–	–
	Sakae Station	Aichi	<0.05	<0.001	<0.001	<0.001	<0.01	0.33
	Fushimi Station	Aichi	<0.001	<0.001	<0.001	<0.001	<0.001	0.79
	Kawaramachi Station	Kyoto	<0.001	0.30	<0.05	<0.01	0.70	<0.001
	Shinsaibashi Station	Osaka	0.51	<0.001	<0.001	<0.001	<0.001	0.77
	Namba Station	Osaka	0.46	<0.001	<0.001	<0.001	<0.001	0.11
	Kitashinchi Station	Osaka	<0.05	<0.001	<0.001	<0.001	<0.001	0.97
	Tamachi Station	Okayama	–	–	–	–	–	–

	Hatchobori Station	Hiroshima	–	–	–	–	–	–
	Kawaramachi Station	Kagawa	–	–	–	–	–	–
	Hakata Station	Fukuoka	<0.01	<0.001	<0.01	0.65	0.17	0.97
	Nakasu-kawabata Station	Fukuoka	0.07	<0.001	<0.001	<0.01	0.90	<0.001
	Kanko-dori Station	Nagasaki	–	–	–	–	–	–
	Makishi Station	Okinawa	–	–	–	–	–	–
16:00–19:59 PM								
	Susukino Station	Hokkaido	–	–	–	–	–	–
	Odori Station	Hokkaido	–	–	–	–	–	–
	Central Hirosaki Station	Aomori	–	–	–	–	–	–
	Kotodai-Koen Station	Miyagi	–	–	–	–	–	–
	Tobu-Utsunomiya station	Tochigi	0.07	0.05	<0.05	<0.001	<0.001	0.29
	Chuo Maebashi Station	Gunma	–	–	–	–	–	–
	Shibuya Station	Tokyo	<0.001	<0.001	<0.001	<0.001	<0.001	<0.001
	Shibuya Center Street	Tokyo	<0.001	<0.001	<0.001	<0.001	<0.001	<0.001
	Harajuku Station	Tokyo	<0.001	<0.001	<0.001	<0.001	<0.001	<0.001
	Shinjuku Kabukicho	Tokyo	<0.001	<0.001	<0.001	<0.001	<0.001	<0.001
	Ikebukuro Station	Tokyo	<0.001	<0.001	<0.001	<0.001	<0.001	0.09
	Tokyo Station	Tokyo	<0.01	<0.05	0.28	0.99	1.00	<0.001
	Shinbashi Station	Tokyo	<0.001	0.81	1.00	1.00	0.31	<0.001
	Shinagawa Station	Tokyo	0.39	0.62	0.89	0.89	0.88	<0.001
	Ueno Station	Tokyo	<0.01	<0.01	<0.001	1.00	0.98	<0.001
	Ginza Station	Tokyo	<0.001	<0.001	<0.001	0.05	0.99	<0.001
	Roppongi Station	Tokyo	<0.001	0.94	0.10	<0.01	0.91	<0.001
	Kichijoji Station	Tokyo	<0.001	<0.001	<0.001	<0.001	<0.001	<0.001
	Motomachi-Chukagai Station	Kanagawa	<0.001	0.90	<0.001	<0.001	<0.001	<0.01
	Sakuragicho Station	Kanagawa	<0.001	<0.001	<0.001	<0.001	<0.001	<0.001
	Kannai Station	Kanagawa	<0.001	<0.001	<0.001	<0.001	0.96	<0.001
	Hamamatsu Station	Shizuoka	–	–	–	–	–	–
	Sakae Station	Aichi	0.93	<0.001	<0.001	<0.01	<0.001	0.86
	Fushimi Station	Aichi	<0.001	<0.001	<0.001	<0.001	<0.001	<0.01
	Kawaramachi Station	Kyoto	<0.001	<0.001	<0.001	<0.001	<0.001	<0.01
	Shinsaibashi Station	Osaka	<0.001	<0.001	<0.001	<0.001	<0.001	1.00
	Namba Station	Osaka	0.88	<0.001	<0.001	<0.001	<0.001	0.92
	Kitashinchi Station	Osaka	<0.05	<0.001	<0.001	<0.001	<0.001	0.21
	Tamachi Station	Okayama	–	–	–	–	–	–
	Hatchobori Station	Hiroshima	–	–	–	–	–	–
	Kawaramachi Station	Kagawa	–	–	–	–	–	–
	Hakata Station	Fukuoka	1.00	<0.001	<0.01	<0.001	<0.01	<0.05
	Nakasu-kawabata Station	Fukuoka	<0.001	<0.001	<0.001	<0.001	<0.001	0.08
	Kanko-dori Station	Nagasaki	–	–	–	–	–	–
	Makishi Station	Okinawa	–	–	–	–	–	–
20:00–23:59 PM								
	Susukino Station	Hokkaido	–	–	–	–	–	–
	Odori Station	Hokkaido	–	–	–	–	–	–
	Central Hirosaki Station	Aomori	–	–	–	–	–	–
	Kotodai-Koen Station	Miyagi	–	–	–	–	–	–
	Tobu-Utsunomiya station	Tochigi	<0.001	<0.001	<0.001	0.93	<0.001	1.00
	Chuo Maebashi Station	Gunma	–	–	–	–	–	–

	Shibuya Station	Tokyo	<0.001	0.38	0.97	0.58	0.96	<0.001
	Shibuya Center Street	Tokyo	<0.001	0.91	1.00	<0.05	1.00	<0.001
	Harajuku Station	Tokyo	<0.001	0.99	1.00	0.94	<0.001	<0.001
	Shinjuku Kabukicho	Tokyo	<0.001	<0.001	0.45	1.00	<0.001	<0.001
	Ikebukuro Station	Tokyo	<0.001	<0.001	0.13	0.98	0.06	<0.001
	Tokyo Station	Tokyo	<0.001	<0.001	<0.001	<0.001	<0.001	<0.001
	Shinbashi Station	Tokyo	<0.001	<0.001	<0.001	<0.001	<0.001	<0.001
	Shinagawa Station	Tokyo	<0.001	0.27	<0.001	<0.001	<0.001	<0.001
	Ueno Station	Tokyo	<0.001	<0.001	<0.001	<0.001	<0.001	<0.001
	Ginza Station	Tokyo	<0.001	<0.001	<0.001	<0.001	0.24	<0.001
	Roppongi Station	Tokyo	<0.001	<0.001	<0.001	<0.001	0.37	<0.001
	Kichijoji Station	Tokyo	<0.001	<0.001	<0.001	<0.001	<0.001	<0.001
	Motomachi-Chukagai Station	Kanagawa	<0.001	<0.001	0.16	<0.001	<0.001	<0.01
	Sakuragicho Station	Kanagawa	<0.001	1.00	<0.01	0.41	<0.001	<0.001
	Kannai Station	Kanagawa	<0.001	0.64	<0.001	0.83	0.70	<0.001
	Hamamatsu Station	Shizuoka	–	–	–	–	–	–
	Sakae Station	Aichi	<0.05	0.20	<0.001	<0.01	<0.001	<0.01
	Fushimi Station	Aichi	<0.05	<0.001	<0.001	<0.001	<0.05	<0.001
	Kawaramachi Station	Kyoto	0.05	<0.001	0.23	<0.001	<0.001	0.13
	Shinsaibashi Station	Osaka	<0.01	<0.001	<0.001	<0.001	<0.001	<0.001
	Namba Station	Osaka	<0.05	<0.001	<0.001	<0.001	<0.001	0.36
	Kitashinchi Station	Osaka	0.93	<0.01	<0.001	<0.001	<0.001	0.26
	Tamachi Station	Okayama	–	–	–	–	–	–
	Hatchobori Station	Hiroshima	–	–	–	–	–	–
	Kawaramachi Station	Kagawa	–	–	–	–	–	–
	Hakata Station	Fukuoka	<0.001	<0.001	0.85	1.00	<0.001	<0.05
	Nakasu-kawabata Station	Fukuoka	<0.01	<0.001	<0.001	<0.001	<0.001	<0.01
	Kanko-dori Station	Nagasaki	–	–	–	–	–	–
	Makishi Station	Okinawa	–	–	–	–	–	–

Figure S1: A map showing the location of the 35 areas. For simplicity of the map, all areas in the same prefecture are combined into the same plot. This map is based on the Digital Map 25000 (Map Image) published by Geospatial Information Authority of Japan (Approval No.603FY2017 information usage <http://www.gsi.go.jp>).

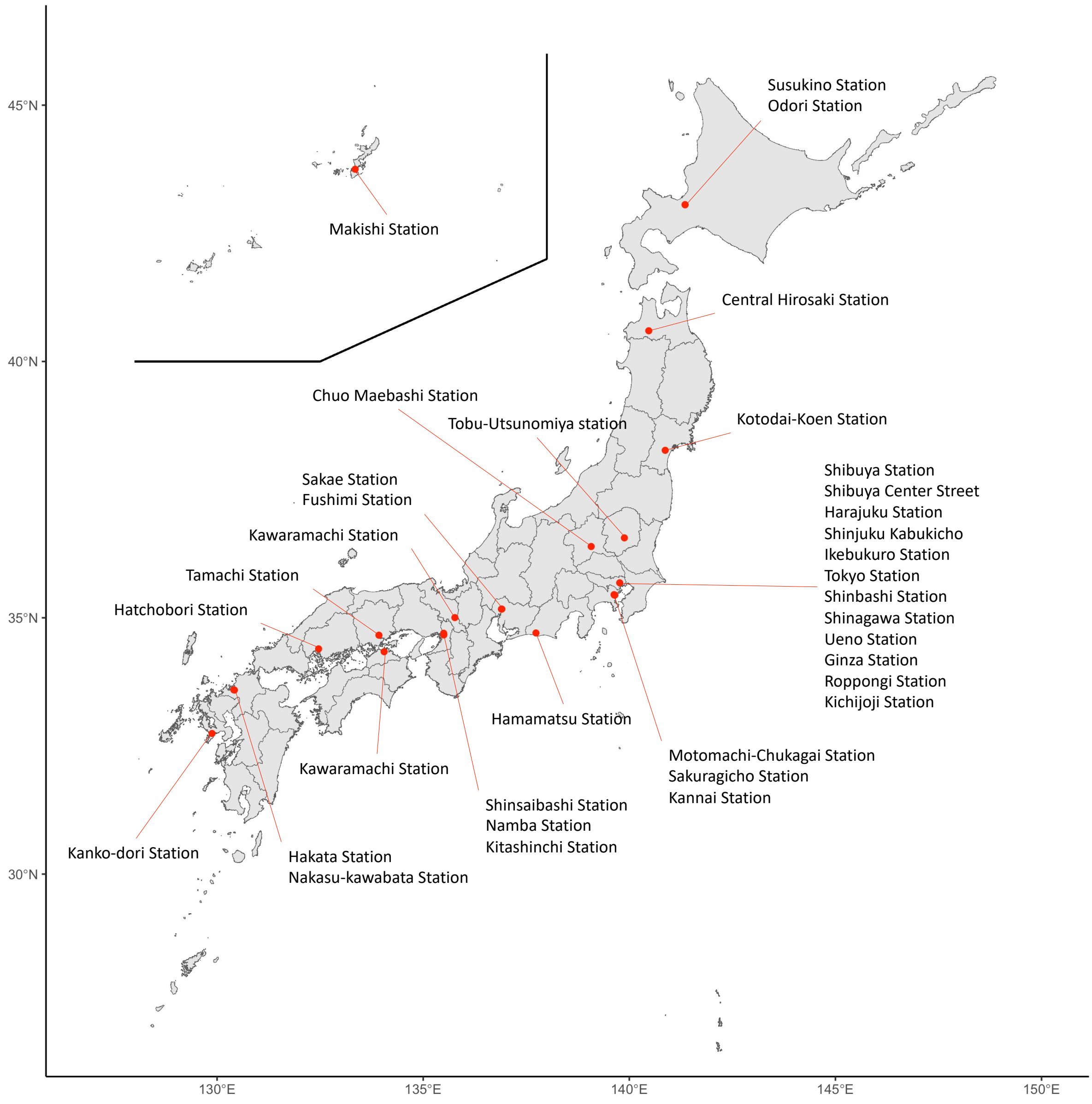

Figure S2: Temporal trends in human mobility by age groups and time frames in other areas. The top five graphs show the ratio of the rolling seven-day daily average of the total population based on different time frames, and the last one is a graph of the daily number of newly reported COVID-19 cases. Gray areas indicate a state of emergency.

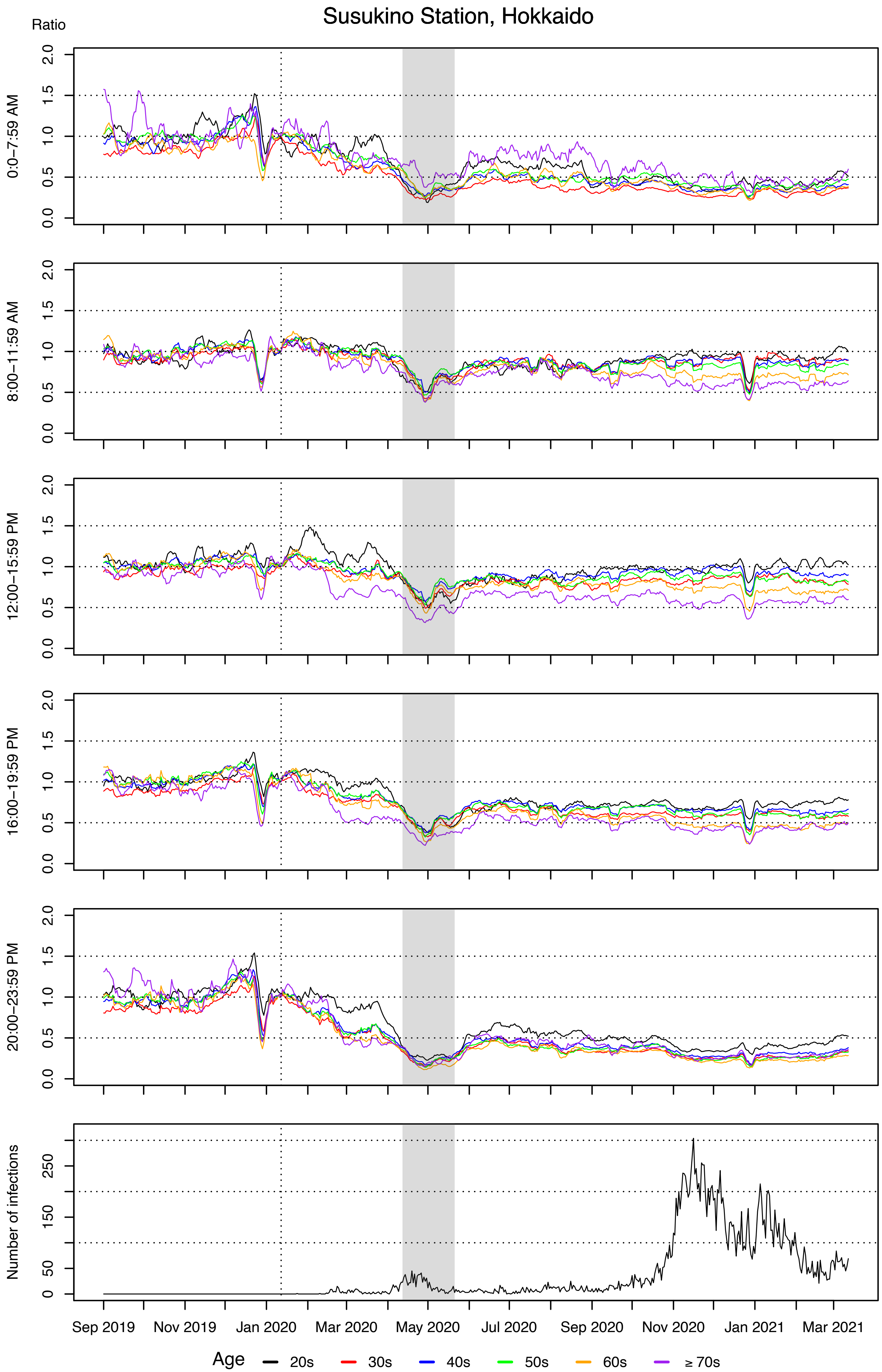

Susukino Station, Hokkaido
Ratio
0:0−7:59 AM
8:00−11:59 AM
12:00−15:59 PM
16:00−19:59 PM
20:00−23:59 PM
Number of infections
0.0
0.5
1.0
1.5
2.0
0
50
150
250
Sep 2019
Nov 2019
Jan 2020
Mar 2020
May 2020
Jul 2020
Sep 2020
Nov 2020
Jan 2021
Mar 2021
Age
20s
30s
40s
50s
60s
≥ 70s

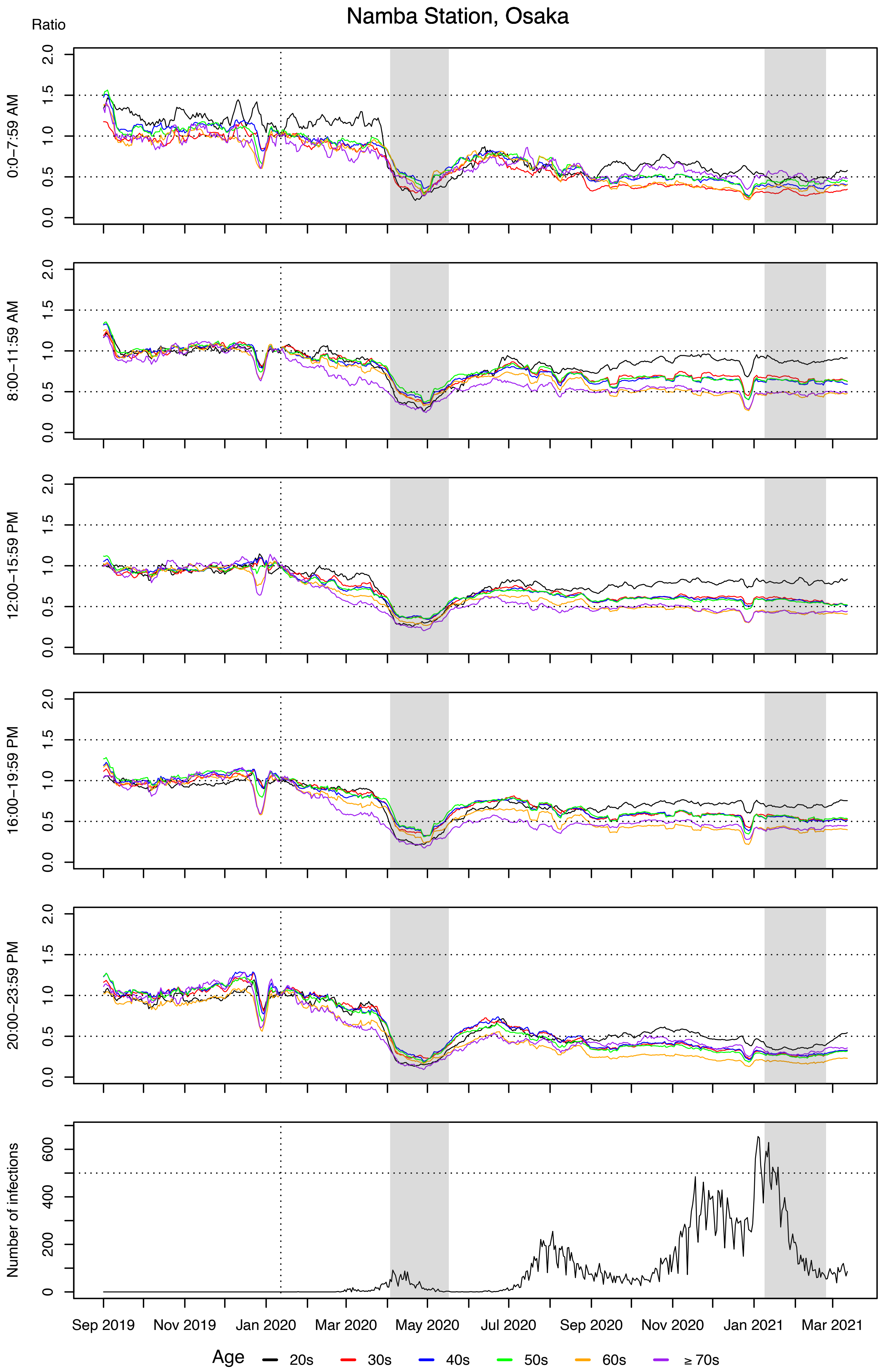
Namba Station, Osaka
Ratio
0:0−7:59 AM
8:00−11:59 AM
12:00−15:59 PM
16:00−19:59 PM
20:00−23:59 PM
Number of infections
0.0
0.5
1.0
1.5
2.0
0
200
400
600
Sep 2019
Nov 2019
Jan 2020
Mar 2020
May 2020
Jul 2020
Sep 2020
Nov 2020
Jan 2021
Mar 2021
Age
20s
30s
40s
50s
60s
≥ 70s

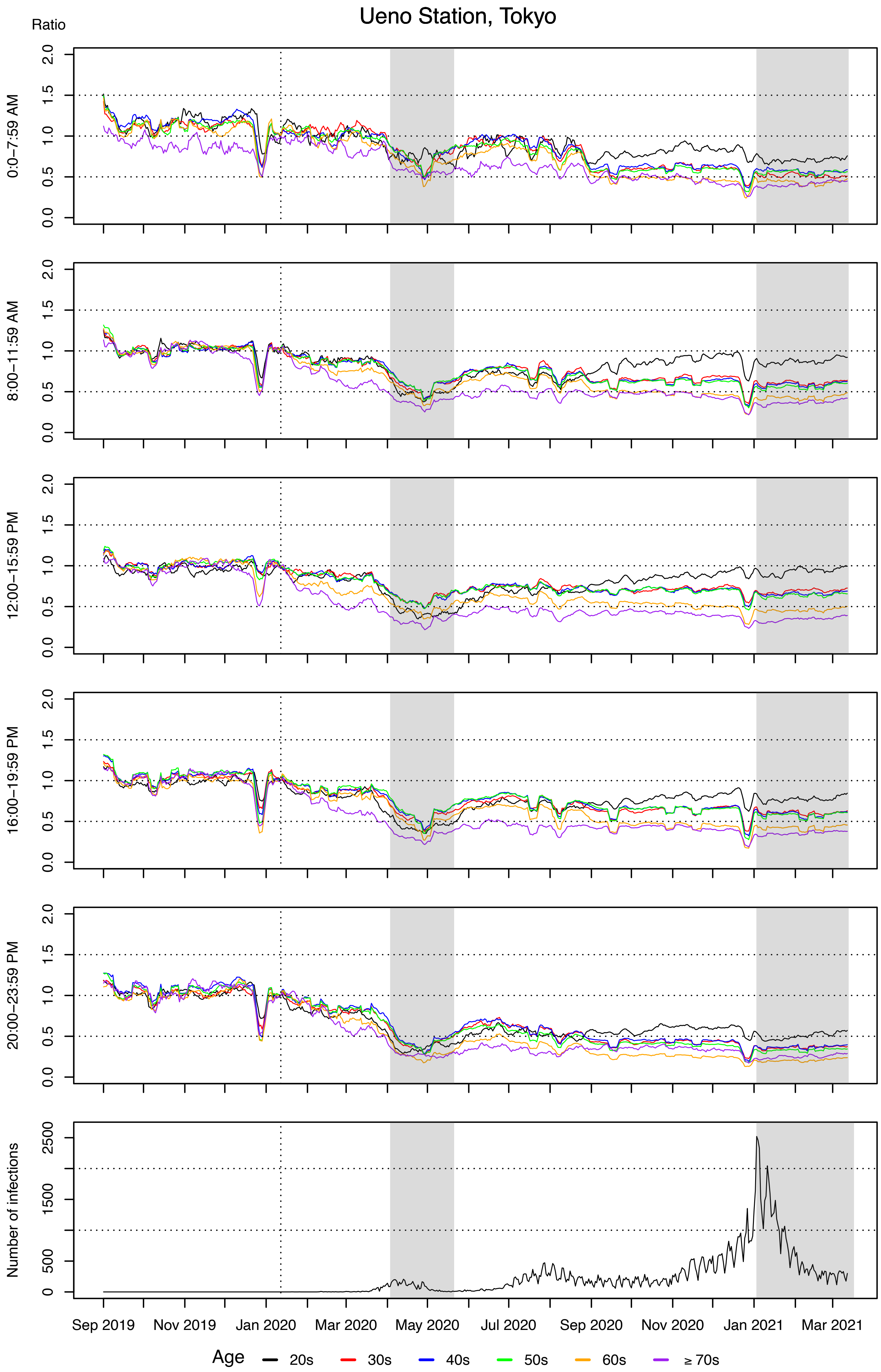

Ueno Station, Tokyo
Ratio
0:0−7:59 AM
8:00−11:59 AM
12:00−15:59 PM
16:00−19:59 PM
20:00−23:59 PM
Number of infections
0.0
0.5
1.0
1.5
2.0
0
500
1500
2500
Sep 2019
Nov 2019
Jan 2020
Mar 2020
May 2020
Jul 2020
Sep 2020
Nov 2020
Jan 2021
Mar 2021
Age
20s
30s
40s
50s
60s
≥ 70s

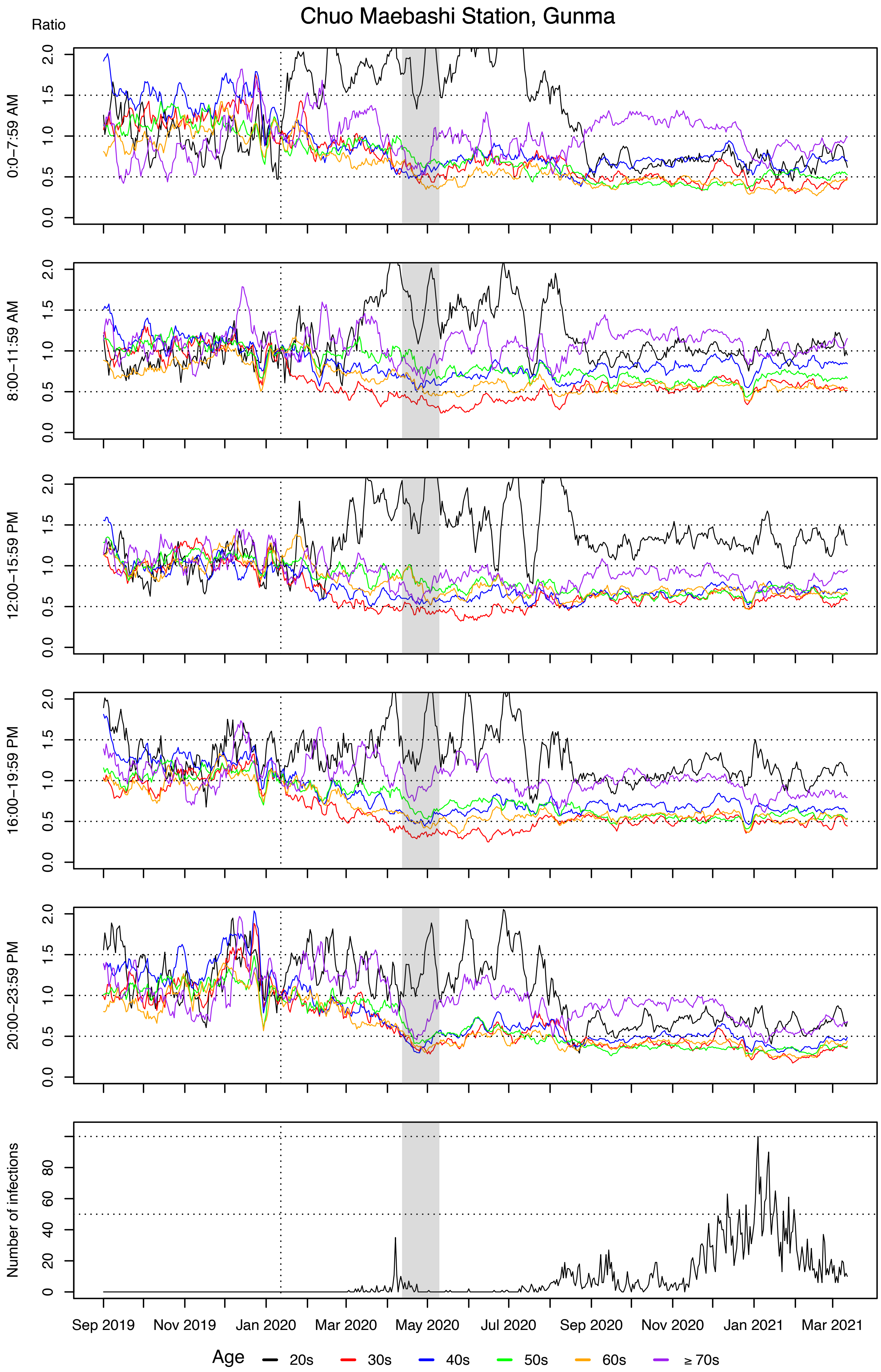
Chuo Maebashi Station, Gunma
Ratio
0:0−7:59 AM
8:00−11:59 AM
12:00−15:59 PM
16:00−19:59 PM
20:00−23:59 PM
Number of infections
Sep 2019
Nov 2019
Jan 2020
Mar 2020
May 2020
Jul 2020
Sep 2020
Nov 2020
Jan 2021
Mar 2021
Age
20s
30s
40s
50s
60s
≥ 70s

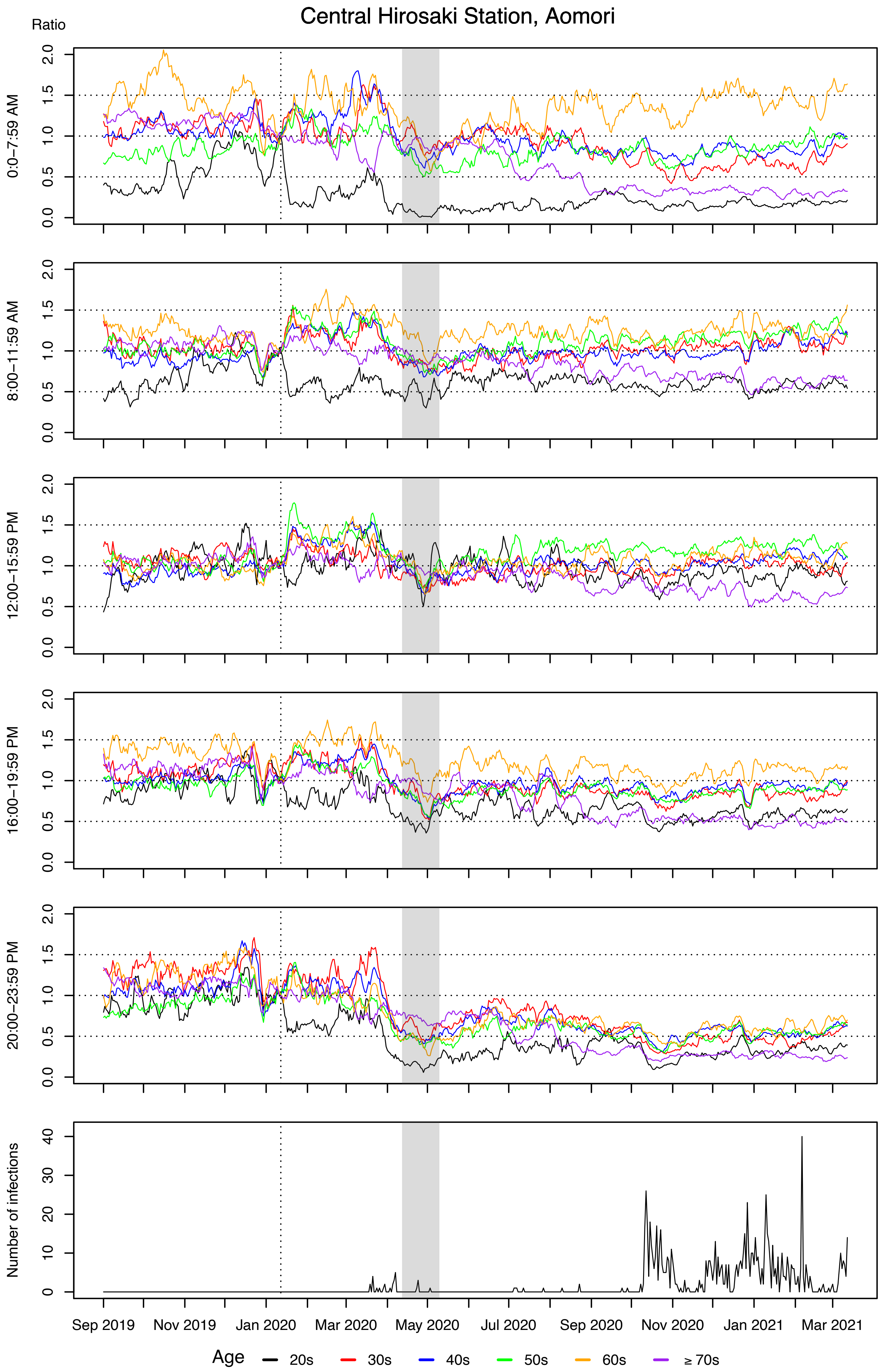

Central Hirosaki Station, Aomori
Ratio
0:0−7:59 AM
8:00−11:59 AM
12:00−15:59 PM
16:00−19:59 PM
20:00−23:59 PM
Number of infections
0.0
0.5
1.0
1.5
2.0
0
10
20
30
40
Sep 2019
Nov 2019
Jan 2020
Mar 2020
May 2020
Jul 2020
Sep 2020
Nov 2020
Jan 2021
Mar 2021
Age
20s
30s
40s
50s
60s
≥ 70s

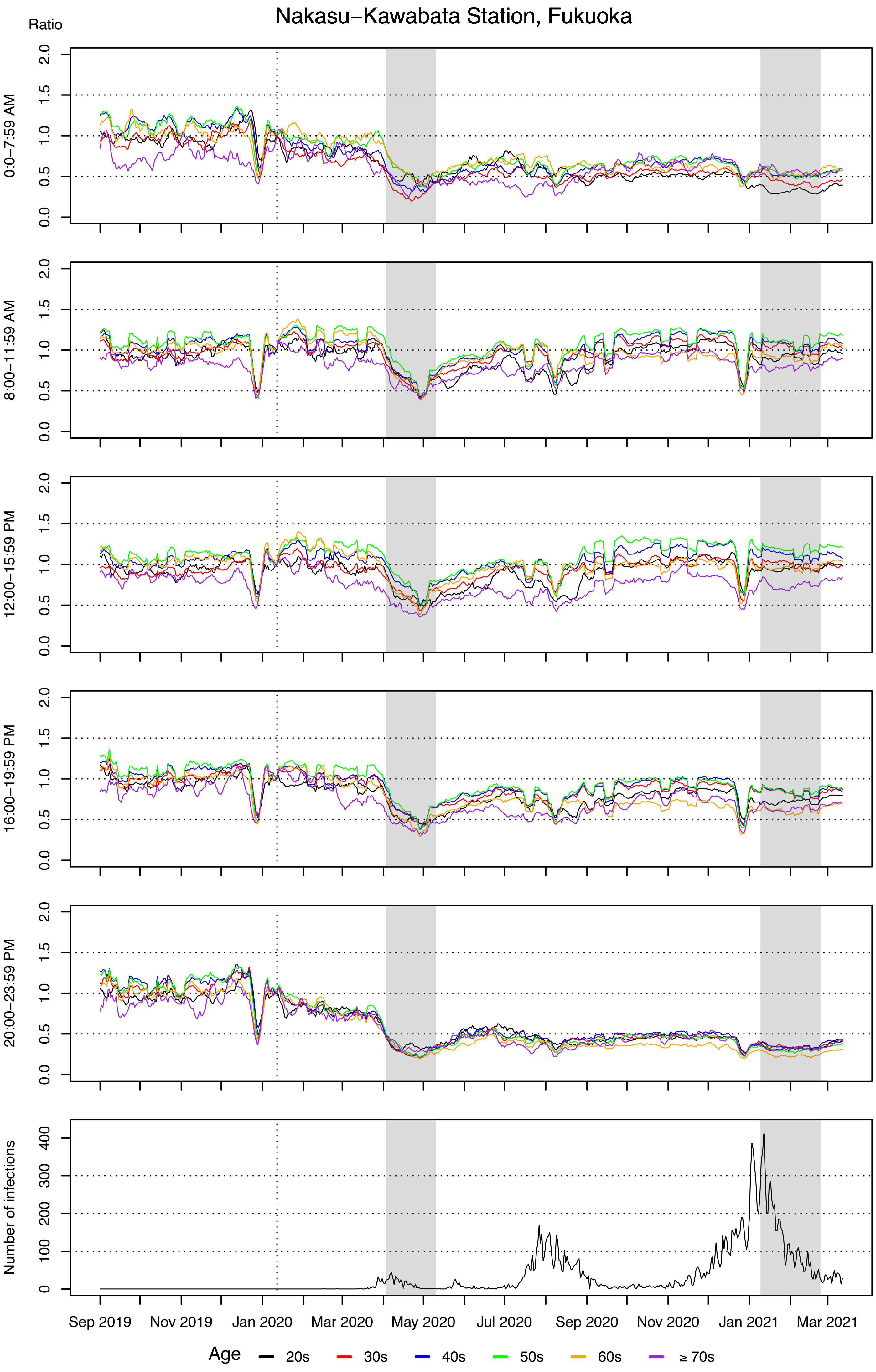

Nakasu−Kawabata Station, Fukuoka
Ratio
0:0−7:59 AM
8:00−11:59 AM
12:00−15:59 PM
16:00−19:59 PM
20:00−23:59 PM
Number of infections
0.0
0.5
1.0
1.5
2.0
0
100
200
300
400
Sep 2019
Nov 2019
Jan 2020
Mar 2020
May 2020
Jul 2020
Sep 2020
Nov 2020
Jan 2021
Mar 2021
Age
20s
30s
40s
50s
60s
≥ 70s

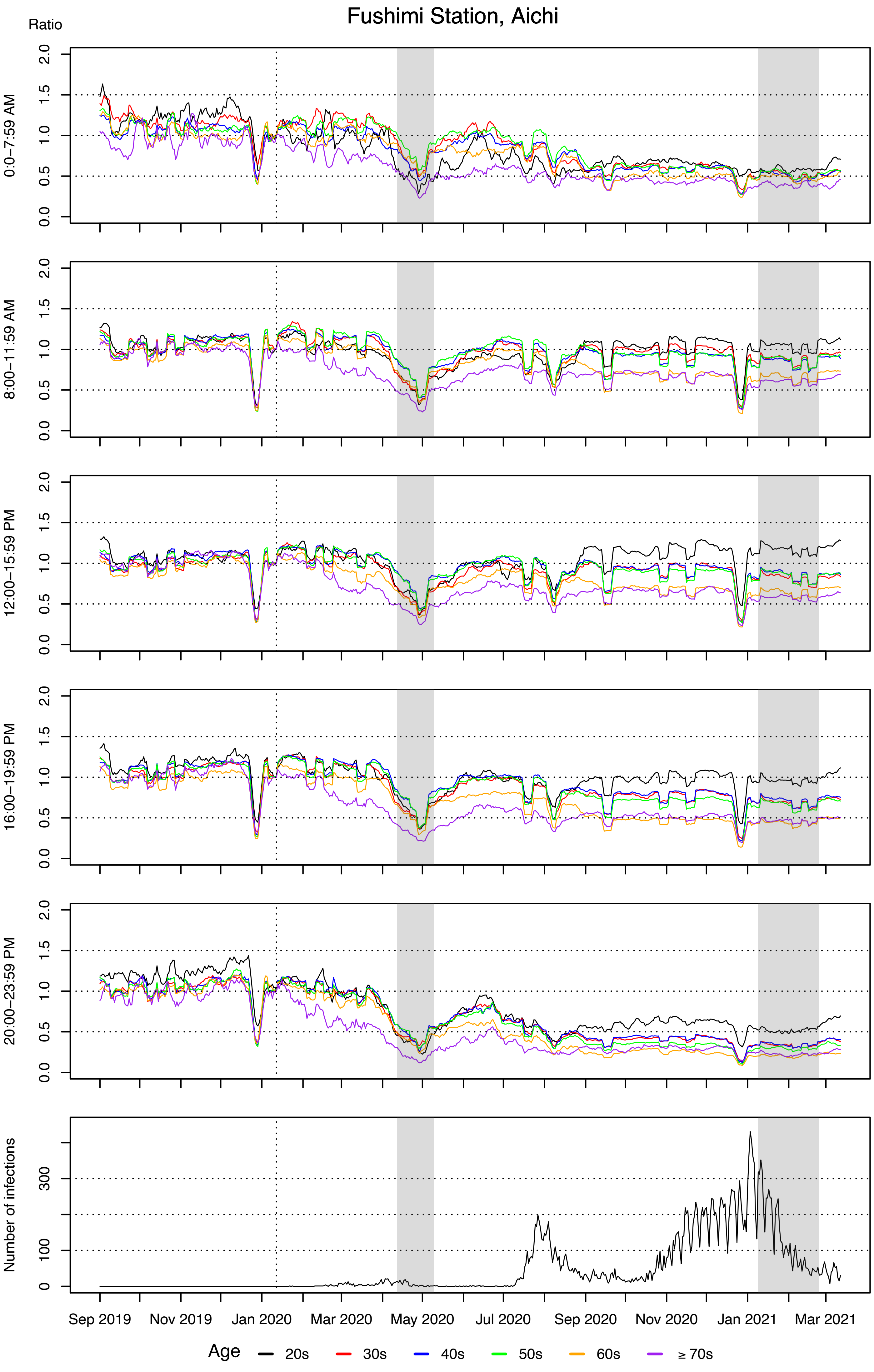
Fushimi Station, Aichi
Ratio
0:0−7:59 AM
8:00−11:59 AM
12:00−15:59 PM
16:00−19:59 PM
20:00−23:59 PM
Number of infections
Sep 2019
Nov 2019
Jan 2020
Mar 2020
May 2020
Jul 2020
Sep 2020
Nov 2020
Jan 2021
Mar 2021
Age
20s
30s
40s
50s
60s
≥ 70s

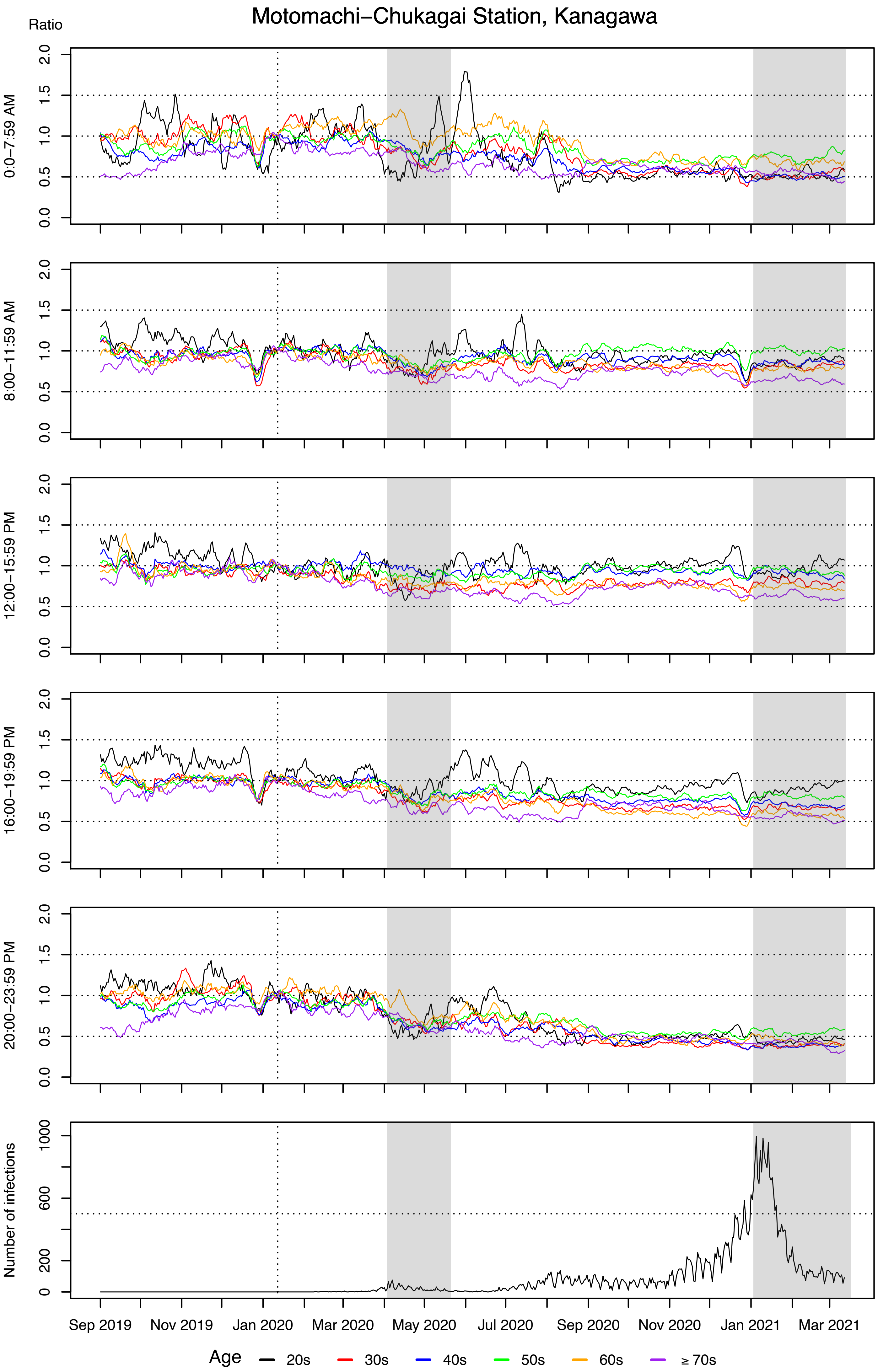
Motomachi−Chukagai Station, Kanagawa
Ratio
0:0−7:59 AM
8:00−11:59 AM
12:00−15:59 PM
16:00−19:59 PM
20:00−23:59 PM
0.0
0.5
1.0
1.5
2.0
Number of infections
0
200
600
1000
Sep 2019
Nov 2019
Jan 2020
Mar 2020
May 2020
Jul 2020
Sep 2020
Nov 2020
Jan 2021
Mar 2021
Age
20s
30s
40s
50s
60s
≥ 70s

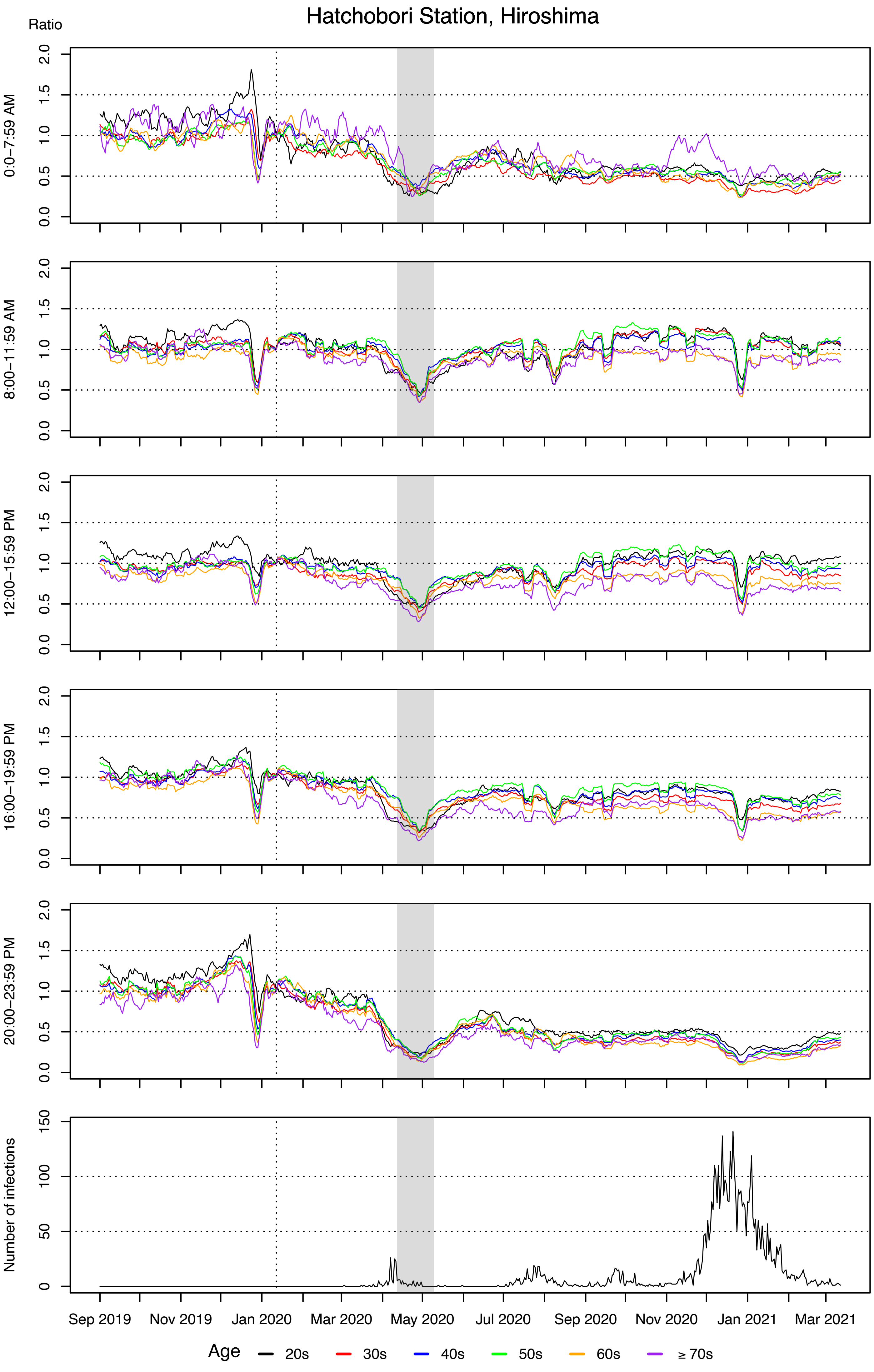

Hatchobori Station, Hiroshima
Ratio
0:0−7:59 AM
8:00−11:59 AM
12:00−15:59 PM
16:00−19:59 PM
20:00−23:59 PM
Number of infections
0.0
0.5
1.0
1.5
2.0
0
50
100
150
Sep 2019
Nov 2019
Jan 2020
Mar 2020
May 2020
Jul 2020
Sep 2020
Nov 2020
Jan 2021
Mar 2021
Age
20s
30s
40s
50s
60s
≥ 70s

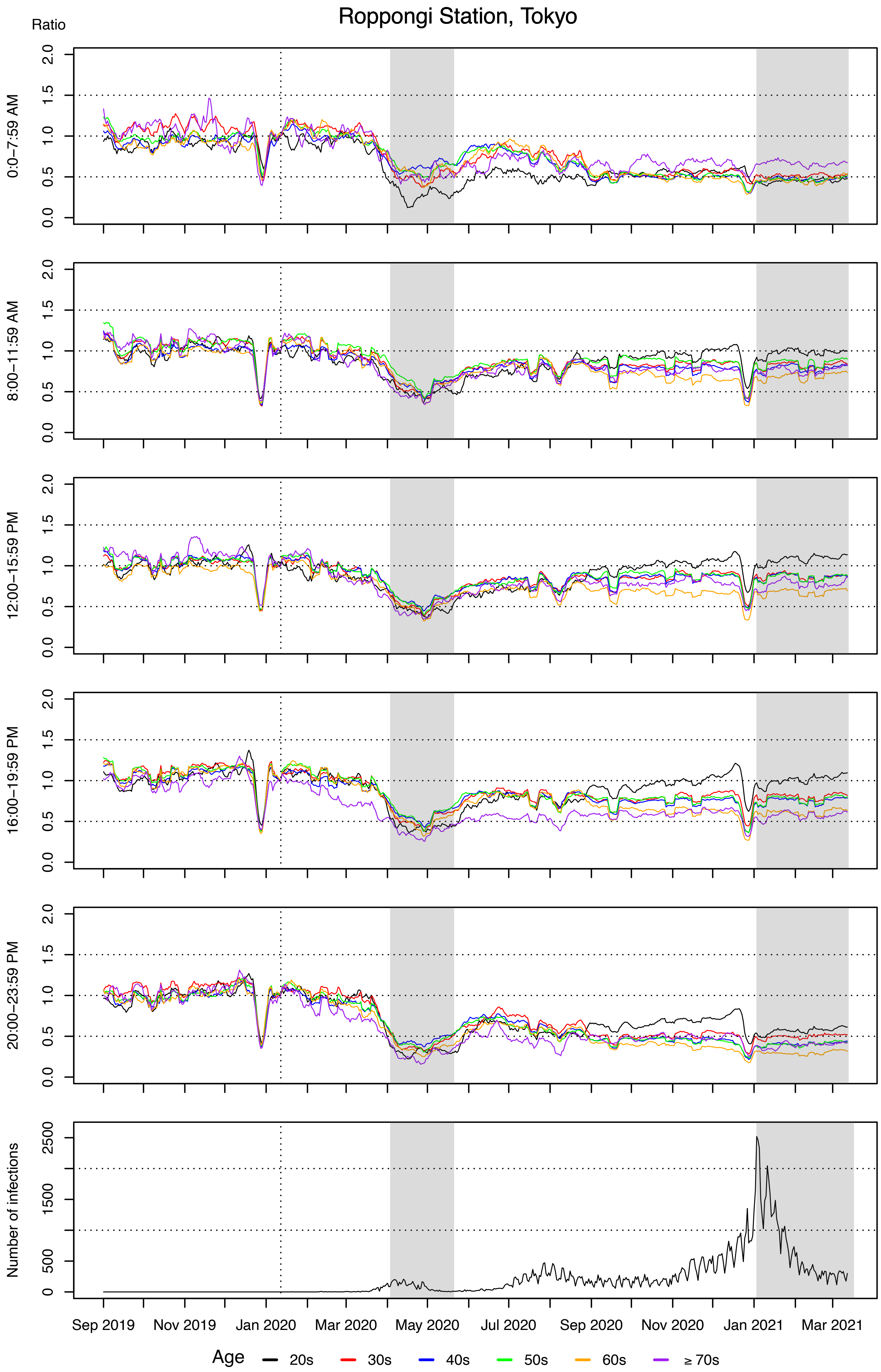
Roppongi Station, Tokyo
Ratio
0:0−7:59 AM
8:00−11:59 AM
12:00−15:59 PM
16:00−19:59 PM
20:00−23:59 PM
Number of infections
0.0
0.5
1.0
1.5
2.0
0
500
1500
2500
Sep 2019
Nov 2019
Jan 2020
Mar 2020
May 2020
Jul 2020
Sep 2020
Nov 2020
Jan 2021
Mar 2021
Age
20s
30s
40s
50s
60s
≥ 70s

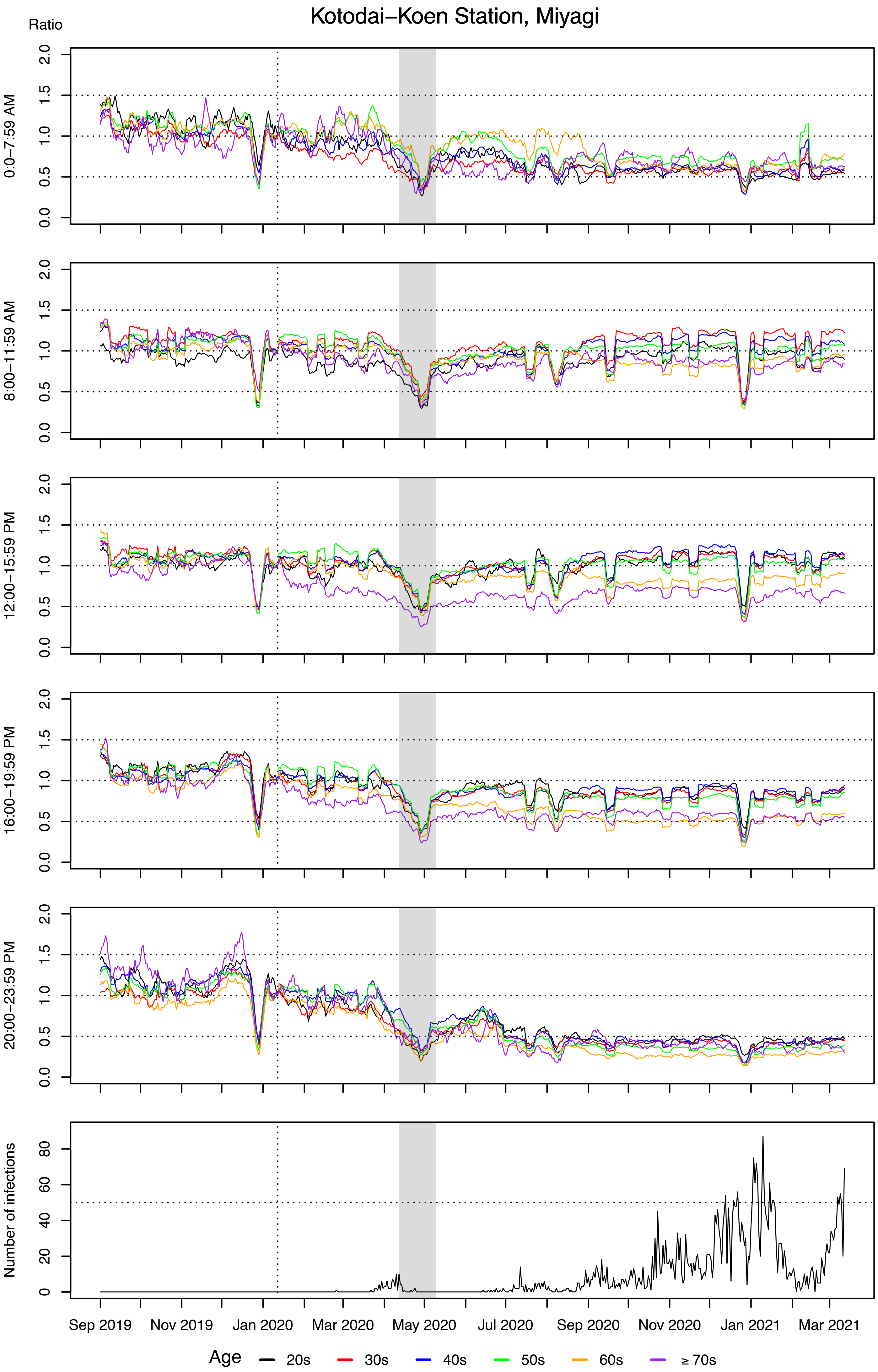

Kotodai–Koen Station, Miyagi
Ratio
0:0–7:59 AM
8:00–11:59 AM
12:00–15:59 PM
16:00–19:59 PM
20:00–23:59 PM
Number of infections
Sep 2019
Nov 2019
Jan 2020
Mar 2020
May 2020
Jul 2020
Sep 2020
Nov 2020
Jan 2021
Mar 2021
Age
20s
30s
40s
50s
60s
≥ 70s

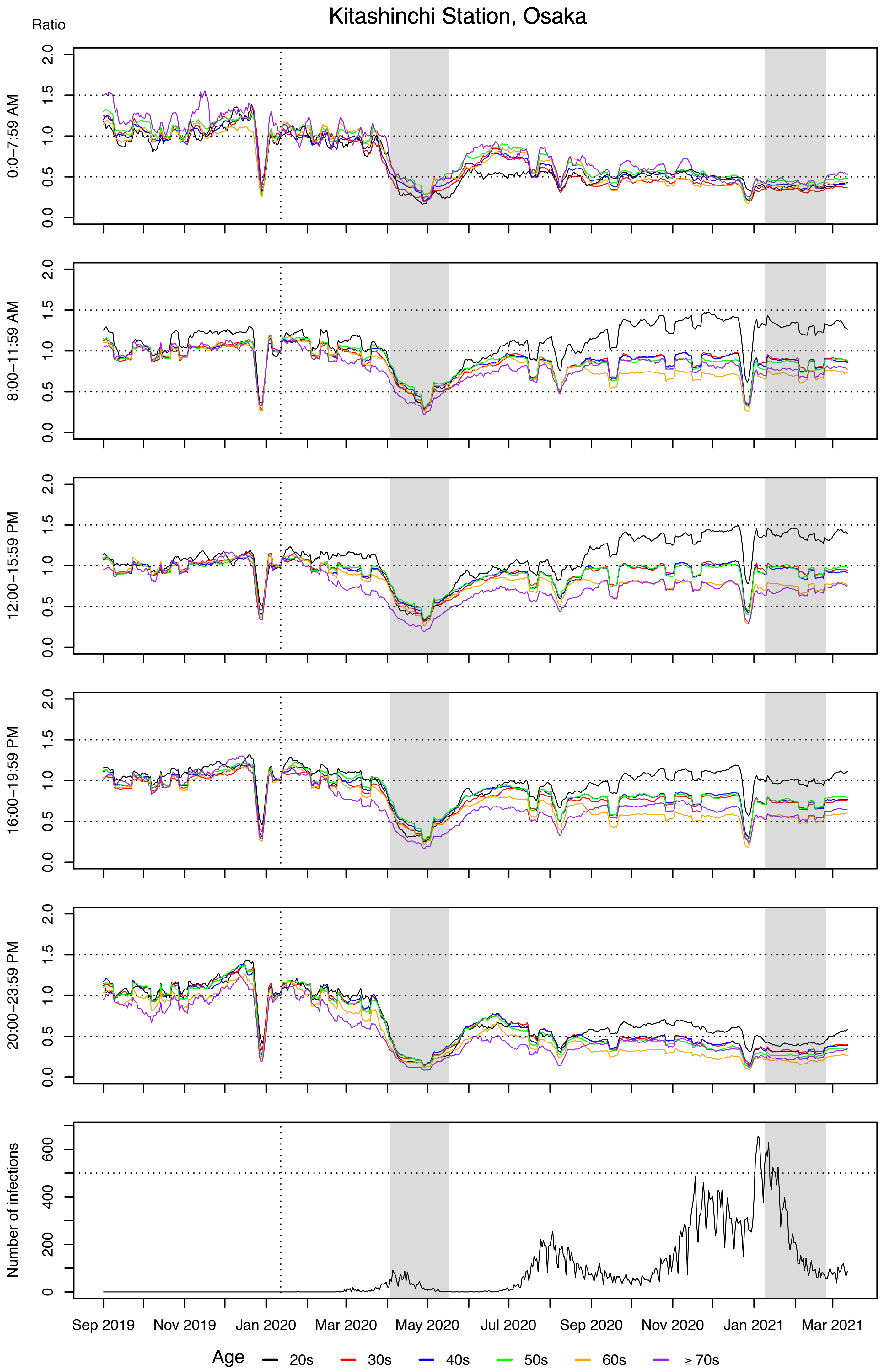
Kitashinchi Station, Osaka
Ratio
0:0−7:59 AM
8:00−11:59 AM
12:00−15:59 PM
16:00−19:59 PM
20:00−23:59 PM
Number of infections
0.0
0.5
1.0
1.5
2.0
0
200
400
600
Sep 2019
Nov 2019
Jan 2020
Mar 2020
May 2020
Jul 2020
Sep 2020
Nov 2020
Jan 2021
Mar 2021
Age
20s
30s
40s
50s
60s
≥ 70s

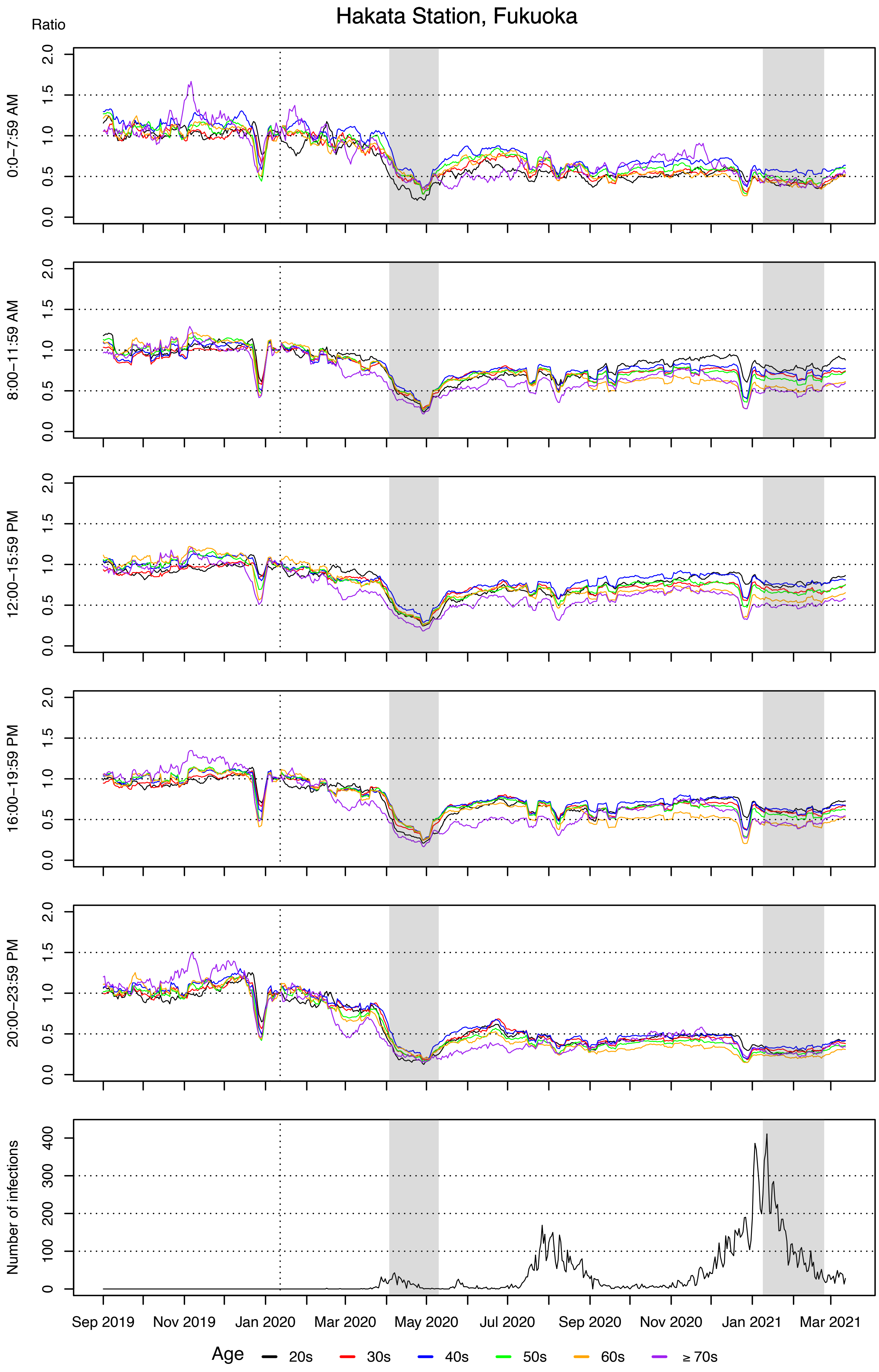

Hakata Station, Fukuoka
Ratio
0:0−7:59 AM
8:00−11:59 AM
12:00−15:59 PM
16:00−19:59 PM
20:00−23:59 PM
Number of infections
0.0
0.5
1.0
1.5
2.0
0
100
200
300
400
Sep 2019
Nov 2019
Jan 2020
Mar 2020
May 2020
Jul 2020
Sep 2020
Nov 2020
Jan 2021
Mar 2021
Age
20s
30s
40s
50s
60s
≥ 70s

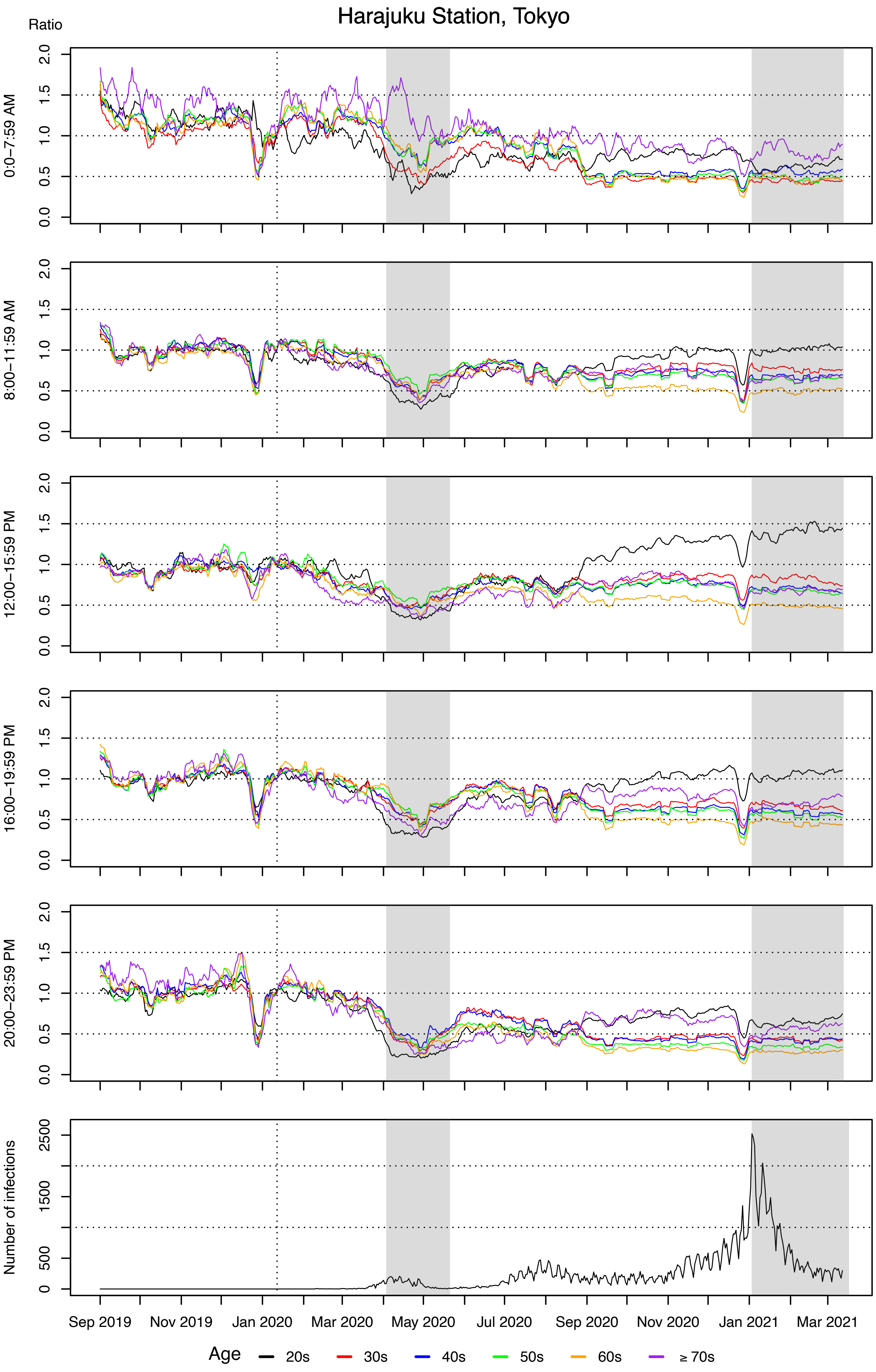

Harajuku Station, Tokyo
Ratio
0:0−7:59 AM
8:00−11:59 AM
12:00−15:59 PM
16:00−19:59 PM
20:00−23:59 PM
Number of infections
0.0
0.5
1.0
1.5
2.0
0
500
1500
2500
Sep 2019
Nov 2019
Jan 2020
Mar 2020
May 2020
Jul 2020
Sep 2020
Nov 2020
Jan 2021
Mar 2021
Age
20s
30s
40s
50s
60s
≥ 70s

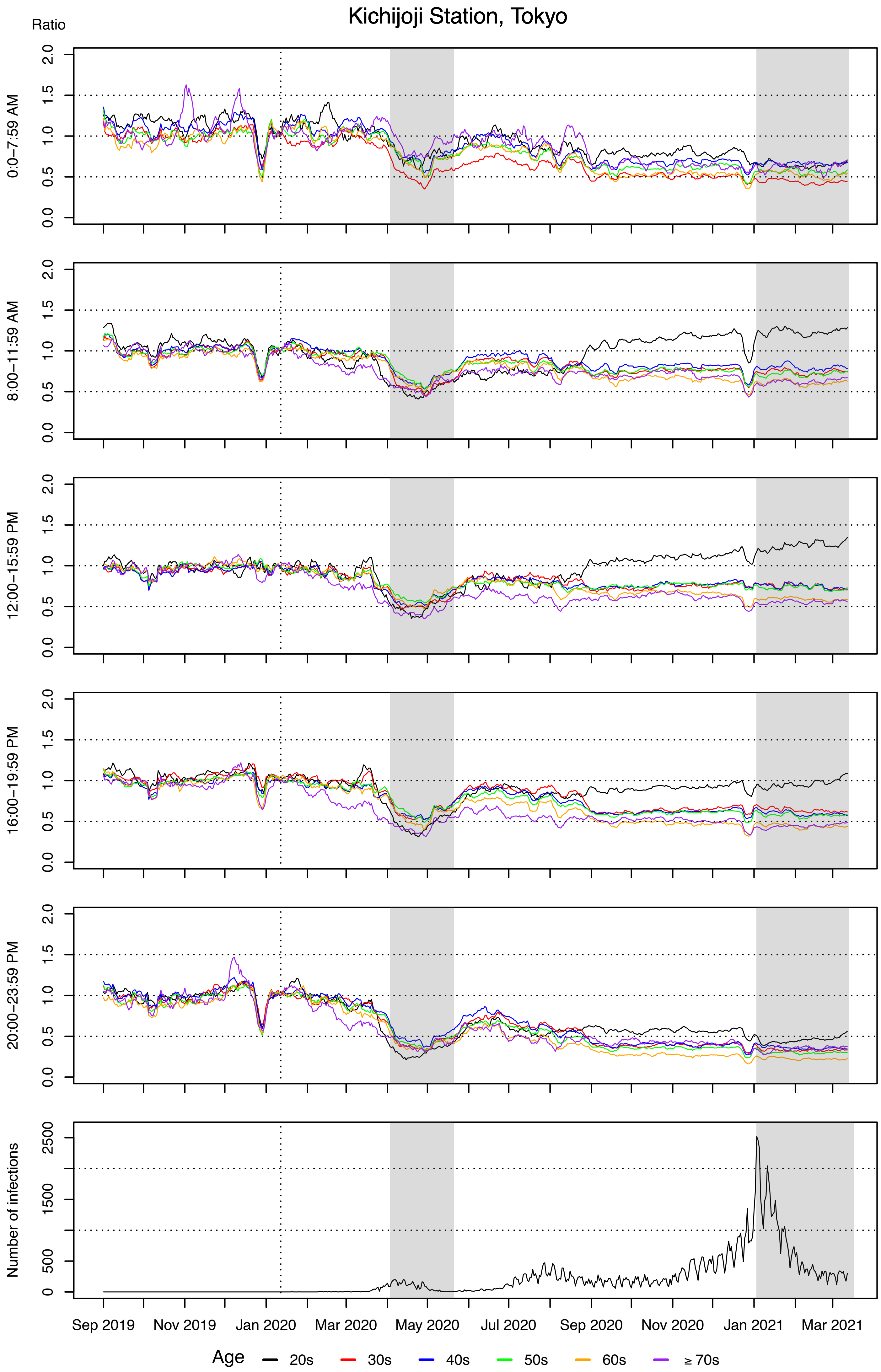

Kichijoji Station, Tokyo
Ratio
0:0−7:59 AM
8:00−11:59 AM
12:00−15:59 PM
16:00−19:59 PM
20:00−23:59 PM
Number of infections
0.0
0.5
1.0
1.5
2.0
0
500
1500
2500
Sep 2019
Nov 2019
Jan 2020
Mar 2020
May 2020
Jul 2020
Sep 2020
Nov 2020
Jan 2021
Mar 2021
Age
20s
30s
40s
50s
60s
≥ 70s

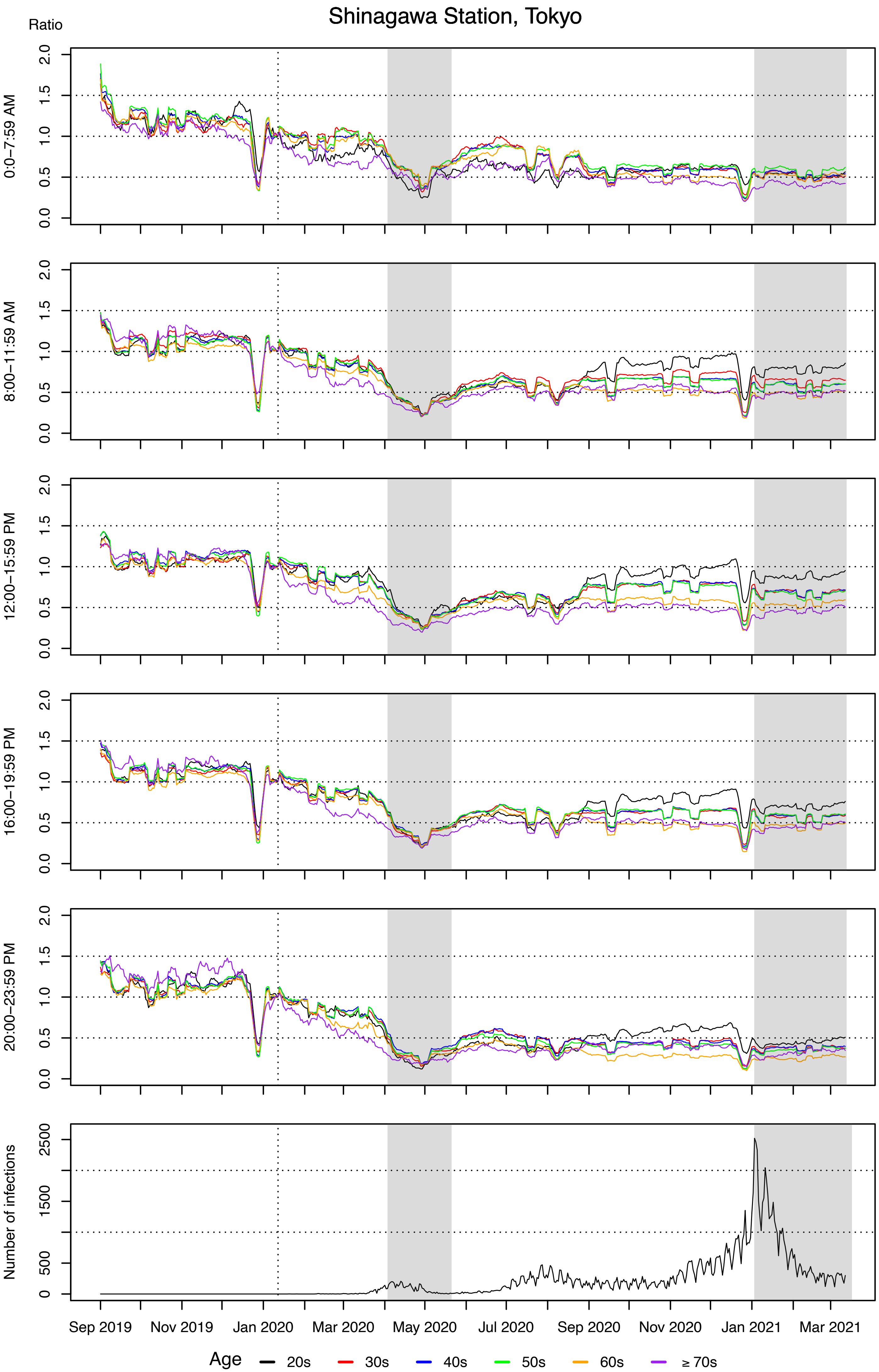
Shinagawa Station, Tokyo
Ratio
0:0−7:59 AM
8:00−11:59 AM
12:00−15:59 PM
16:00−19:59 PM
20:00−23:59 PM
Number of infections
0.0
0.5
1.0
1.5
2.0
0
500
1500
2500
Sep 2019
Nov 2019
Jan 2020
Mar 2020
May 2020
Jul 2020
Sep 2020
Nov 2020
Jan 2021
Mar 2021
Age
20s
30s
40s
50s
60s
≥ 70s

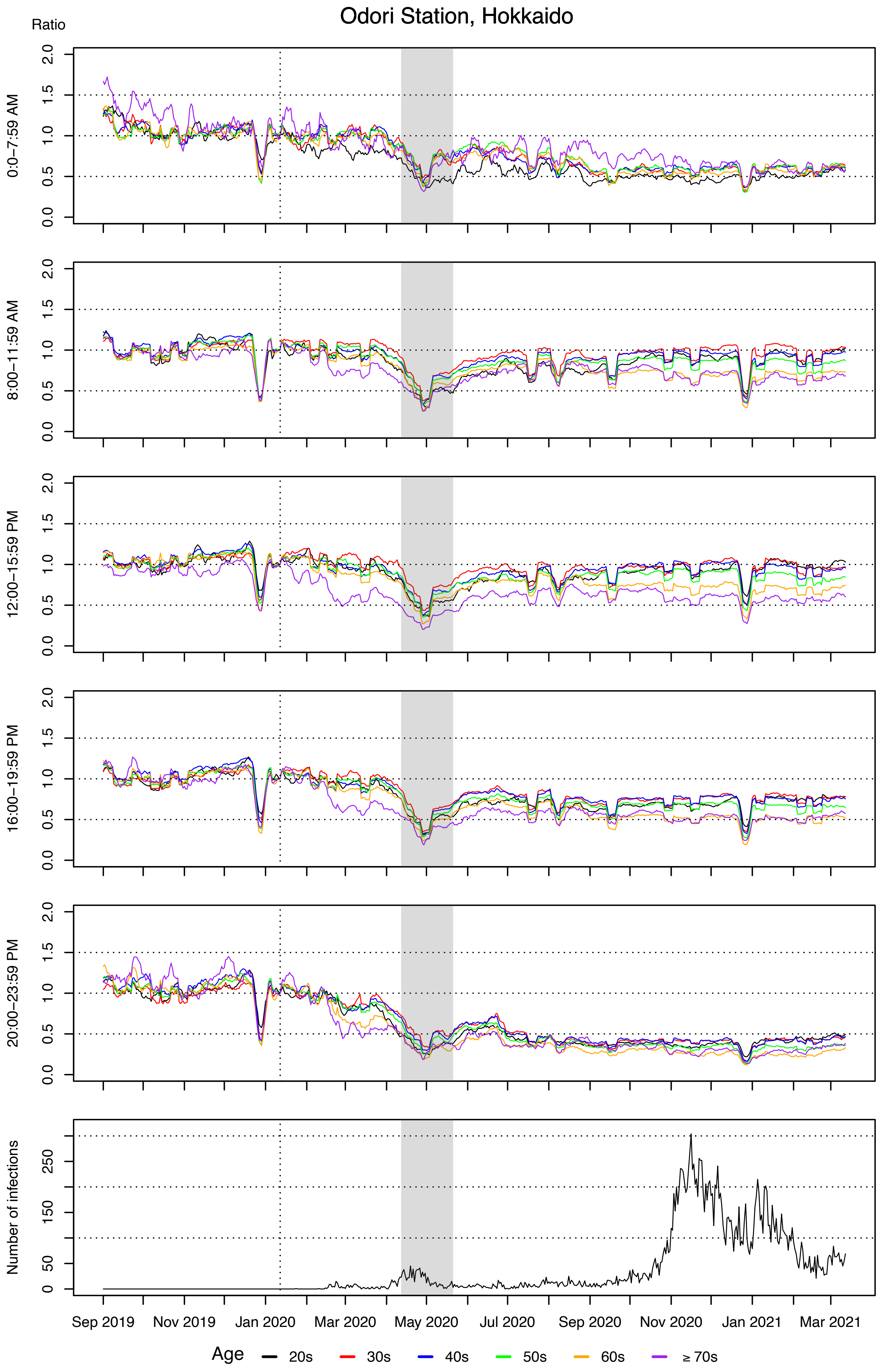

Odori Station, Hokkaido
Ratio
0:0−7:59 AM
8:00−11:59 AM
12:00−15:59 PM
16:00−19:59 PM
20:00−23:59 PM
Number of infections
Sep 2019
Nov 2019
Jan 2020
Mar 2020
May 2020
Jul 2020
Sep 2020
Nov 2020
Jan 2021
Mar 2021
Age
20s
30s
40s
50s
60s
≥ 70s

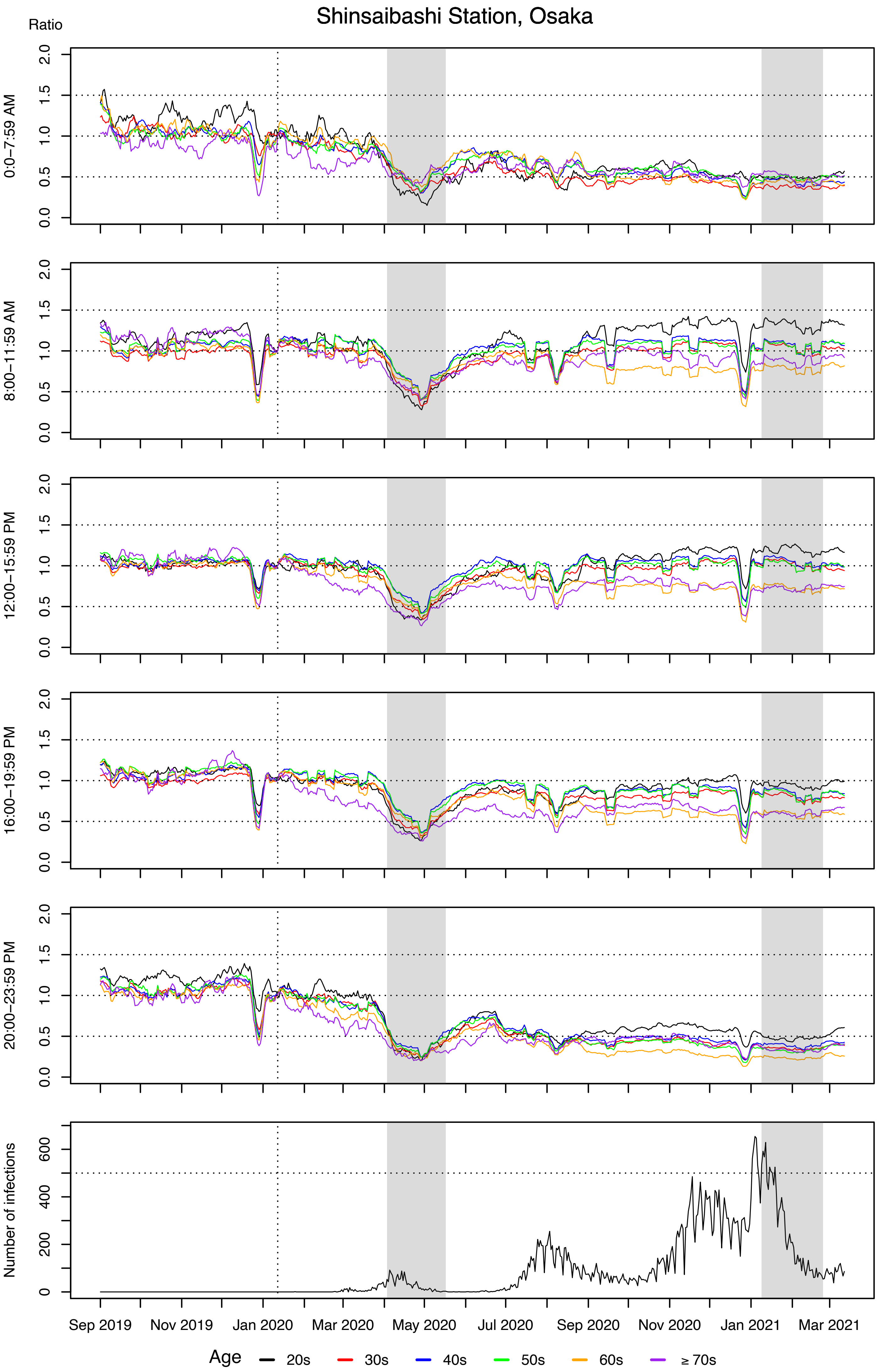
Shinsaibashi Station, Osaka
Ratio
0:0−7:59 AM
8:00−11:59 AM
12:00−15:59 PM
16:00−19:59 PM
20:00−23:59 PM
Number of infections
0.0
0.5
1.0
1.5
2.0
0
200
400
600
Sep 2019
Nov 2019
Jan 2020
Mar 2020
May 2020
Jul 2020
Sep 2020
Nov 2020
Jan 2021
Mar 2021
Age
20s
30s
40s
50s
60s
≥ 70s

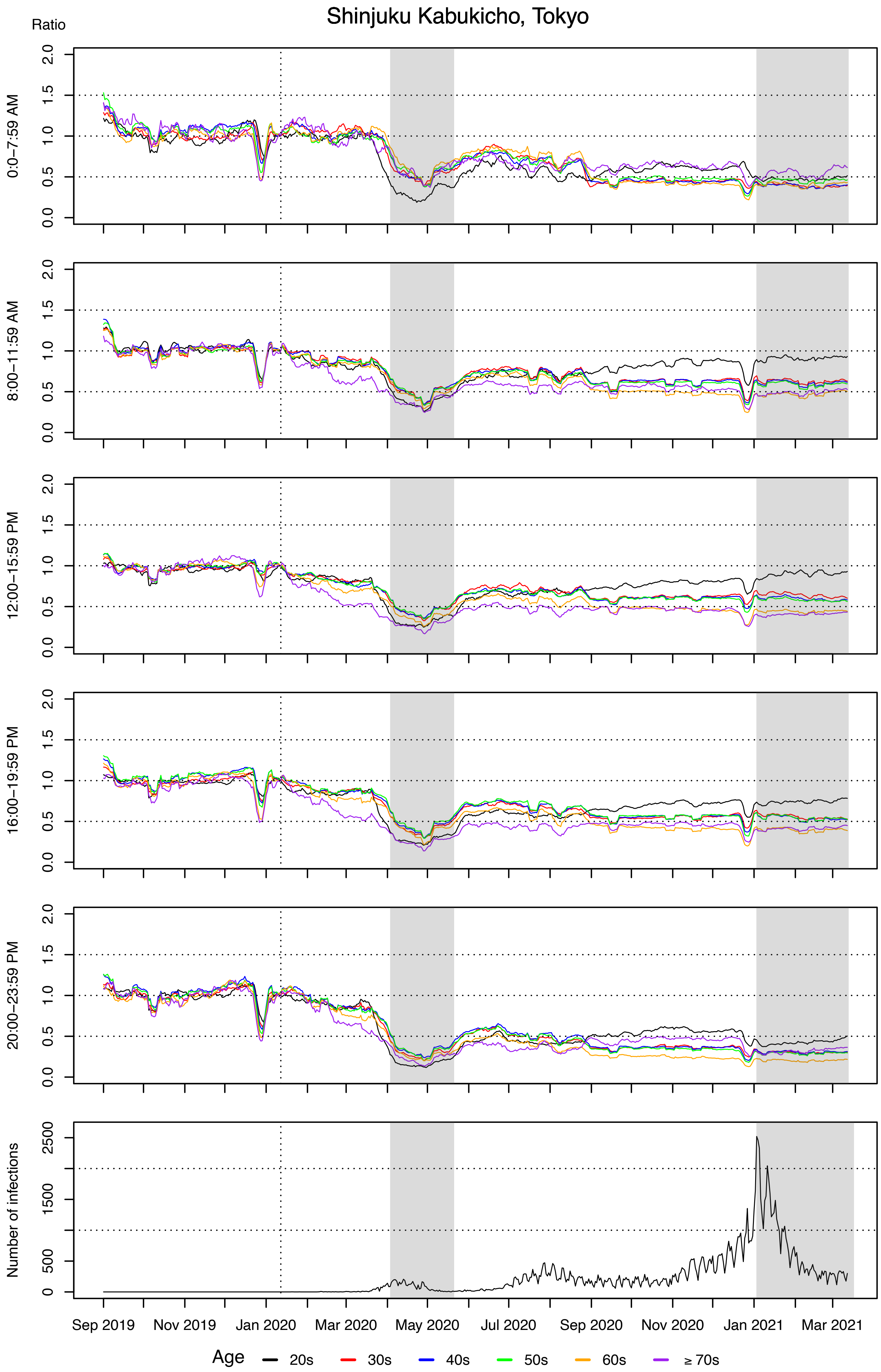

Shinjuku Kabukicho, Tokyo
Ratio
0:0−7:59 AM
8:00−11:59 AM
12:00−15:59 PM
16:00−19:59 PM
20:00−23:59 PM
Number of infections
0.0
0.5
1.0
1.5
2.0
0
500
1500
2500
Sep 2019
Nov 2019
Jan 2020
Mar 2020
May 2020
Jul 2020
Sep 2020
Nov 2020
Jan 2021
Mar 2021
Age
20s
30s
40s
50s
60s
≥ 70s

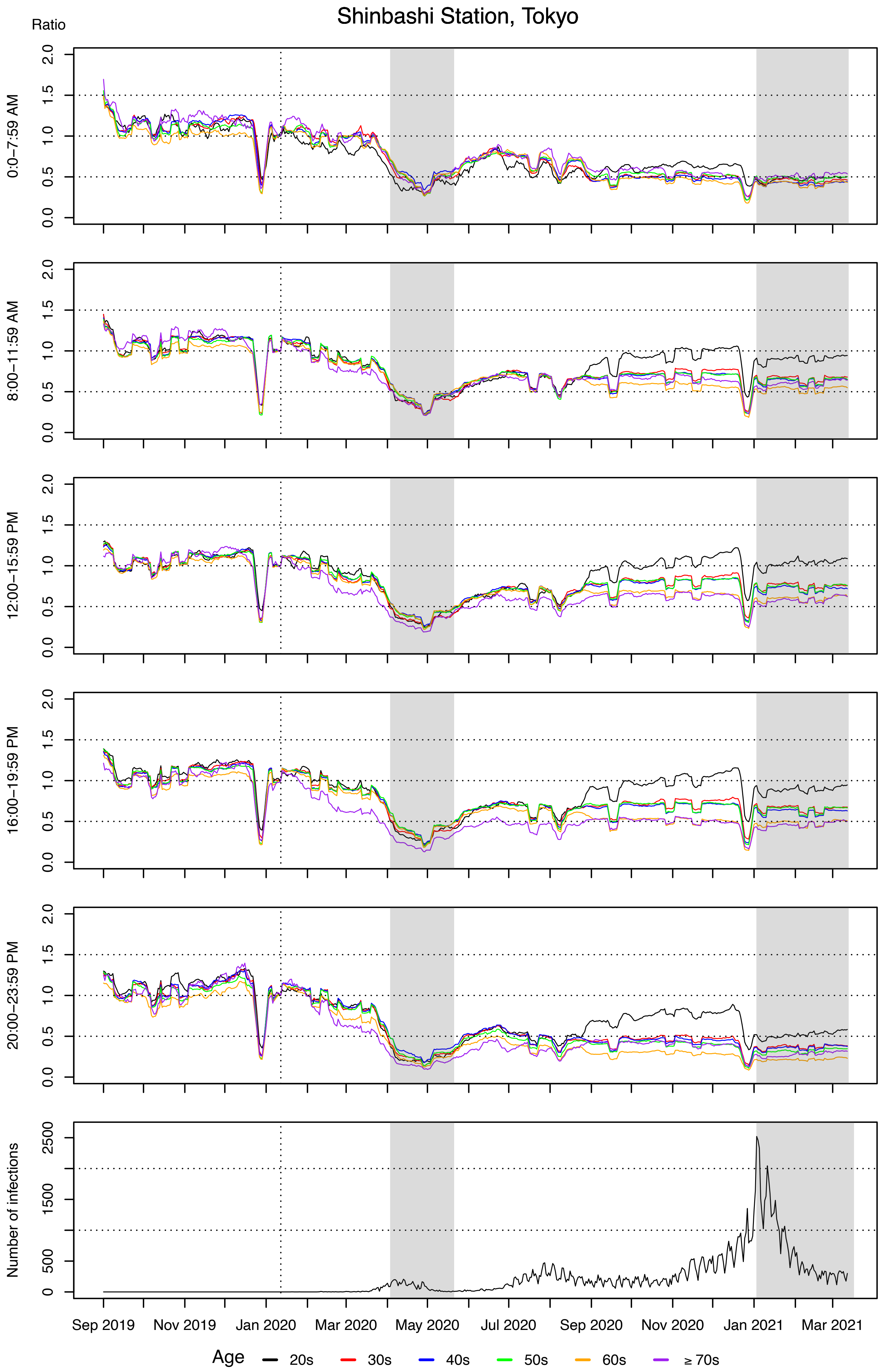

Shinbashi Station, Tokyo
Ratio
0:0−7:59 AM
8:00−11:59 AM
12:00−15:59 PM
16:00−19:59 PM
20:00−23:59 PM
0.0
0.5
1.0
1.5
2.0
Number of infections
0
500
1500
2500
Sep 2019
Nov 2019
Jan 2020
Mar 2020
May 2020
Jul 2020
Sep 2020
Nov 2020
Jan 2021
Mar 2021
Age
20s
30s
40s
50s
60s
≥ 70s

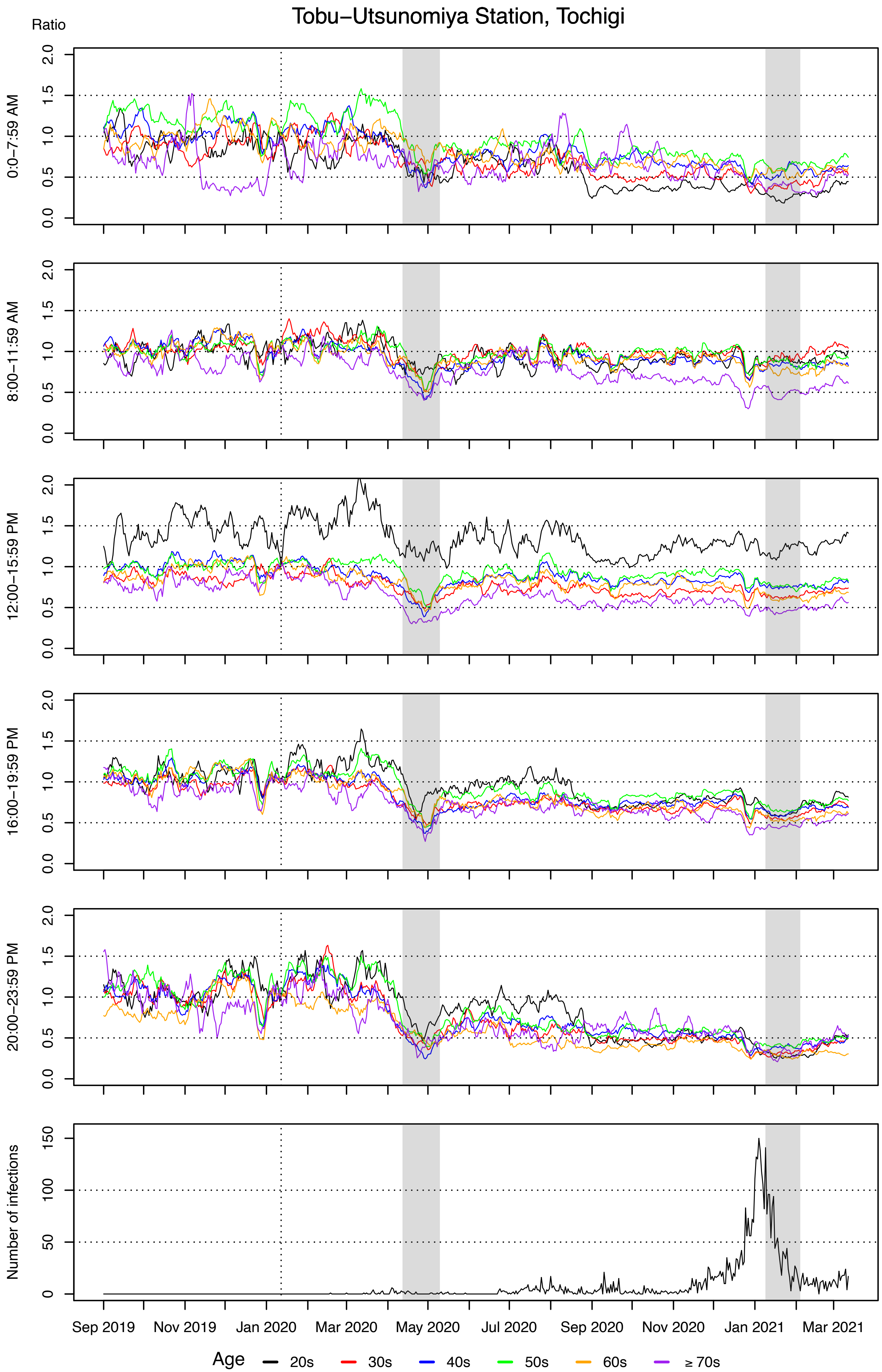
Tobu−Utsunomiya Station, Tochigi
Ratio
0:0−7:59 AM
8:00−11:59 AM
12:00−15:59 PM
16:00−19:59 PM
20:00−23:59 PM
Number of infections
0.0
0.5
1.0
1.5
2.0
0
50
100
150
Sep 2019
Nov 2019
Jan 2020
Mar 2020
May 2020
Jul 2020
Sep 2020
Nov 2020
Jan 2021
Mar 2021
Age
20s
30s
40s
50s
60s
≥ 70s

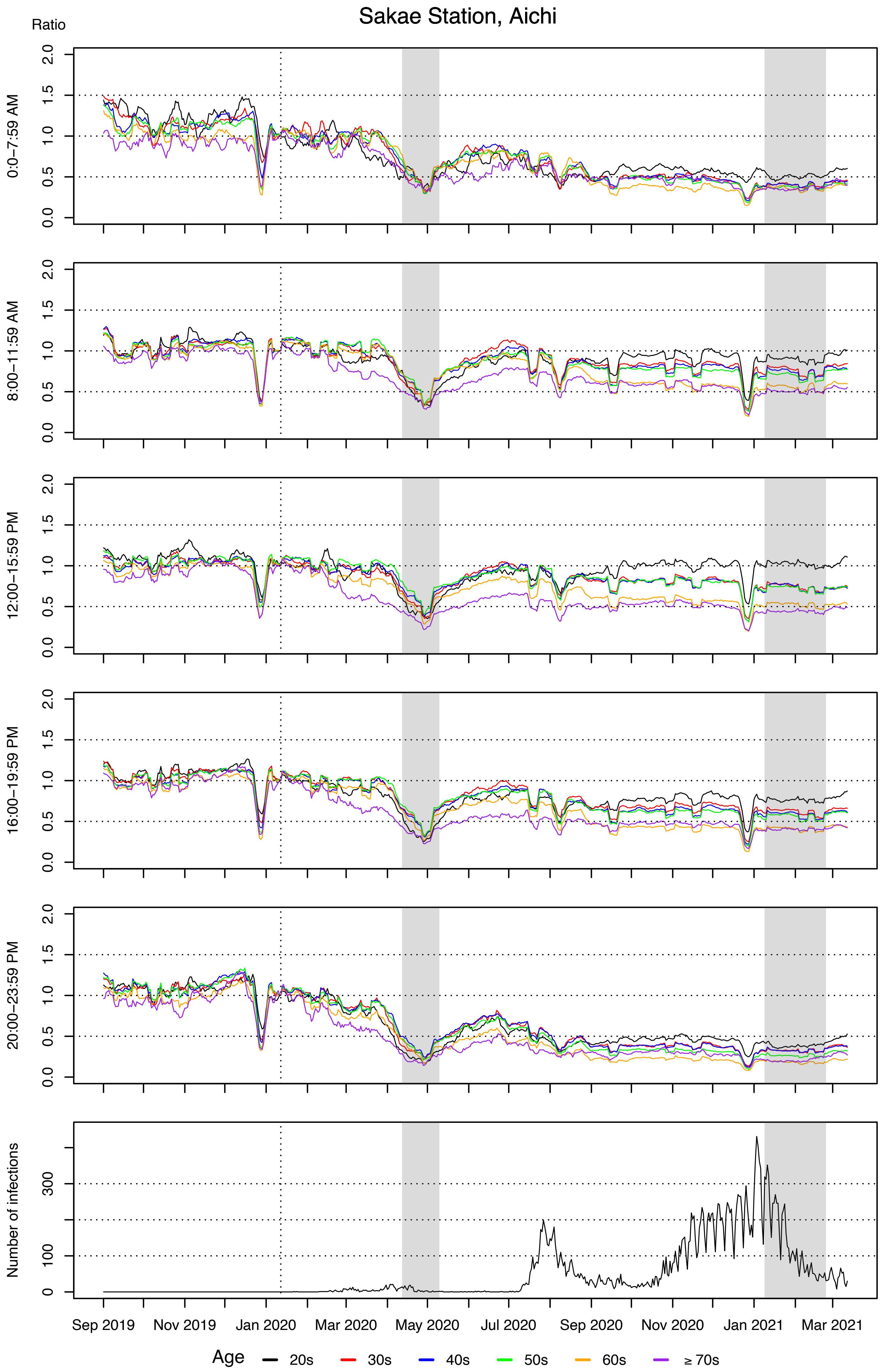
Sakae Station, Aichi
Ratio
0:0−7:59 AM
8:00−11:59 AM
12:00−15:59 PM
16:00−19:59 PM
20:00−23:59 PM
Number of infections
Sep 2019
Nov 2019
Jan 2020
Mar 2020
May 2020
Jul 2020
Sep 2020
Nov 2020
Jan 2021
Mar 2021
Age
20s
30s
40s
50s
60s
≥ 70s

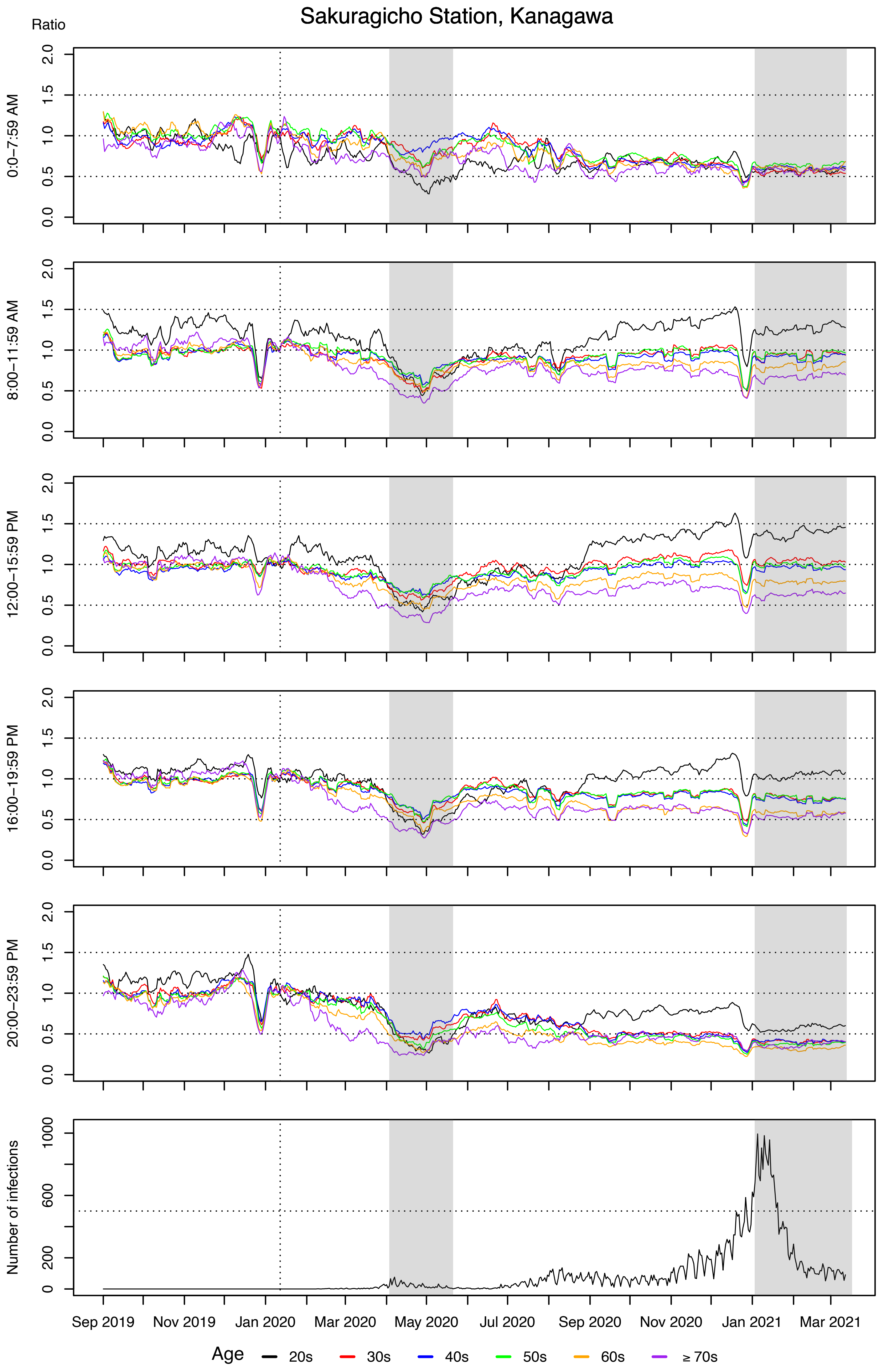
Sakuragicho Station, Kanagawa
Ratio
0:0−7:59 AM
8:00−11:59 AM
12:00−15:59 PM
16:00−19:59 PM
20:00−23:59 PM
Number of infections
0.0
0.5
1.0
1.5
2.0
0
200
600
1000
Sep 2019
Nov 2019
Jan 2020
Mar 2020
May 2020
Jul 2020
Sep 2020
Nov 2020
Jan 2021
Mar 2021
Age
20s
30s
40s
50s
60s
≥ 70s

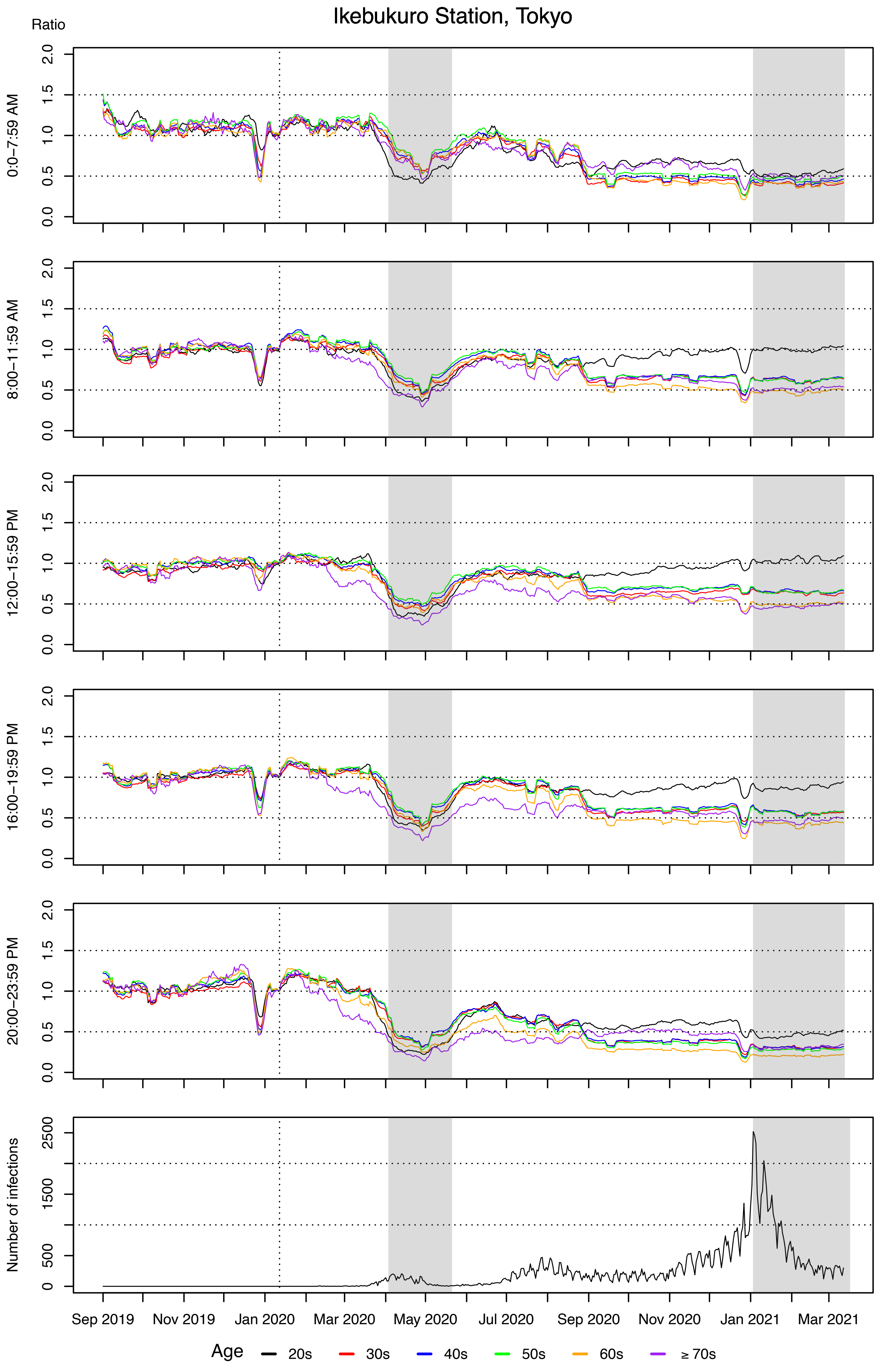

Ikebukuro Station, Tokyo
Ratio
0:0−7:59 AM
8:00−11:59 AM
12:00−15:59 PM
16:00−19:59 PM
20:00−23:59 PM
Number of infections
0.0
0.5
1.0
1.5
2.0
0
500
1500
2500
Sep 2019
Nov 2019
Jan 2020
Mar 2020
May 2020
Jul 2020
Sep 2020
Nov 2020
Jan 2021
Mar 2021
Age
20s
30s
40s
50s
60s
≥ 70s

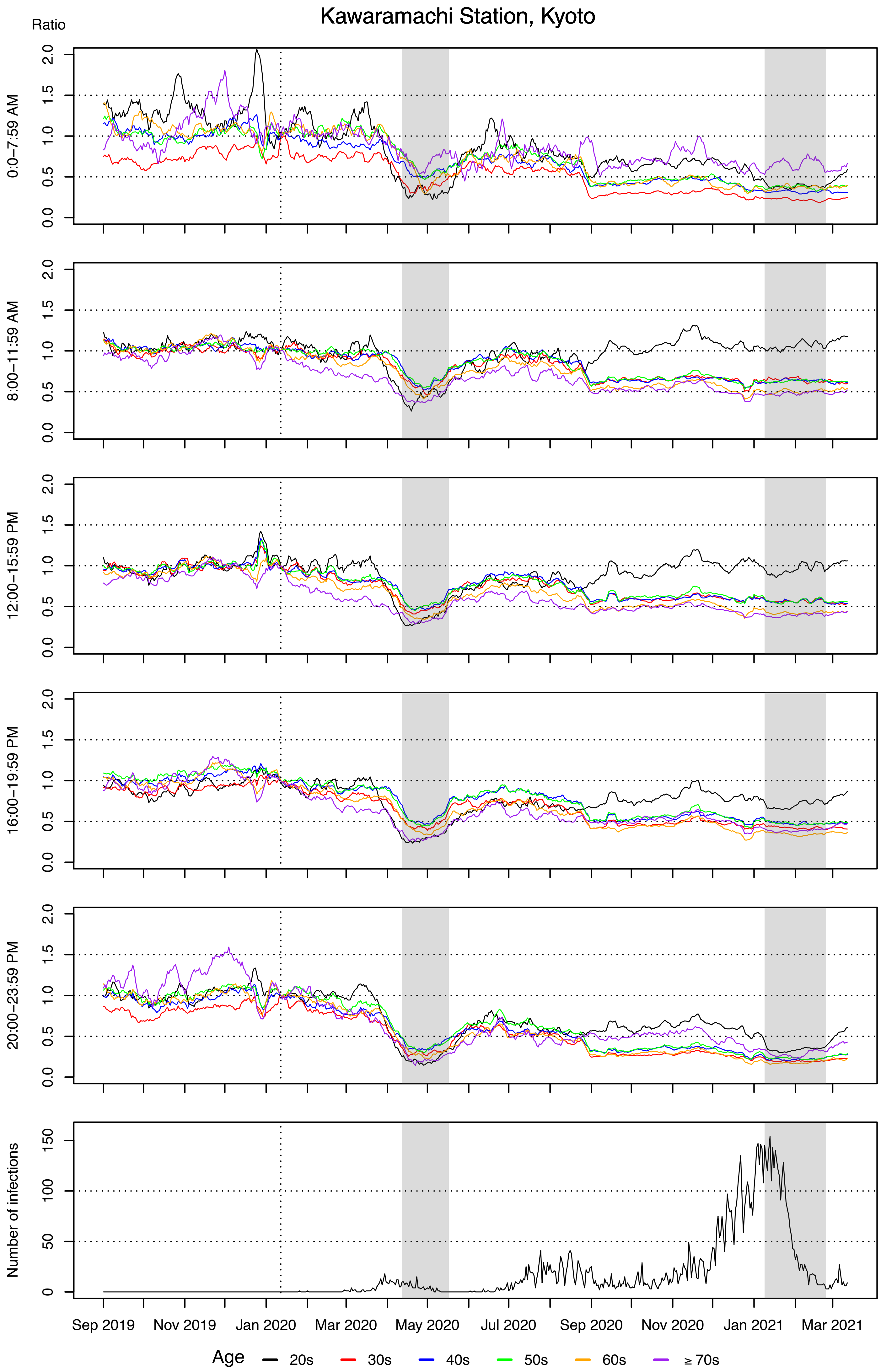
Kawaramachi Station, Kyoto
Ratio
0:0−7:59 AM
8:00−11:59 AM
12:00−15:59 PM
16:00−19:59 PM
20:00−23:59 PM
Number of infections
0.0
0.5
1.0
1.5
2.0
0
50
100
150
Sep 2019
Nov 2019
Jan 2020
Mar 2020
May 2020
Jul 2020
Sep 2020
Nov 2020
Jan 2021
Mar 2021
Age
20s
30s
40s
50s
60s
≥ 70s

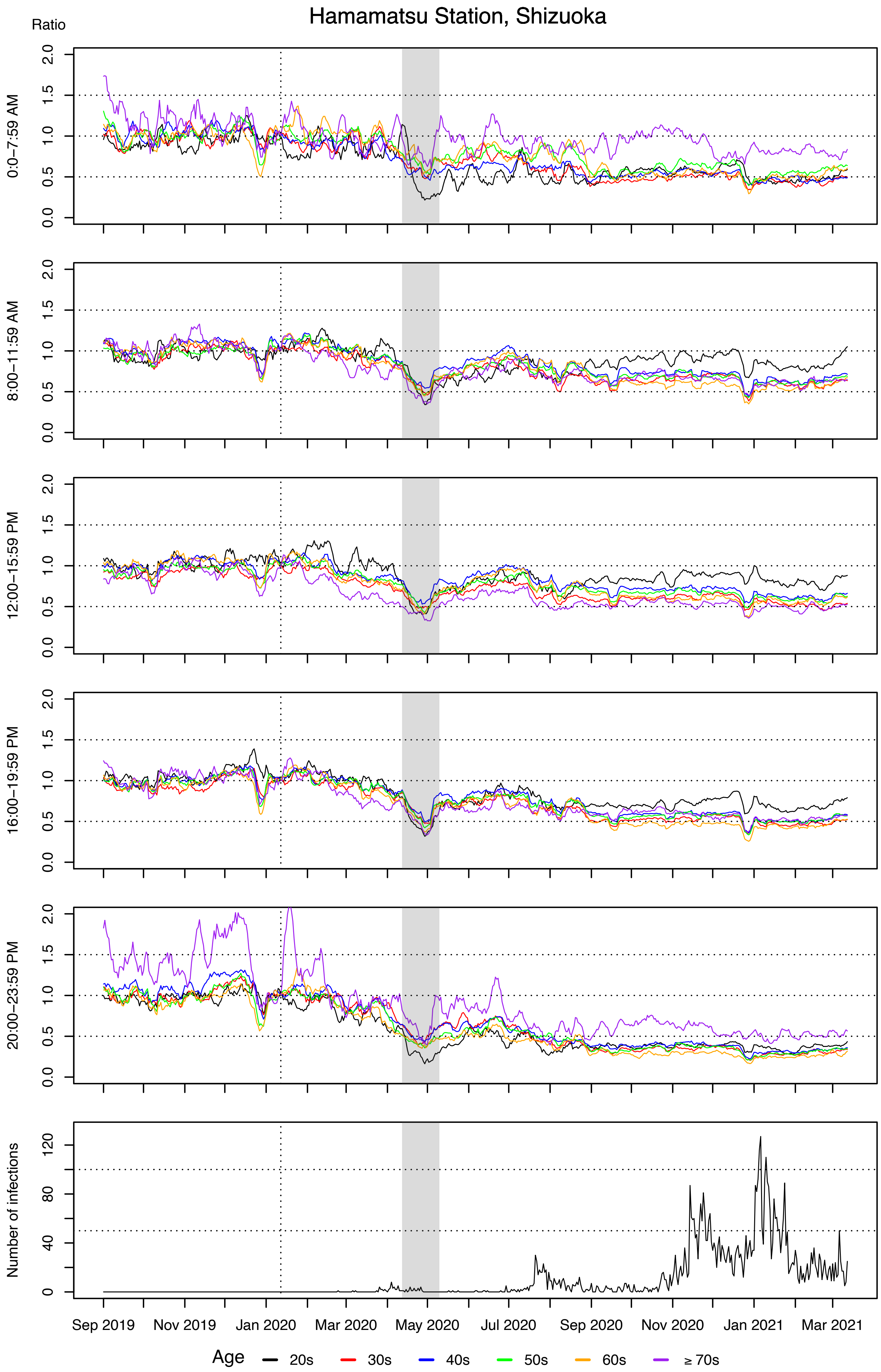

Hamamatsu Station, Shizuoka
Ratio
0:0−7:59 AM
8:00−11:59 AM
12:00−15:59 PM
16:00−19:59 PM
20:00−23:59 PM
Number of infections
0.0
0.5
1.0
1.5
2.0
0
40
80
120
Sep 2019
Nov 2019
Jan 2020
Mar 2020
May 2020
Jul 2020
Sep 2020
Nov 2020
Jan 2021
Mar 2021
Age
20s
30s
40s
50s
60s
≥ 70s

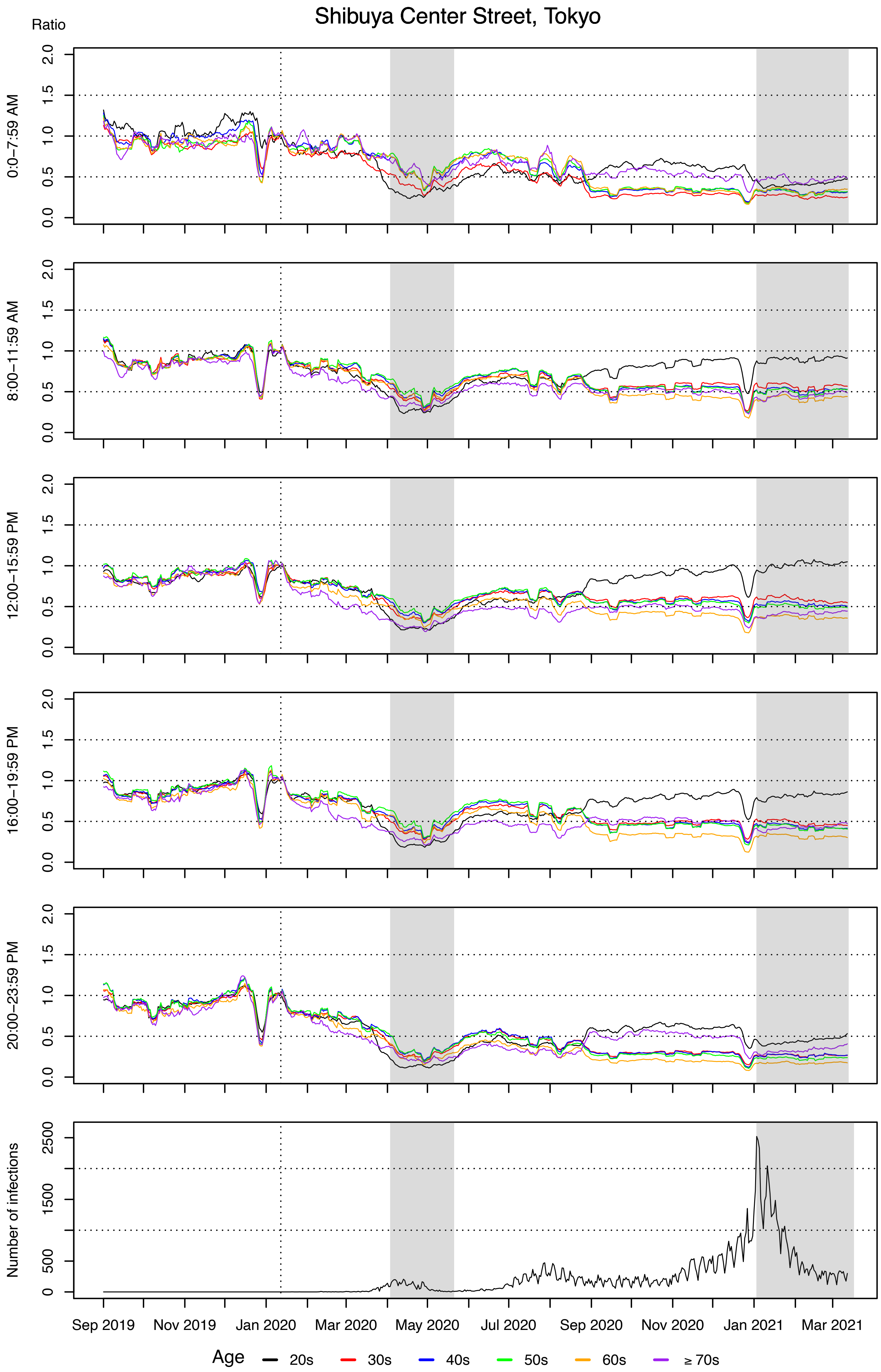

Shibuya Center Street, Tokyo
Ratio
0:0−7:59 AM
8:00−11:59 AM
12:00−15:59 PM
16:00−19:59 PM
20:00−23:59 PM
Number of infections
0.0
0.5
1.0
1.5
2.0
0
500
1500
2500
Sep 2019
Nov 2019
Jan 2020
Mar 2020
May 2020
Jul 2020
Sep 2020
Nov 2020
Jan 2021
Mar 2021
Age
20s
30s
40s
50s
60s
≥ 70s

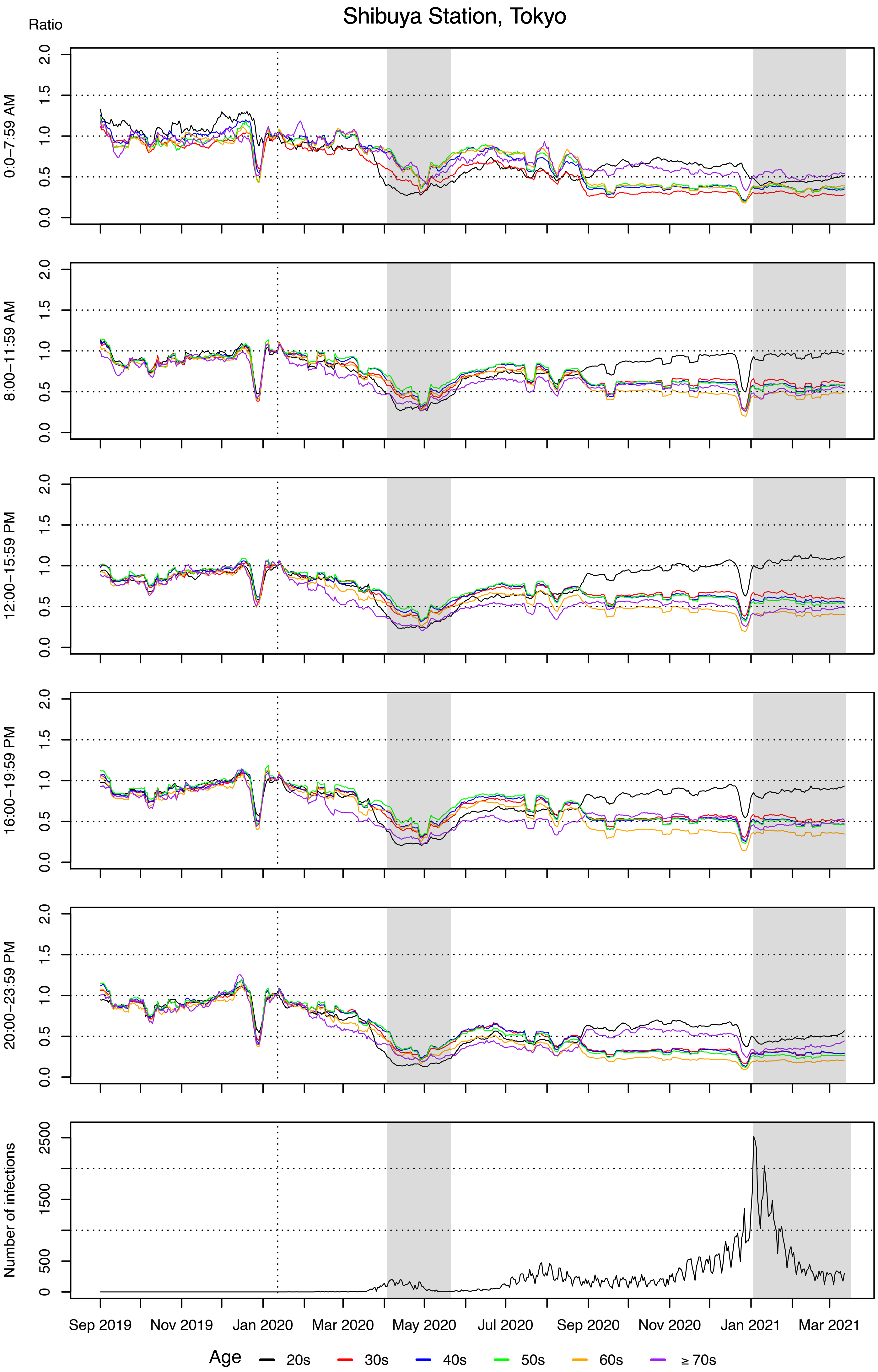
Shibuya Station, Tokyo
Ratio
0:0−7:59 AM
8:00−11:59 AM
12:00−15:59 PM
16:00−19:59 PM
20:00−23:59 PM
Number of infections
0.0
0.5
1.0
1.5
2.0
0
500
1500
2500
Sep 2019
Nov 2019
Jan 2020
Mar 2020
May 2020
Jul 2020
Sep 2020
Nov 2020
Jan 2021
Mar 2021
Age
20s
30s
40s
50s
60s
≥ 70s

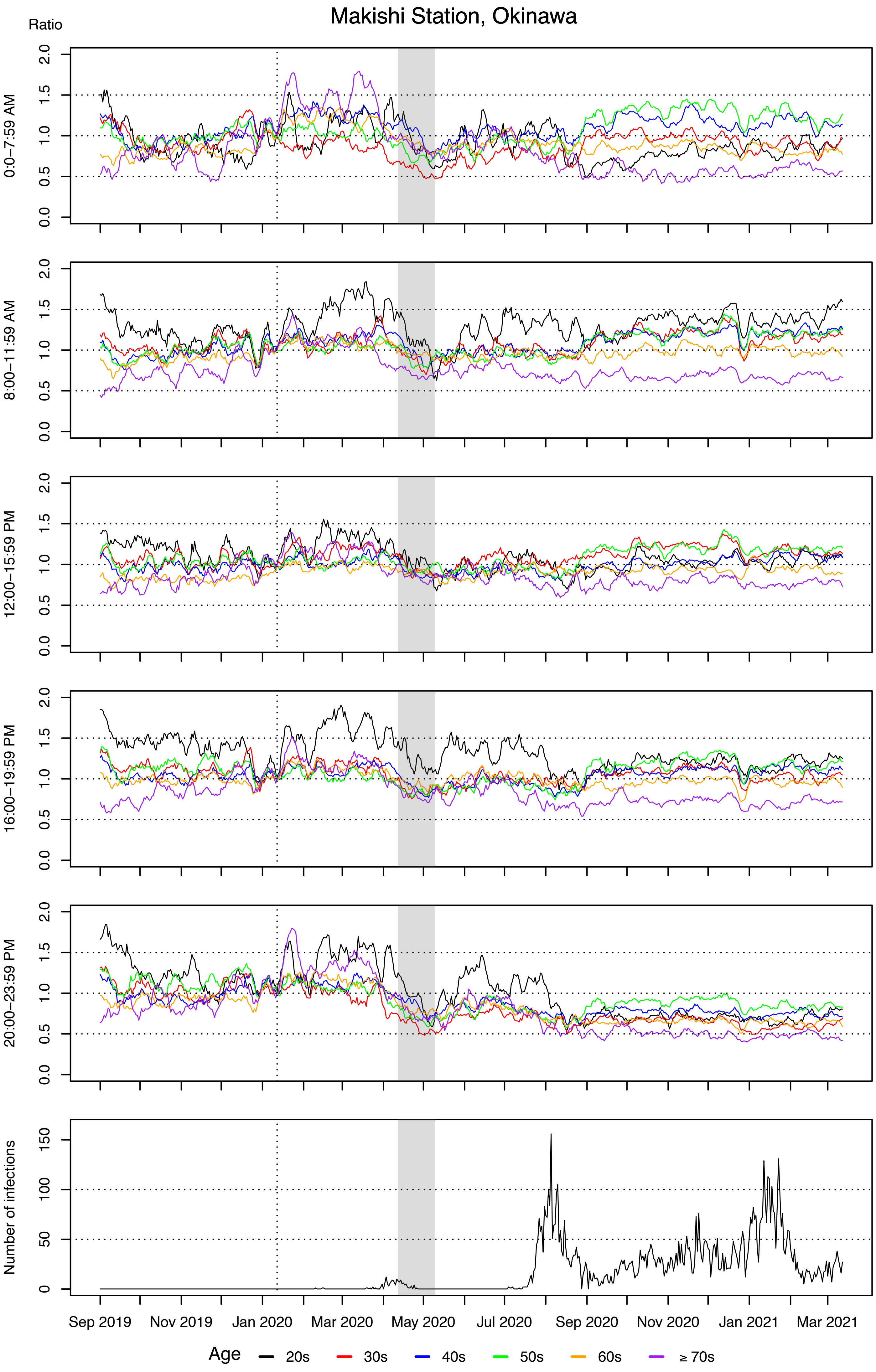

Makishi Station, Okinawa
Ratio
0:0−7:59 AM
8:00−11:59 AM
12:00−15:59 PM
16:00−19:59 PM
20:00−23:59 PM
Number of infections
0.0
0.5
1.0
1.5
2.0
0
50
100
150
Sep 2019
Nov 2019
Jan 2020
Mar 2020
May 2020
Jul 2020
Sep 2020
Nov 2020
Jan 2021
Mar 2021
Age
20s
30s
40s
50s
60s
≥ 70s

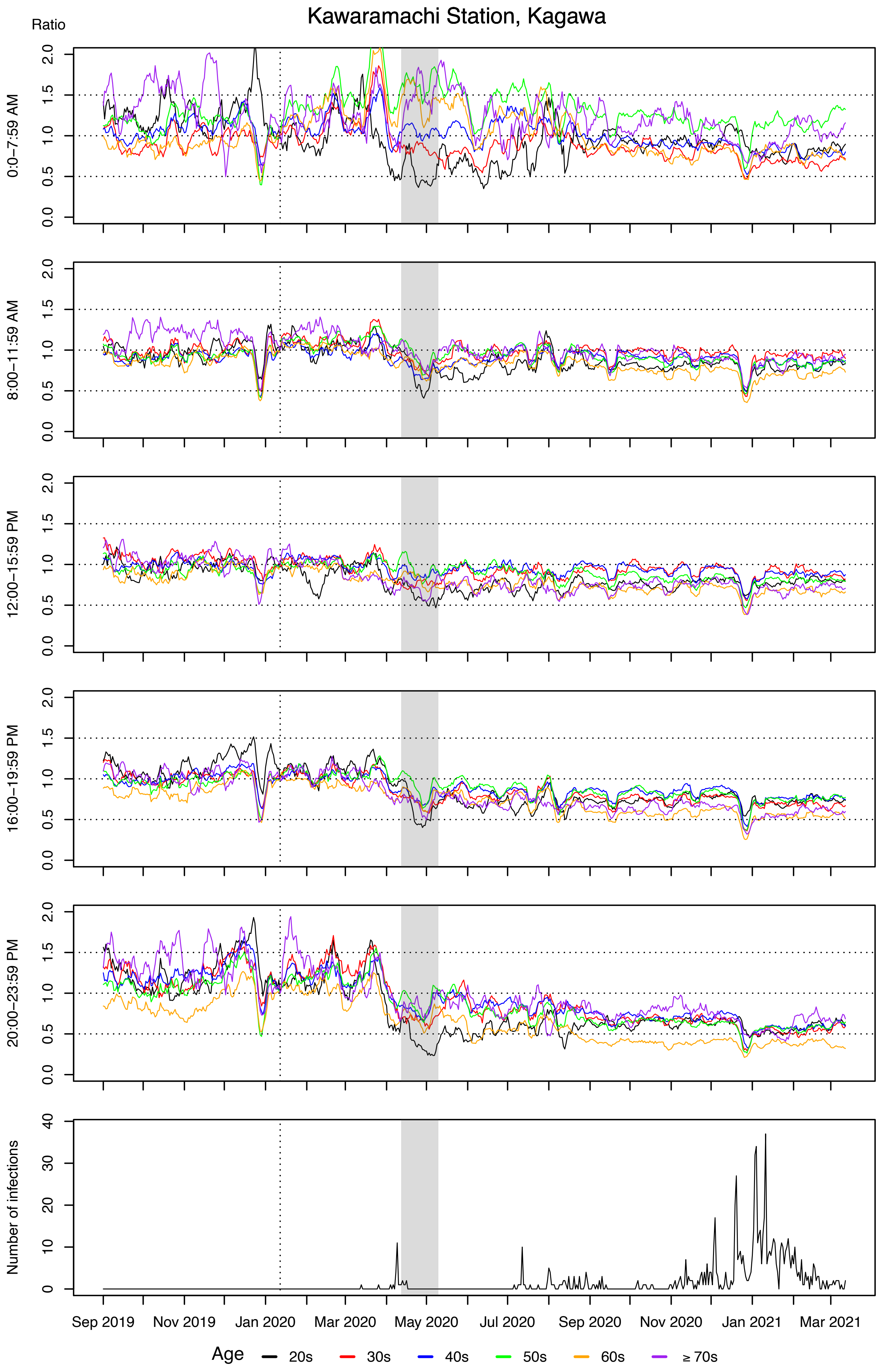

Kawaramachi Station, Kagawa
Ratio
0:0−7:59 AM
8:00−11:59 AM
12:00−15:59 PM
16:00−19:59 PM
20:00−23:59 PM
Number of infections
Sep 2019
Nov 2019
Jan 2020
Mar 2020
May 2020
Jul 2020
Sep 2020
Nov 2020
Jan 2021
Mar 2021
Age
20s
30s
40s
50s
60s
≥ 70s

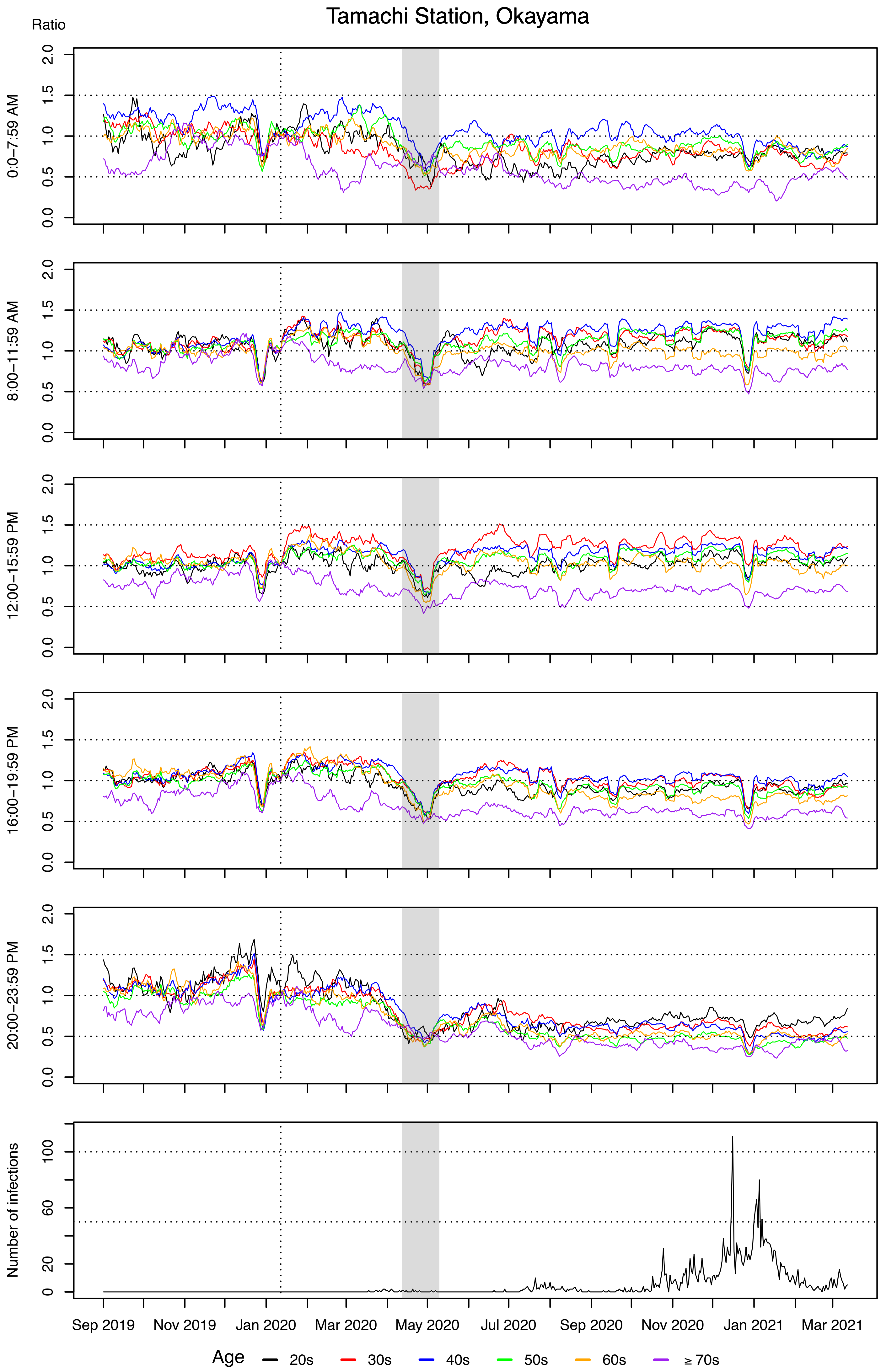

Tamachi Station, Okayama
Ratio
0:0−7:59 AM
8:00−11:59 AM
12:00−15:59 PM
16:00−19:59 PM
20:00−23:59 PM
Number of infections
0.0
0.5
1.0
1.5
2.0
0
20
60
100
Sep 2019
Nov 2019
Jan 2020
Mar 2020
May 2020
Jul 2020
Sep 2020
Nov 2020
Jan 2021
Mar 2021
Age
20s
30s
40s
50s
60s
≥ 70s

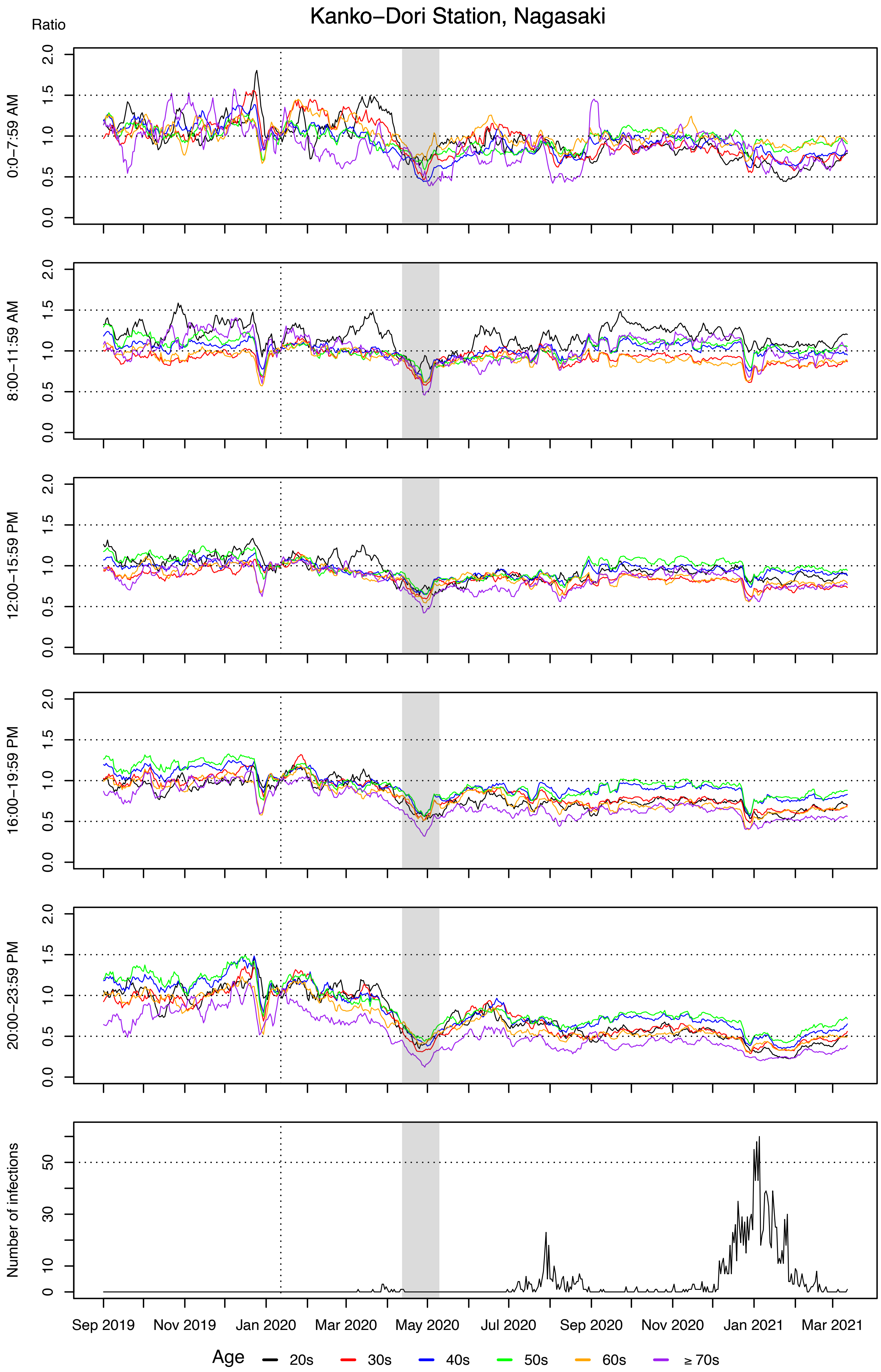

Kanko−Dori Station, Nagasaki
Ratio
0:0−7:59 AM
8:00−11:59 AM
12:00−15:59 PM
16:00−19:59 PM
20:00−23:59 PM
Number of infections
Sep 2019
Nov 2019
Jan 2020
Mar 2020
May 2020
Jul 2020
Sep 2020
Nov 2020
Jan 2021
Mar 2021
Age
20s
30s
40s
50s
60s
≥ 70s

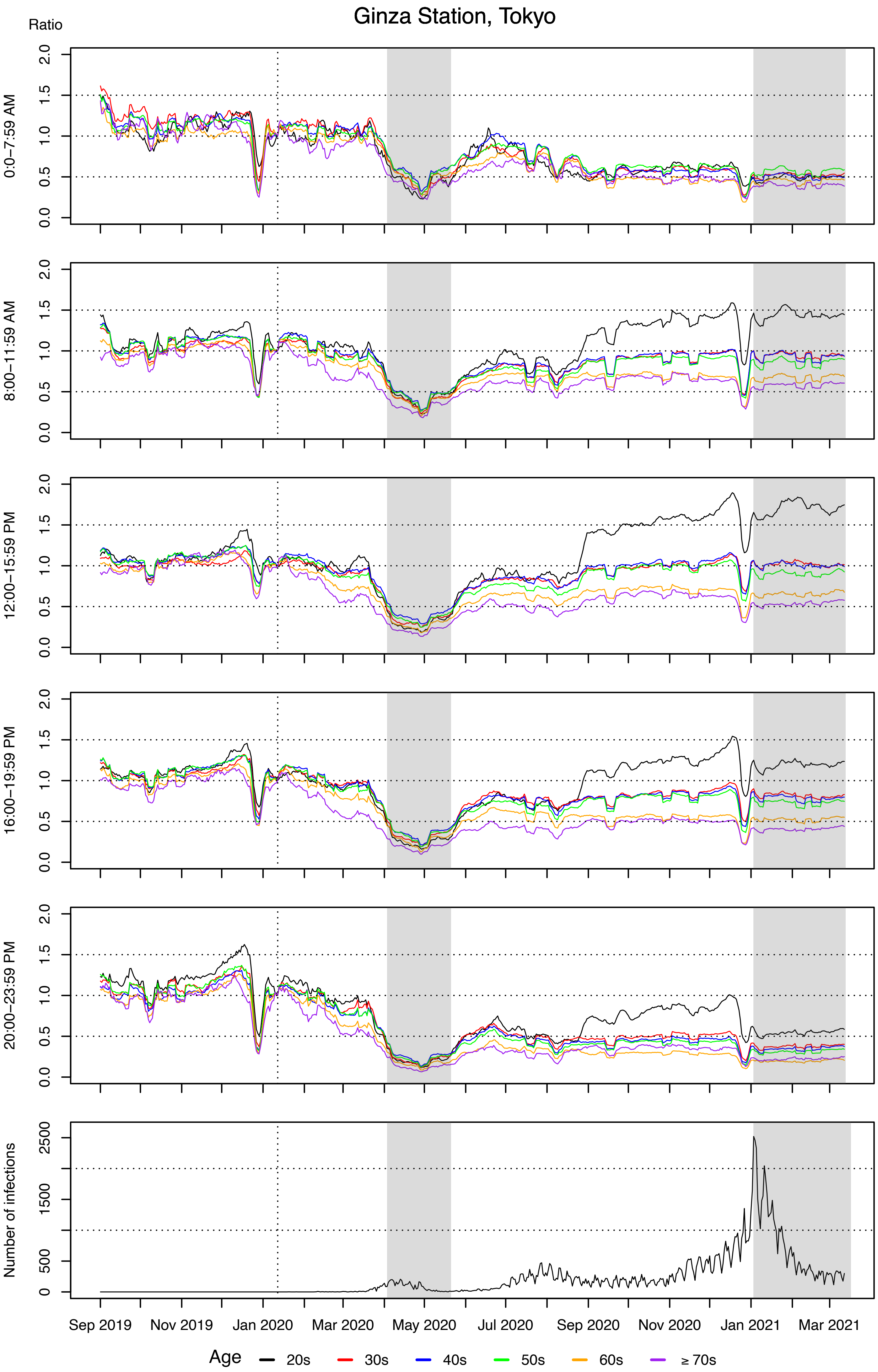

Ginza Station, Tokyo
Ratio
0:0−7:59 AM
8:00−11:59 AM
12:00−15:59 PM
16:00−19:59 PM
20:00−23:59 PM
Number of infections
0.0
0.5
1.0
1.5
2.0
0
500
1500
2500
Sep 2019
Nov 2019
Jan 2020
Mar 2020
May 2020
Jul 2020
Sep 2020
Nov 2020
Jan 2021
Mar 2021
Age
20s
30s
40s
50s
60s
≥ 70s

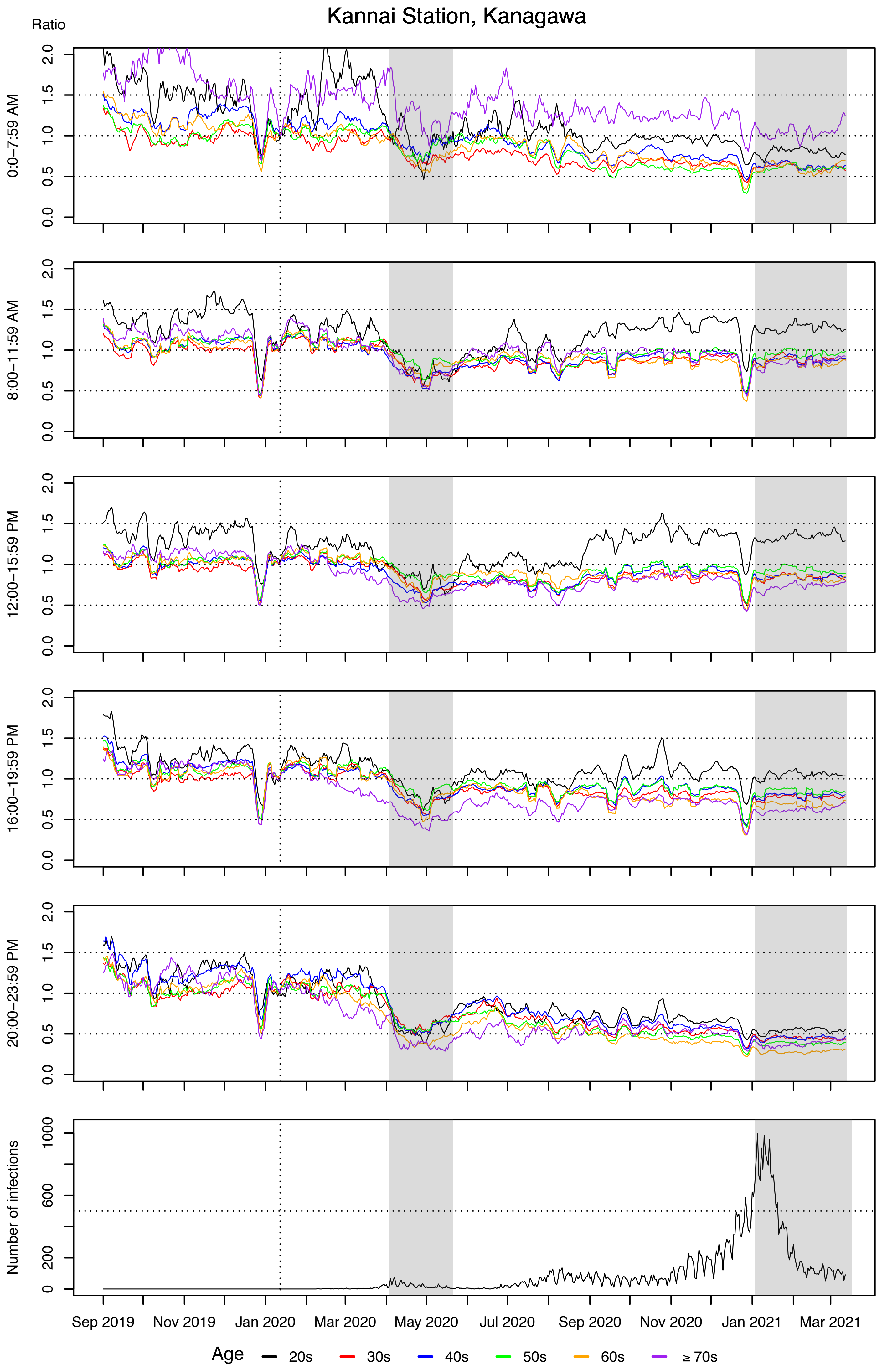
Kannai Station, Kanagawa
Ratio
0:0−7:59 AM
8:00−11:59 AM
12:00−15:59 PM
16:00−19:59 PM
20:00−23:59 PM
Number of infections
0.0
0.5
1.0
1.5
2.0
0
200
600
1000
Sep 2019
Nov 2019
Jan 2020
Mar 2020
May 2020
Jul 2020
Sep 2020
Nov 2020
Jan 2021
Mar 2021
Age
20s
30s
40s
50s
60s
≥ 70s
